# Metformin for the treatment of breast cancer: a scoping review of randomized clinical trials

**DOI:** 10.1186/s12885-025-14468-3

**Published:** 2025-08-21

**Authors:** Carolina Fumico Massuda Araujo, Lélia Cápua Nunes, Cristiane Murta-Nascimento, Arinilda Campos Bragagnoli, Fernanda Bono Fukushima, Cristiano de Pádua Souza, Edison Iglesias de Oliveira Vidal

**Affiliations:** 1 Public Health Department, Medical School, São Paulo State University (UNESP), Botucatu, SP Brazil; 2 Barretos Cancer Hospital, Barretos, SP Brazil; 3 Surgical Specialties and Anesthesiology Department, Medical School, São Paulo State University (UNESP), Botucatu, SP Brazil; 4 Internal Medicine Department, Medical School, São Paulo State University (UNESP), Av. Mario Rubens Guimaraes Montenegro, SN, Botucatu, SP 19818-687 Brazil; 5 Medicine Department, Science of Life Institute, Juiz de Fora - Campus Governador Valadares Federal University (UFJF-GV), Governador Valadares - MG, Brazil

**Keywords:** Metformin, Breast cancer, Review, Clinical trial

## Abstract

Metformin has been the focus of substantial interest in the field of oncology. Although breast cancer is the type of cancer where metformin was most extensively studied through randomized clinical trials (RCTs), none of the previous reviews in this field provided a comprehensive overview of the landscape of RCTs taking into account the phenotype of breast cancer, its staging, and treatment modalities. This scoping review sought to comprehensively map the literature of RCTs focusing on the use of metformin in the treatment of breast cancer and followed the Preferred Reporting Items for Systematic Review and Meta-Analysis (PRISMA) Extension for Scoping Reviews guidelines. The eligibility criteria encompassed all RCTs involving metformin for adult patients with breast cancer, with no constraints regarding context, language, publication date, or outcomes. We included 122 reports from 40 RCTs comprising a total of 5,623 participants and 107 distinct outcomes. The results showed that most studies did not present results by phenotype of breast cancer and highlighted critical gaps and opportunities in the literature. Notably, limited evidence from subgroup analyses within a large RCT suggested potential benefits of metformin in improving overall and disease-free survival among HER2 + participants but not among patients with other phenotypes, a result that requires further investigation. Our findings highlight the potential for considerably expanding the current knowledge base in this field through the retrospective determination of participant phenotypes, facilitating cost-effective and time-efficient individual participant data meta-analyses. Furthermore, we recommend that funding agencies and journals mandate the comprehensive presentation of results from RCTs on breast cancer based on phenotype.

**Supplementary Information:**

The online version contains supplementary material available at 10.1186/s12885-025-14468-3.

## Introduction

According to the most recent Global Cancer Statistics report, female breast cancer was the second leading cancer worldwide in 2022, accounting for 11.6% of all cancer cases, an estimated 2.3 million new cases, and 666,000 deaths [1]. Breast cancer accounts for approximately one in four cancer cases and one in six cancer deaths among women in the world. In addition, there are predictions for the year 2040 that the burden of breast cancer will increase to more than 3 million new cases and 1 million deaths every year due to population growth and aging alone [2].

Breast cancer treatments are expensive, and chemotherapy treatments are associated with major toxicity. In the USA, breast cancer has the highest treatment cost of any cancer, accounting for 14% of all cancer treatment costs [3]. In 2020, the cost of medical services for breast cancer patients was $26.2 billion, with an additional $3.5 billion spent on prescription drugs. Furthermore, chemotherapy is a common treatment for breast cancer and has been shown to improve survival. However, it often leads to adverse effects, including nausea, dysgeusia, peripheral neuropathy, loss of appetite, myalgia, and peripheral edema, which can significantly impact various aspects of quality of life [4]. Due to the high cost and toxicity associated with various treatments, less aggressive and more affordable breast cancer treatments are desirable.

Metformin is one of the most common medications used worldwide and has been used for more than 60 years because of its efficacy and safety [5]. It is a synthetic biguanide often prescribed as the first-line drug to treat type 2 diabetes mellitus (T2DM). In addition, it is an extremely inexpensive medication, costing approximately 15 cents per tablet [5]. Currently, it is used daily by more than 200 million diabetic patients around the world as monotherapy or in combination with other medications. Nevertheless, the precise mechanisms responsible for its therapeutic benefits are still not fully understood [6].

There are several public health interests in the repurposing of generic drugs for new therapeutic targets, as it represents a unique and cheaper strategy of innovation with several advantages compared with the long and costly process of developing new drugs from scratch [7]. With their initial indication, generic drugs have already passed all the phases required by regulatory agencies to be approved for commercialization. They have well-established pharmacodynamic and pharmacokinetic profiles with well-mapped adverse effects.

Over the past decades, besides treating T2DM, several other beneficial effects of metformin have been identified, such as preventing diabetes, and treating polycystic ovarian syndrome [8]. Importantly, metformin has shown promising effects against certain types of cancers and is being investigated in several studies, including observational studies [9], in vitro and/or in vivo experimental studies [10], and clinical trials [11].

The number of Randomized Clinical Trials (RCTs) of metformin for the treatment of breast cancer has been growing, but the landscape of this field remains unclear [12]. Moreover, breast cancer is a heterogeneous disease with great variation in its morphological and molecular characteristics, as well as in its clinical response. Furthermore, different stages of breast cancer are associated with distinct treatment modalities, which are associated with a variety of specific outcomes.

Importantly, a recent systematic review attempted to assess the evidence from RCTs on the effectiveness of metformin in the treatment of breast cancer [11]. However, that study was restricted to a limited range of outcomes and did not consider the different phenotypes, stages of breast cancer, or treatment modalities examined in the original studies. These factors are central to the appropriate interpretation of the effectiveness of any breast cancer treatment.

To date, metformin does not have an established role in breast cancer therapy. Given the sources of clinical heterogeneity underlying breast cancer and the many clinical trials conducted or planned to be initiated in this field, a scoping review is needed to show the extent of the landscape of existing studies and inform future directions and opportunities for research. Therefore, our goal with this scoping review was to map the literature on RCTs of metformin for the treatment of breast cancer.

## Methods

### Registration of the scoping review protocol

This scoping review was conducted according to the recommendations of the Joanna Briggs Institute guidance for systematic scoping reviews [13]. We registered the protocol with the Open Science Framework [14] (osf.io/yquba), and we published it elsewhere [15].

### Scoping review questions

We pursued answers to the following research questions:

1. What is the extent of the randomized clinical trials literature on the use of metformin in the treatment of breast cancer?
2. What phenotypes and stages of breast cancer were examined in those studies?
3. What treatment modalities, regimens, and comparators were used in those studies?
4. What outcomes were evaluated in those studies and what were their main findings?

### Eligibility criteria

We delineated the eligibility criteria following the ‘population, concept, and context’ (PCC) framework.

### Population

We included 18-year-old adult patients of both sexes with any phenotype and stage of breast cancer. We considered the reports to belong to the same study when the investigated population was the same. We considered those studies that used a subset of the population of a larger study as substudies.

### Concept

We accepted RCTs that included any intervention for treating breast cancer using metformin. Regarding the unit of randomization of the trials, we included both individuals and clusters of individuals in the population. We included trials in which patients were treated with metformin either alone or in combination with other systemic pharmacological treatments (e.g., hormone therapy or chemotherapy), local radiological treatments, surgical procedures, or behavioral interventions such as weight loss, exercise training, or nutritional interventions.

We included studies that used a placebo, standard treatment, or other behavioral interventions as long as they did not include metformin as a comparator/control. Those studies involving the use of metformin combined with other treatments must include a comparator using the same treatments without metformin so that the drug effect could be identified. For those studies that investigated metformin alone, we also included control groups with no treatment. We did not limit the outcomes evaluated in the selected studies. Specifically, we intended to map the outcomes of the clinical trials that proposed the use of metformin for breast cancer treatment.

### Context

There were no restrictions related to the context, language, or date of publication of the studies identified to be included in the scoping review.

### Literature search

On April 2021, we searched the following databases for potential studies: MEDLINE through PubMed, EMBASE, LILACS, Web of Science, and CENTRAL. The electronic search strategies are shown in Supplement 1. Additionally, we conducted a gray literature search in two databases: the System for Information on Grey Literature in Europe (OpenGrey) and the National Library of Medicine Bookshelf.

In addition to looking for relevant studies in databases, we also searched some registers. We screened ClinicalTrials.gov and the WHO International Clinical Trials Registry Platform (ICTRP).

We further extended our search efforts through other methods. We searched websites via Google Scholar, and we also hand-searched reference lists of relevant publications, conference abstract books, and specialist referrals.

Finally, during the analysis of the data in this review, we updated our search for references in September 2023 and verified whether the results of ongoing studies included in this review had already been published.

### Selection of studies

The study selection process followed careful identification steps. First, two researchers (CFMA and LCN) independently screened and reviewed the titles and abstracts of all records indexed in the databases using Rayyan software [16]. Second, they separately examined the full versions of the selected records from the first step. Third, the reviewers independently selected the first 200 results found via Google Scholar through Publish or Perish software [17] and screened them using the Rayyan software again. Finally, the investigators hand-searched the included reports as they extracted data for the review. A third reviewer (EIOV) resolved any disagreements that arose between the first two reviewers during the process.

### Data charting process

We developed a data extraction form to collect information from the included studies. We subsequently refined the standardized form according to the progress of the data charting process. All the extractions were performed independently and in duplicate by the two reviewers. Disagreements about the extracted data were resolved by discussion and consensus, and an independent third reviewer (EIOV) was consulted when conflicts and doubts persisted. We did not appraise the methodological quality of the included articles in alignment with the methodological expectations for scoping reviews.

Extracted data included: first author; study title; reports used for the data extraction; complete reference; is this a secondary report from a larger study?; the time period when the study was conducted; the geographical location where the study took place; study design; is the study protocol available?; type and frequency of breast cancer phenotypes, type and frequency of histological subtypes, type and frequency of other genetic characteristics (e.g. single nucleotide polymorphisms [SNP]); type and frequency of breast cancer stage under investigation; were other types of cancer included?; inclusion/exclusion criteria; sample size; characteristics of the population (e.g., mean age, mean Body Mass Index (BMI), frequency of obesity/overweight, menopause, physical activity, presence of comorbidities such as diabetes, hypertension, hyperlipidemia, and metabolic syndrome, frequency of treatment line chemotherapy provided in palliative care); details of the interventions, including treatment modality (i.e., neoadjuvant or adjuvant; palliative, or unclear), metformin dose, frequency and duration of metformin treatment, cointerventions; details of comparators, including type, dose, frequency, and duration; follow-up; outcome measures with the definitions used by the study authors; statistical analyses; results; adverse events; conclusions reported by the study authors; research limitations; funding sources; and references cited in studies’ reports to be evaluated for possible inclusion in our review. In addition, there was a field for free registration of other information deemed relevant by the reviewers.

Published articles with results were chosen to guide the full filling of extraction forms because they show the most recent and complete data related to the study. Reports such as study protocols and conference abstracts supported the charting of the data. Commonly, researchers make the results of their studies available in more than one article. To better organize one study’s data that are spread across multiple published articles, we filled out one form per article with results. When no published articles reported the results of included studies, we used data from study protocols and/or abstracts presented in conference proceedings to complete the extraction forms.

We stored the data extracted from each study in digital word-processing documents. At the same time, we organized the same data in digital spreadsheets in a summarized format while filling out the standardized forms. This approach facilitated a comprehensive overview of all studies and allowed for effective data synthesis.

### Collating, summarizing and reporting the results

We structured the presentation of our results around the different phenotypes of breast cancer, their stages, treatment modalities, types of interventions with metformin, comparators, outcomes evaluated in the primary studies, and their main findings. Because none of the studies whose population was restricted to participants with estrogen and/or progestogen receptor positive breast cancer presented their results separated by Luminal A and B phenotypes, we presented those studies under a single Luminal phenotype category. We constructed figures to present the results of the most relevant clinical outcomes evaluated by more than one included study. For these figures, we adopted the criterion of signaling statistically significant results favoring either the metformin group or the control group if at least one aspect of the outcome assessed by the study was positive. For example, if a significantly better overall survival (OS) was observed for patients with the HER2 + phenotype of breast cancer taking metformin in comparison to placebo, but no difference was observed for patients with other phenotype subgroups, the OS outcome was marked as favoring the metformin group for that study in the figure dedicated to that specific outcome.

The reporting of results was guided by the PRISMA-ScR statement (Preferred Reporting Items for Systematic Reviews and Meta-Analyses extension for Scoping Reviews) [18]. Our PRISMA-ScR checklist comprising our review is available in Supplement 2.

## Results

### Selection of studies and reports

**Fig. 1:**
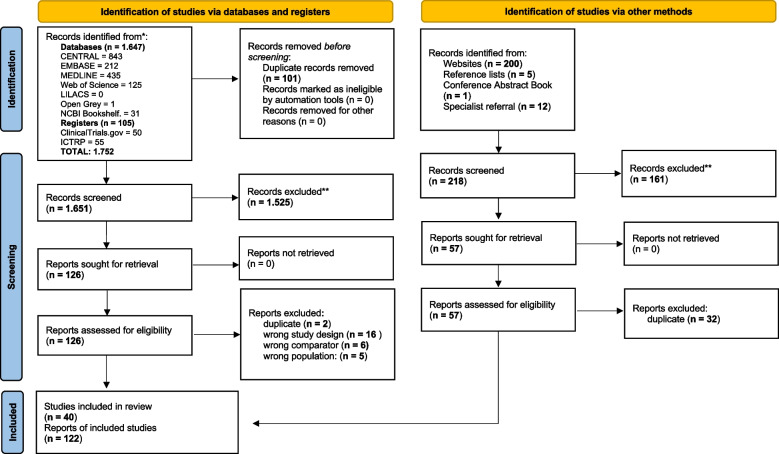
Flow diagram of included studies in the scoping review

We identified 40 studies based on eligibility criteria outlined by our PCC framework. Our searches identified 122 reports related to the 40 studies that were included in the scoping review after screening titles and abstracts and examining full versions of potentially relevant studies. Figure 1 outlines the flowchart for inclusion of reports in this review [19]. Supplement 3 lists the reports that were evaluated in full and excluded from databases, the ICTRP, and Google Scholar, along with the reasons for their exclusion. The reasons for exclusion were based on incompatibilities with our previously mentioned eligibility criteria. These included duplicate studies, incorrect study designs, mismatched populations, and incorrect comparators.

### Nature of reports

Of the 122 reports, 39 were full articles, 42 were protocols, 37 were conference abstracts, two were statistical analysis plans (SAP), only one was a thesis related to the study with the protocol number EudraCT 2007–000306-70 [20], and only one was a feasibility study whose results were available in the European Union Clinical Trials Registry (EUCTR) [21]. In addition, our review encompassed four large studies, each of which included several substudies: the MYME Trial [22, 23], the METTEN Trial [24–27], the NCIC CTG MA.32 (National Cancer Institute of Canada—Clinical Trials Group MA.32) [28–34], and a trial with the protocol number EudraCT number 2008–004912-10 [35–38].

### Availability of studies’ results

The dissemination of results from the included studies exhibited a heterogeneous and often incomplete pattern. Out of the 40 identified studies, 25 had some form of published results, with 19 [26, 28, 32, 39, 43–57] of them appearing as full articles and six [21, 54–58] having results published solely in the form of conference abstracts. In contrast, 15 of the included studies did not have published results; for 11 of them, only their protocols were available [59–69], and four were terminated prematurely without their results being published [70–73].

In addition, the characteristics of the included studies with published results are available in Table 1. Similarly, the characteristics of the included studies without published results are available in Supplement 4.

**Table 1:** Main characteristics of the 25 included studies with some form of published results

**Study name/First author/Year**	**Participants**	**Cancer (type, stage, treatment modality)**	**Intervention and Comparator**	**Outcomes**	**Main Results**
**Studies with Results Published as Full Text Articles**					
Ahmed, 2021	50 women with breast cancer No data on: diabetes, obesity, overweight, metabolic syndrome, and physical activity Menopausal status: Pre-menopause: 34 (68%); Post-menopause: 14 (28%)	**Phenotype**: 34 (68%) with luminal; 10 (20%) with HER2 +; 6 (12%) with triple negative breast cancer (TNBC) **Stage**: 80% with stage IIIA and 16% with stage IIIB **Treatment Modality**: neoadjuvant	**Interventions**: Standard neoadjuvant Adriamycin-Paclitaxel plus Metformin 500 mg twice/day until the time of surgery **Comparator**: standard neoadjuvant Adriamycin-Paclitaxel until the time of surgery	**Primary**: Pathological Complete Response (pCR) in the axilla and breast postoperatively **Secondary**: clinical response, method of surgery, toxicity, and Disease-free survival (DFS)	**pCR:** Metformin group: 15 (60%) Control group: 9 (36%) *p* = 0.08 **Clinical response** (total clinical staging remission post neoadjuvant chemotherapy): Metformin group: 76% Control group: 60% *p* = 0.39 **Method of surgery:** - Modified radical mastectomy: Metformin group: 19 (76%) Control group: 23 (92%) - Conservative surgery: Metformin group: 6 (24%) Control group: 2 (8%) *p* = 0.25 **DFS** (2-year DFS)**:** Metformin group: 91.3% Control group: 96.3% *p* = 0.57 **Radiological response:** “All patients achieved either complete remission or regressive disease” **Toxicity:** No differences in grade ≥ 3 adverse events between groups
EL-Haggar, 2016	A total of 148 women were eligible for the study. 129 women were randomized No data on: obesity, overweight, menopausal status, physical activity, and metabolic syndrome 100% nondiabetic women	**Phenotype**: Luminal: 86 (84.3%); Basal like: 10(9.8%); HER2 enriched: 6 (5.9%) **Stage**: Stage I: 16 (15.7%) Stage II: 33 (32.4%) Stage III: 53 (51.96%) **Treatment Modality**: adjuvant	**Interventions**: adjuvant therapy (chemotherapy treatment [CT] + hormonal therapy [HT]) + metformin treatment (850 mg of metformin twice daily) + Vitamin B12 (every 3 days) **Comparator**: adjuvant therapy (CT + HT) and Vitamin B12 (every 3 days)	**Primary**: DFS **Secondary**: Insulin-like Growth Factor (IGF)−1, Insulin-like Growth Factor Binding Protein (IGFBP)−3, IGF- 1: IGFBP-3 molar ratio, insulin, fasting blood glucose (FBG), Homeostasis Model Assessment of Insulin Resistance (HOMA-IR), Cancer Antigen 15–3 (CA 15–3) and metastasis	**DFS:** Metformin group: [mean survival time = 24.9 months, 95% confidence interval (CI): 24.1 to 25.7] (p = 0.04) Control group: [mean survival time = 22.8 months, 95%CI: 21.2 to 24.4] After adjusting for age, tumor stage, adjuvant chemotherapy, ER and HER2 status, control women were significantly more (p = 0.023) likely to develop metastasis than those treated with metformin [Hazard Ratio (HR) = 3.27, 95%CI: 1.17 to 9.06]
Ko, 2015	105 women with breast cancer No data on: metabolic syndrome Diabetes: 1% Obese/overweight: 100% Menopause: 85% a 88% Regular exercise: 51% to 69%	**Phenotype**: 17% HER2 +; 62% luminal; 16% triple negative; 5% not specified **Stage**: 36% of patients were stage 0 or 1 **Treatment Modality**: adjuvant	**Interventions**: Metformin 500 mg/d group: Received metformin 500 mg dose once daily for 2 weeks and then placebo was added to that regimen for 22 weeks Metformin 1000 mg/d group: Received metformin 500 mg dose once daily for 2 weeks and then the dose was escalated to 500 mg twice daily for 22 weeks **Comparator**: Placebo	**Primary**: Weight loss **Secondary**: FBG, glycated hemoglobin (HbA1c), insulin levels; total cholesterol, HDL and LDL-cholesterols, and triglyceride levels; Anthropometry (i.e., weight, height, BMI, waist circumference, and blood pressures) and body composition. Beck depression inventory (BDI)	**Weight loss (kg):** Metformin 500 mg group [mean (Standar Error (SE)]: −0.5 (0.3) Metformin 1000 mg group:—0.6 (0.2) Placebo group: −1.1 (0.3) *p* = 0.22 **Glucose (mg/dL):** Metformin 500 mg group: 88.9 (1.4) Metformin 1000 mg group: 88.7 (2.3) Placebo group: 90.6 (1.0) *p* = 0.79 **Insulin (µIU/mL):** Metformin 500 mg group: 8.3 (0.4) Metformin 1000 mg group: 7.2 (0.3) Placebo group: 8.1 (0.4); *p* = 0.57 **HbA1c (%):** Metformin 500 mg group: 5.84 (0.04) Metformin 1000 mg group: 5.68 (0.07) Placebo group: 5.82 (0.05) p = 0.75 **Total cholesterol (mg/dL):** Metformin 500 mg group: 193.2 (6.0) Metformin 1000 mg group: 190.1 (5.5) Placebo group: 188.4 (7.1) *p* = 0.91 **BMI (kg/m**^**2**^**):** Metformin 500 mg group: 26.5 (0.5) Metformin 1000 mg group: 26.9 (0.5) Placebo group: 26.9 (0.5) *p* = 0.50 **Waist (cm):** Metformin 500 mg group: 86.8 (1.3) Metformin 1000 mg group: 87.4 (1.1) Control group: 84.2 (1.3) *p* = 0.32 **Beck depression inventory (BDI):** Metformin 500 mg group: 10.3 (1.0) Metformin 1000 mg group: 10.5 (1.0) Placebo group: 10.4 (1.3) *p* = 0.81
PECAM Study (Davis, 2018)	105 women with breast cancer No data on: obesity, overweight, physical activity, and metabolic syndrome 100% postmenopausal women 100% nondiabetic women	**Phenotype**: 100% ER +/PR + (inclusion criteria) **Stage**: stage I/II **Treatment Modality**: adjuvant	**Interventions**: The metformin dose was titrated over three weeks from 425 mg at night to 850 mg twice a day **Comparator**: Placebo will be identical tablets to the metformin 850 mg tablets	**Primary:** Endometrial Thickness (ET) **Secondary**: Clinical characteristics (BMI, weight, waist circumference) and biochemistry (FBG, fasting insulin, HOMA-IR)	**ET:** Metformin group [median (range)]: 2.3 mm (1.4 to 7.8) Placebo group [median (range)]: 3.0 mm (1.2 to 11.3) *p* = 0.05 **BMI change:** Metformin group [median (range)]: −0.58 (−2.96 to 1.37) Placebo group [median (range)]: 0.52 (−2.27 to 2.90) *p *< 0.001 **Weight change (Kg):** Metformin group [median (range)]: −1.7 (−8.4 to 3.7) Placebo group [median (range)]: 1.5 (−6.1 to 7.4) *p* < 0.001 **Waist circumference change (cm):** Metformin group [median (range)]: −1 (−13 to 5) Placebo group [median (range)]: 1 (−12 to 14) *p *= 0.06 **FBG change (mmol/L):** Metformin group [median (range)]: −0.25 (−1.3 to 0.6) Placebo group [median (range)]: 0.0 (−0.5 to 0.7) *p* < 0.001 **Insulin change (μU/mL):** Metformin group [median (range)]: −0.5 (−7.9 to 4.5) Placebo group [median (range)]: 0.5 (−2.7 to 9.8) *p *< 0.01 **HOMA-IR change:** Metformin group [median (range)]: −0.17 (−2.17 to 0.96) Placebo group [median (range)]: 0.14 (−0.71 to 2.48) *p* < 0.001
Liubota, 2018	72 women included No data on: physical activity and diabetes 100% obese and 100% with metabolic syndrome Premenopausal status: 12 (16,7%); Postmenopausal status: 60 (83,3%); Hypertension: 39 (54,2%); Hyperlipidemia: 61 (84,7%) Hiperlipidemia 61 (84.7%)	**Phenotype**: Luminal: 52 (72,22%); HER2 + : 6 (8,33%); TNBC: 14 (19,44%) **Stage**: Stage II: 35 (48,61%); Stage III: 37 (51,39%); **Treatment Modality**: neoadjuvant	**Interventions**: 500 mg metformin 30 min before meal, three times a day with standard neoadjuvant treatment **Comparator**: standard neoadjuvant treatment	**Primary:** Clinical Response Rate [Complete Clinical Response (cCR), Partial Clinical Response (cPR), Clinical Stable Disease (cSD) and Clinical Progressive Disease (cPD)] and pCR **Secondary**: Type of surgery performed, DFS, Overall survival (OS)	**Clinical Response Rate**: Overall clinical response rate (cCR + cPR) Metformin group: 28 (77,5%) Control group: 9 (25%) *p* < 0,05 **Pathological complete response**: Metformin group: 9 (26,5%) Control group: 2 (6%) *p* < 0.05 **Type of surgery performed**: - Breast-conserving surgery: Metformin group: 9 (50%) Control group: 4 (23,5%) *p* < 0.05 - Radical mastectomy: Metformin group: 7 (38%) Control group: 13 (76.5%) **DFS** (5-year DFS)**:** Metformin group: 57% Control group: 58% *p* = 0.79 **OS** (5-year OS): Metformin group: 76% Control group: 59% *p* = 0.67
Salah, 2021	50 women included No data on: obesity/overweight, physical activity and metabolic syndrome Pre-menopausal: 22 (44%) Post-menopausal: 28 (56%) 100% nondiabetic women	**Receptors**: Overall HER 2: HER2-: 36 (72%); HER2 + : 14(28%) Overall HR: ER/PR-: 10 (20%); ER/PR + = 40 (80%) **Stage**: 100% stage IV **Treatment Modality**: adjuvant	**Interventions**: Standard chemotherapy plus metformin 1 g twice daily (2 g/day) **Comparator**: Standard chemotherapy	**Clinical outcomes:** **Primary:** Radiologic Response Rate **Secondary:** OS and progression-free survival (PFS)	**Radiologic Response Rate:** Women in metformin group had significantly better radiologic response rate than women in the control group (p = 0.002) **OS:** Metformin group [mean OS]: 5.8 months Control group [mean OS]: 5.3 months (HR = 0.57, 95%CI: 0.24 to 1.3) **PFS:** Metformin group [mean PFS]: 5.1 months Control group [mean PFS]: 4.4 months (HR = 0.311, 95%CI: 0.063 to 1.5)
Barakat, 2022	80 women included; (74 randomized) No data on: physical activity and metabolic syndrome Obesity: 32 (43,2%) Overweight: 26 (35,1%) Premenopause: 38 (51,4%) Postmenopause: 36 (48,7%) 100% nondiabetic	**Phenotype**: Luminal A: 30 (40,54%) Luminal B/HER2-: 8 (10,81%) Luminal B/HER2 + : 25 (33,78%) HER2 +/non-luminal: 5 (6,76%) TNBC: 6 (8,11%) **Stage**: IIB: 10 (13,51%) IIIA: 31 (41,89%) IIIB: 33 (44,59%) **Treatment Modality**: neoadjuvant	**Interventions**: Standard Neoadjuvant Chemotherapty (NACT) (4 cycles doxorubicin 60 mg/m2/cyclophosphamide 600 mg/m2, followed by 12 cycles of weekly paclitaxel 80 mg/m2) + Metformin (1000 mg twice daily) followed by surgery **Comparator**: Standard NACT only followed by surgery	**Primary:** Objective response rate (ORR), cCR and pCR **Secondary:** Breast conservative rate (BCR), safety profile and tolerability and quality of life (QoL)	**ORR:** Metformin group: 29 (80.6%) Control group: 26 (64.4%) (OR = 1.91, 95%CI: 0.66 to 5.59, p = 0.24) **cCr:** Metformin group: 10 (27.8%) Control group: 4 (10.5%) (OR = 3.27, 95%CI: 0.92 to 11.60, p = 0.06) **pCR:** Metformin group: 8 (22.2%) Control group: 4 (10.5%) (OR = 2.43, 95%CI: 0.66 to 8.91, p = 0.18) **BCR:** Metformin group: 7 (19.4%) Control group: 5 (13.2%) (OR = 1.59, 95% CI: 0.46 to 5.57, p = 0.47) **QoL:** QoL was similar in the metformin and control groups across different QoL domains **Safety and tolerability:** Grade I and II gastrointestinal symptoms were more common in the metformin group but chemotherapy-induced peripheral neuropathy was less common in the metformin group than in the control group
Zhao, 2017	60 patients No data on: obesity, overweight, physical activity, diabetes, and metabolic syndrome 100% postmenopausal	**Phenotype**: Luminal = 60 (100%); **Receptors**: 100% HR + (ER and/or PR), 100% HER- **Stage**: Stage III and IV: 60 (100%) **Treatment Modality**: adjuvant and palliative	**Interventions**: aromatase inhibitor (exemestane 25 mg/d or letrozole 2.5 mg/d depending on the most recent treatment) plus metformin (0.5 g bid, orally) **Comparator**: Aromatase inhibitor (letrozole or exemestane) + placebo	**Clinical outcomes:** **Primary:** PFS **Secondary:** ORR, Clinical Benefit Rate (CBR), OS and tolerability	**PFS:** HR = 1.2, 95%CI: 0.7 to 2.1, p = 0.48 **ORR:** Metformin group: 6.7% (95%CI: 0.3 to 16) Control group: 0% (95%CI: not available) p = 0.99 **CBR:** Metformin group: 33.3% (95%CI: 15 to 51) Control group: 50.0% (95%CI: 31 to 69) (OR = 0.50, 95%CI: 0.2 to 1.4, p = 0.15) **OS:** HR = 1.1, 95%CI: 0.5 to 2.4, p = 0.81 **Tolerability:** No substantial difference in incidence or severity of adverse events was seen between the two treatment groups
METEOR Trial (Kim, 2019)	203 women were randomized. 153 intention-to-treat population were analyzed (72 metformin 75 placebo group) No data on: obesity, overweight, physical activity, and metabolic syndrome 100% postmenopausal 100% nondiabetic	**Phenotype**: Luminal = 203 (100%); **Receptors**: ER + = 203 (100%) **Stage**: 100% stages II/III. There is no information on specific frequencies for stage II and stage III separately **Treatment Modality**: neoadjuvant	**Interventions**: Metformin (2000 mg/day) + Letrozole (2.5 mg/day) **Comparator**: Placebo + Letrozole (2.5 mg/day)	**Primary:** clinical response rate (CRR) **Secondary:** pCR, breast conservation rate, breast density change, Ki67(%) and toxicity	**CRR:** Metformin group: 66.7% Control group: 56.4% *p *= 0.19 **pCR:** Not reported **Breast conservation rate:** Metformin group: 66.7% Control group: 69.3% *p* > 0.05 **Breast density change:** Not reported **Ki67 (%)** Neither Ki67% nor PEPI score was different between the two groups. However, among the 20 patients with core-needle biopsy after 4 weeks of medication, greater number of patients displayed Ki67 < 10% in the metformin group than in the placebo group (87.5% versus 33.3%, p = 0.017)
MBC1 Trial (Semiglazova, 2019)	53 patients were included in the study No data regarding: obesity, overweight, menopause status, physical activity, and metabolic syndrome 100% nondiabetic	**Phenotype**: Luminal, HER2, Triple negative **Receptors**: ER +/HER2-(68%); ER +/HER2 + (25%); ER-/HER2- (7%); **Stage**: stage IIB, IIIA,IIIB,IIIC (frequency not reported) **Treatment Modality**: neoadjuvant	**Interventions**: FDC × 6 cycles once every 21 days with metformin 850 mg BID **Comparator**: conventional chemotherapy (FDC)	**Primary:** ORR and pathomorphological response (pR) **Secondary:** Adverse events and QoL	**ORR:** Metformin group: 75% Control group: 85% **pR**: Metformin group compared to control group: OR = 0.602, 95%CI: 0.085 to 4.284, p = 0.612 **Adverse events:** Not reported **QoL:** Not reported
Semiglazova, 2018	54 patients (recruiting) No data regarding: physical activity, and metabolic syndrome Obesity: 23 (42%) Overweight: 15 (28%) 100% menopause 100% nondiabetic	**Phenotype**: Luminal **Receptors**: 54 (100%) ER + **Stage**: stage IIB, IIIA, IIIB, IIIC (frequency not reported) **Treatment Modality**: neoadjuvant	**Interventions**: Arm 3: Toremifene (TOR) 120 mg daily with metformin (MTF) 850 mg BID **Comparator**: Arm 1: 120 mg of oral toremifene (TOR) daily; Arm 2: Toremifene (TOR) 120 mg daily with 3 mg overnight melatonin	**Primary:** Ki67 index **Secondary:** ORR, pR (tumor and lymph nodes), adverse events and QoL	**Ki67 (decreased level)**: Metformin group: 74% Control group: 42% RR = 4.23, 95%CI: 1.044 to 17.139, p = 0.043 **Objective Response Rate:** Metformin group: 47.3% Control group: 31.6% RR not reported **pR:** A complete pR in the tumor and lymph nodes was not achieved in any patient **Adverse events:** Not reported **QoL:** There was not reduce the quality of life of patients
Reach for Health trial (Patterson, 2016; Patterson, 2018; Hartman, 2019)	333 participants No data regarding: diabetes and metabolic syndrome Comorbidities: High blood pressure = 166(49.9%); High cholesterol: 164 (49.3%); Previous smoking + current smoking = 149 (44,7%); Any current alcohol intake = 225 (67.6%) Phisical activity: median (Q1–Q3): 16.0 (2.7–42.0) Metabolic Equivalent Task (MET) hours per week (self-report) 100% overweight/obese 100% postmenopausal	**Phenotype**: ER + or PR + HER2- (Luminal): 240 (72.1%); HER2 + : 51 (15.3%); Triple-negative (ER-, PR-, HER2-): 30 (9.0%); Missing data: 12 (3.6%) **Stage**: Stage I: 161 (48.4%); Stage II: 116 (34.8%); Stage III: 56 (16.8%) **Treatment Modality**: prevention of recurrence after end of cancer treatment	**Interventions**: Metformin only (1500 mg/day) and Weight loss & Metformin **Comparator:** Placebo only and Weight loss & placebo	**Primary:** Fasting insulin, glucose, C-reactive protein (CRP), estradiol, testosterone, and sex-hormone binding globulin (SHBG) **Secondary:** Weight and five cognitive domains (exploratory outcome)	Insulin (pg/mL) change*: –7.88, 95%CI: –15.01 to –0.76, p = 0.03 Glucose (mg/dL) change*: –1.27, 95%CI: –3.39 to 0.85, p = 0.24 C-reactive protein (mg/L) change*: –14.87, 95%CI: –32.88 to 3.15, p = 0.10 Estradiol (pg/mL) change*: –9.98, 95%CI: –18.45 to –1.51, p = 0.02 Testosterone (ng/dL) change*: –9.49, 95%CI: –15.20 to –3.77, p = 0.001 SHBG (nmol/L) change*: 7.5, 95%CI: 2.43% to 12.57, p = 0.004 Geometric mean weight (Kg) change* –2.46, 95%CI: –3.59 to –1.34, p < 0.001 **Cognitive domains change (follow up-baseline):** Difference between metformin and control group: Visual spatial: 1.67, 95%CI:−0.92 to 4.25, p = 0.205 Verbal function: −1.58, 95%CI: −4.90 to 1.74, p = 0.351 Memory: 0.67, 95%CI: −1.09 to 2.43, p = 0.454 Executive function: 1.12, 95%CI: −1.20 to 3.44, p = 0.342 Attention: 0.70, 95%CI: −1.03 to 2.44, p = 0.425 *percent change in metformin group minus placebo group
NeoMet Trial (Hadad, 2011; Hadad, 2015)	47 participants No data regarding: obesity, overweight, physical activity, and metabolic syndrome Pre- and peri-menopausal: 2 (14.3%) Post-menopausal: 12 (85.7%) 100% nondiabetic	**Phenotype**: Luminal A and Luminal B **Receptors**: ER + : 11 (78.57%); PR + : 11 (78.57%); HER + : 2 (14.28%) **Stage**: stage I or II **Treatment Modality**: neoadjuvant	**Interventions**: metformin 2000 mg **Comparator:** No treatment	Ki67 index, insulin, gene array data, PDE3B and p53, ingenuity pathway analysis, gene set analysis, pAMPK, pAkt, insulin receptor signaling and caspase 3 Note: no clear information about which were the primary and the secondary outcomes	**Ki67** (Mean percentage change of cells staining for Ki67: preoperative-baseline)**:** Metformin group: −3.4 (p = 0.027) Control group: −0.03 (p = 0.455) **Insulin** (Mean percentage change: preoperative-baseline)**:** Metformin group: 0.87 (p = 0.663) Control group: 13.75 (p = 0.044) **Gene array data** Not reported to randomized groups **PDE3B and p53** Not reported to randomized groups **Ingenuity pathway analysis** Not reported to randomized groups **Gene set analysis** Not reported to randomized groups **pAMPK (allred)** (score change baseline to preoperative) Metformin group: from 5.18 to 6.45, p = 0.04 Control group: from 5.64 to 6.00, p = 0.492 **pAkt (allred)** (score change baseline to preoperative)**:** Metformin group: from 5.91 to 5.00, p = 0.043 Control group: from 4.91 to 5.18, p = 0.517 **Insulin receptor signaling (mmol/L) (allred)** (score change baseline to preoperative)**:** Metformin group: from 4.9 to 4.4, p = 0.363 Control group: from 4.09 to 4.36, p = 0.431 **Caspase-3 (%)** (percentage of cancer cell stained preoperative-baseline)**:** Metformin group: −0.292, p = 0.0437 Control group: −0.146, p = 0.371 PS: None of the changes in the biomarkers were associated with the patient’s BMI either using BMI as a continuous variable or with a cut off of 26 or 30
Meyerhardt, 2020; Brown, 2020	139 (87 with breast cancer; 52 with colorectal cancer) (62,6% BC/37,4% CRC) No characteristics were presented for breast cancer only No data regarding: obesity, overweight, menopause status, physical activity, and metabolic syndrome 100% nondiabetic	**Phenotype**: NI **Stage**: stage I to stage III **Treatment Modality**: adjuvant	**Interventions**: Metformin alone arm: starting dose of 850mg once daily for 2 weeks, followed by 850mg twice daily for 3 months. Exercise alone arm: two supervised exercise sessions per week for 3 months + recommendation of additional 120 minutes of exercise on their own per week. Metformin + exercise arm: combination of both arms above. **Comparator**: Educational information packet about nutrition and physical activity.	**Primary:** Fasting insulin **Secondary:** IGF-1, IGFBP-1, IGFBP-3, leptin, fasting glucose, weight, BMI, waist to hip ratio, high sensitivity CRP (hs-CRP), soluble tumor necrosis factor alpha receptor 2 (sTNF-αR2) and interleukin 6 (IL-6)	No results were reported for breast cancer only
SPIRIT Trial (Yeh, 2021; Hu, 2021)	121 participants Participants were cancer survivors with overweight or obesity Participants with different malignant solid tumors were included (breast = 68) No data regarding: menopause status, physical activity, diabetes, and metabolic syndrome 100% overweight or obesity	**Phenotype**: No information was reported specifically for breast cancer **Stage**: No information was reported specifically for breast cancer **Treatment Modality**: prevention of recurrence after end of cancer treatment	**Interventions**: Metformin 2000 mg/day. Dosing can be flexible, two or three times per day with meals as tolerated for 12 months **Comparator**: Two arms: Coach directed weight loss; Self-directed weight loss	**Primary:** IGF-1 (6-month) **Secondary:** IGF-1 (12 months), IGF-1: IGFBP3 molar ratio (6 and and 12 months), weight, BMI, fasting insulin, HbA1c, IL-6, and hs-CRP**,** fasting Serum Urate (SU), Glomerular Filtration Rate (eGFR) and BMI	No results were reported for breast cancer separately
Saif, 2019; Kritharis, 2014	A total of 100 patients of both sexes were enrolled (52 in delayed arm *vs*. 48 concurrent arm) It was included 16 different solid tumor types (breast = 11) No data regarding: obesity, overweight, menopause status, physical activity, diabetes, and metabolic syndrome	**Phenotype**: NI **Stage**: NI **Treatment Modality**: adjuvant	**Interventions**: metformin 500 mg twice daily (1 g daily) with chemotherapy **Comparator**: chemotherapy alone (Stage 1 of study)	**Primary:** Dose limiting toxicity (DLT) (Stage 1 of study: when metformin was added to chemotherapy) **Secondary:** Adverse events, tumor responses, PFS and pharmacodynamic markers	No results were reported for breast cancer separately
MYME Trial Nanni, 2018	126 participants 21 (17%) obese 50 (41%) overweight 100 (82%) post-menopausal 100% nondiabetic	**Phenotype**: Luminal **Receptors**: ER + : 106 (87%); PgR + : 87 (71%); HER2-: 100% **Stage**: Stage IV: 126 (100%) **Treatment line:** 100% first line chemotherapy **Treatment modality**: palliative	**Interventions**: Metformin 2000 mg/day + First-line chemotherapy alone (NPLD + cyclophosphamide) **Comparator**: First-line chemotherapy alone (NPLD + cyclophosphamide)	**Primary:** PFS **Secondary:** OS, ORR, PFS as function of HOMA Index levels, ORR as function of HOMA Index levels, OS as function of HOMA Index levels and Toxicity	**PFS:** Metformin group (median PFS): 9.4 months, 95%CI: 7.8 to 10.4 Control group (median PFS): 9.9 months, 95%CI: 7.4 to 11.5 (HR = 1.09, 95%CI: 0.75 to 1.58, p = 0.653) **OS:** Metformin group (median OS): 34.4 months, 95%CI: 19.3 to 37.2 Control group (median OS): 26.8 months, 95%CI: 19.4 to 37.9 (HR: 0.81, 95%CI: 0.50 to 1.30, p = 0.382) **ORR:** Metformin group: 48%, 95%CI: 32.0 to 63.5 Control group: 49%, 95%CI: 34.1 to 63.9, p = 0.901 **PFS as function of HOMA Index levels:** HOMA Index < 2.5 group, median PFS: 10.4 months, 95%CI: 9.6 to 11.7 HOMA Index ≥ 2.5 group, median PFS: 8.5 months, 95%CI: 5.8 to 9.7 (HR = 1.51, 95%CI: 1.03 to 2.20, p = 0.034) **OS as function of HOMA Index levels:** HOMA Index < 2.5 group (median OS): 30.8 months, 95%CI:19.4 to 41.4 HOMA Index ≥ 2.5 group (median OS): 27.2 months, 95%CI:19.3 to 37.0 (HR = 0.97, 95%CI: 0.61 to 1.55, p = 0.900) **ORR as function of HOMA**: Not reported **Toxicity:** Neutropenia grade 3/4: Metformin group: 54% Control group: 72% (*p* = 0.019) Febrile neutropenia: Metformin group: 1 patient Control group: 6 patients (*p* = 0.076) Diarrhea: Metformin group: 8% Control group: 0% (*p* = 0.014) There was no clinical evidence of cardiotoxicity in either arm
MYME Trial TransMYME Trial Gennari, 2020	126 participants (TransMYME study sample = 72) 100% nondiabetic	**Phenotype**: Luminal **Receptors**: ER + : 106 (87%); PgR + : 87 (71%); HER2-: 100% **Stage**: Stage IV: 126 (100%) **Treatment line:** 100% first line chemotherapy **Treatment modality**: palliative	**Interventions**: Metformin 2000 mg/day + First-line chemotherapy alone (NPLD + cyclophosphamide) **Comparator**: First-line chemotherapy alone (NPLD + cyclophosphamide)	**TransMYME trial:** prognostic role of insulin-like growth factor-1 receptor (IGF-1R) expression on circulating tumor cells (CTC) and prognostic role of CTC count and IGF-1R was assessed for PFS and OS	No interaction was detected between metformin treatment and the number of CTC or with the expression of IGF-1R on such cells in relation to PFS (p = 0.322) or OS (p = 0.840). There was no effect of metformin treatment on the evolution of the number of CTC or the expression of IGF-1R over time [These results were described only in a narrative form, without presenting the exact quantitative results]
METTEN Trial Martin-Castillo, 2018	84 patients were randomized, but results were presented for patients who received at least one dose of study medication (n = 79) 100% not obese (exclusion criteria: BMI > 30) Overweight: Metformin group: 17 (44.7%) Control group: 21 (51.2%) Postmenopausal Metformin group: 14 (36.8%) Control group: 17 (41.5%) Premenopausal Metformin group: 24 (63.2%) Control group: 24 (58.8%) 100% nondiabetic No data regarding: physical activity and metabolic syndrome	**Phenotype**: HER2 **Receptors**: **ER/PgR + ** Metformin group: 19 (50.0%) Control group: 24 (58.5%) **ER/PgR-:** Metformin group: 19 (50.0%) Control group: 17 (41.5%) **Stage**: Stage II—III **Treatment modality**: neoadjuvant	**Interventions**: Metformin 1.700 mg/day (850 mg BID) for 24 weeks concurrently with 12 cycles of weekly paclitaxel plus trastuzumab, followed by four cycles of 3-weekly fluorouracil, epirubicin, cyclophosphamide (FE75C) plus trastuzumab **Comparator**: Only equivalent sequential chemotherapy plus trastuzumab (paclitaxel plus trastuzumab, followed by fluorouracil, epirubicin, cyclophosphamide plus trastuzumab)	**Primary:** pCR **Secondary:** Tolerability and safety profile (including cardiac toxicity), the rate of breast conservation, 5-year DFS, the inhibition of tumor tissue biomarkers (including proliferative, mTOR/AMPK and HER2-related pathways), and changes in circulating levels of insulin and metabolites	**pCR (per protocol):** Metformin group: 65.5% had a pCR (95%CI: 47 to 80) Control group: 58.6% had a pCR (95%CI: 41 to 74) (OR = 1.34, 95%CI: 0.46 to 3.89, p = 0.588) **Cardiac toxicity (left ventricular ejection fraction):** Metformin group (median change from baseline): –4.0%, 95%CI: –6.0 to –1.8 (end of treatment) Control group (median change from baseline): –5.0%, 95%CI: –7.5 to –1.0 (end of treatment) (*p* = 0.409) **Rate of breast conservation (per protocol):** Metformin group: 79.3% of breast-conserving surgery Control group: 58.6% of breast-conserving surgery (*p* = 0.089) **DFS** Not reported **Changes in circulating levels of insulin and metabolites (analysis only PCR groups):** Not reported to intervention groups
METTEN Trial Lopez-Bonet, 2019	79 patients included in the intention-to-treat (ITT) population of the METTEN study 100% not obese (exclusion criteria: BMI > 30) 100% nondiabetic No data regarding: menopause status, physical activity, and metabolic syndrome	**Phenotype**: HER2 **Receptors**: **ER/PgR + ** Arm A with metformin: 19 (50.0%) Arm B control: 24 (58.5%) **ER/PgR-:** Arm A with metformin: 19 (50.0%) Arm B control: 17 (41.5%) **Stage**: Stage II—III **Treatment modality**: neoadjuvant	**Interventions**: Metformin 1.700 mg/day (850 mg BID) for 24 weeks concurrently with 12 cycles of weekly paclitaxel plus trastuzumab, followed by four cycles of 3-weekly fluorouracil, epirubicin, cyclophosphamide (FE75C) plus trastuzumab **Comparator**: Only equivalent sequential chemotherapy plus trastuzumab (paclitaxel plus trastuzumab, followed by fluorouracil, epirubicin, cyclophosphamide plus trastuzumab)	**Secondary:** Ki67 index	**Ki67** (analysis only non-pCR patients, n = 14, baseline to sugery)**:** Metformin group: decrease from 42.5% (Visual Analysis (VA))/34.5% (automated digital image analysis (ADIA) to 11% (VA)/11%(ADIA)), p = 0.025 (VA)/0.035 (ADIA) Control group: decrease from 36.0%(VA)/38.5%(ADIA) to 28.5%(VA)/25%(ADIA), p = 0.293 (VA)/0.080(ADIA) **Ki67** (analysis only non-pCR patients, change of category: high ≥ 20% versus low < 20%)**:** Metformin group: 57% (VA)/50% (ADIA) of high-Ki67 moved into the low-Ki67 Control group: 67%(VA)/80% (ADIA) of high-ki67 remained unchanged [*p* = 0.5210 (VA), p = 0.3582 (ADIA)]
METTEN Trial Cuyàs, 2019	79 patients included in the intention-to-treat (ITT) population of the METTEN study 100% not obese (exclusion criteria: BMI > 30) BMI ≥ 25 (overweight) Non-pCR: 12 (38.7%) pCR: 26 (54.2%) Post Non-pCR: 11 (35.5%) pCR: 20 (41.7%) Pre + Peri Non-pCR: 20 (64.5%) pCR: 28 (58.3%) 100% nondiabetic No data regarding: physical activity, and metabolic syndrome	**Phenotype**: HER2 **Receptors**: **ER/PgR + ** **Non-pCR:** 21 (67.7%) pCR: 22 (45.8%) **ER/PgR-:** **Non-pCR:** 10 (32.3%) pCR: 26 (54.2%) **SNP:** Metformin group: [A/A = 17 (51,5%); A/C, C/C = 16 (48,5%)]; Control group: [A/A = 20 (54,1%); A/C, C/C = 17 (45,9%)] **Stage**: Stage II—III **Treatment modality**: neoadjuvant	**Interventions**: Metformin 1.700 mg/day (850 mg BID) for 24 weeks concurrently with 12 cycles of weekly paclitaxel plus trastuzumab, followed by four cycles of 3-weekly fluorouracil, epirubicin, cyclophosphamide (FE75C) plus trastuzumab **Comparator**: Only equivalent sequential chemotherapy plus trastuzumab (paclitaxel plus trastuzumab, followed by fluorouracil, epirubicin, cyclophosphamide plus trastuzumab)	**Secondary:** C Allele of ataxia telangiectasia mutated (ATM) rs11212617 (if presence predict pCR)	**C Allele of ATM rs11212617:** **Relationship** **between rs11212617 genotype and the ability of the treatment groups to achieve pCR:** Adjusted OR genotype × arm: 20.53, 95%CI: 1.97 to 213.79, p = 0.011 **Relationship between rs11212617 C allele and pCR** Metformin group: adjusted OR A/A (ref) vs A/C, C/C = 28.88, 95%CI: 2.20 to 378.73, p = 0.010 Control group: adjusted OR A/A (ref) vs A/C, C/C: 0.53, 95%IC: 0.11 to 2.50), p = 0.425
METTEN Trial Cuyàs, 2019	68 patients included in the intention-to-treat (ITT) population of the METTEN study 100% not obese (exclusion criteria: BMI > 30) Overweight: Metformin group: 15 (45.5%) Control group: 19 (54.4%) Postmenopausal Metformin group: 13 (39.4%) Control group: 15 (42.9%) Premenopausal Metformin group: 20 (60.6%) Control group: 20 (57.1%) 100% nondiabetic No data regarding: physical activity, and metabolic syndrome	**Phenotype**: HER2 **Receptors**: **ER/PgR + ** Arm A with metformin: 18 (54.5%) Arm B control: 19 (54.3%) **ER/PgR-:** Metformin group: 15 (45.5%) Control group: 16 (45.7%) **Stage**: Stage II—III **Treatment modality**: neoadjuvant	**Interventions**: Metformin 1.700 mg/day (850 mg BID) for 24 weeks concurrently with 12 cycles of weekly paclitaxel plus trastuzumab, followed by four cycles of 3-weekly fluorouracil, epirubicin, cyclophosphamide (FE75C) plus trastuzumab **Comparator**: Only equivalent sequential chemotherapy plus trastuzumab (paclitaxel plus trastuzumab, followed by fluorouracil, epirubicin, cyclophosphamide plus trastuzumab)	**Secondary:** Serum metabolic profile [β-hydroxybutyrate (BHBA), α-ketoglutarate, cystathionine, taurine, betaine, choline, dimethylglycine, homocysteine, methionine, s-adenosyl methionine (SAM) and s-adenosyl homocysteine (SAH)]	**Serum metabolic profile (median fold-change post vs pre-treatment):** Differential impact between treatment arms: only BHBA (p = 0.038) and α-ketoglutarate (p = 0.029) reached statistical significance. Other metabolite levels were not significant **BHBA, α-ketoglutarate and homocysteine according to pCR status in metformin group** (fold-changes in serum levels post *vs* pre-treatment)**:** BHBA (pCR *vs* non-pCR): p = 0.134 α-ketoglutarate (pCR *vs* non-pCR): p = 0.082 Homocysteine (pCR *vs* non-pCR): p = 0.047 **Relationship between levels of serum homocysteine (post—pre-treatment) and the ability of the treatment groups to achieve pCR:** Adjusted OR follow-up homocysteine × group: 47.584, 95%CI: 1.60 to 1411.93, p = 0.026 **Relationship between homocysteine (higher levels, post—pre-treatment) and pCR** Metformin group: adjusted OR = 6.614, 95%CI: 0.822 to 53.189, p = 0.076 Control group: adjusted OR = 0.144, 95%CI: 0.010 to 2.077, p = 0.155
Pimentel, 2019	40 subjects were randomized (22 metformin group, 18 control group) No data regarding: obesity, overweight, menopause status, physical activity, and metabolic syndrome 100% nondiabetic	**Phenotype**: Luminal, HER2, Triple negative **Receptors**: 34 (85%) ER/PR +; 6 (15%) ER/PR-; 6 (15%)HER2 +; 34 (85%)HER2- **Stage**: 100% stage IV **Treatment line:** 27 (67,5%) 1 st line treatment; 7 (17,5%) 2nd line treatment; 6 (15%) 3rd or + line treatment **Treatment modality**: adjuvant and palliative	**Interventions**: Metformin 1.700 mg/day (850 mg BID) plus standard chemotherapy. 1st-4th line chemotherapy (prespecified anthracycline, taxane, vinorelbine, platinum or capecitabine, with allowance for HER2 targeted treatment) **Comparator**: Placebo plus standard chemotherapy	**Primary:** PFS **Secondary:** OS, Response rates, Toxicity, (QoL)	**PFS**: Metformin group: 5.4 months Control group: 6.3 months Adjusted HR = 1.43 (95%CI: 0.68 to 3.02), 2-sided p = 0.35, 1-sided p = 0.83 **OS**: Metformin group: 20.2 months Control group: 24.2 months Adjusted HR = 1.46 (95%CI: 0.68 to 3.13), 2-sided = 0.34, 1-sided p = 0.83 **Response Rates (partial response or stable disease):** Metformin group: 12 patients (54.5%) Control group: 7 patients (43.7%) OR = 1.77 (95%CI: 0.45 to 6.99), 2-sided p = 0.41, 1-sided p = 0.21 **Adverse events**: High grade (III or IV): Metformin group: 7 patients (31.8%) Control group: 10 patients (58.8%) Low grade (I or II): Metformin group: 15 patients (68.2%) Control group: 6 patients (35.3%) Gastrointestinal toxicity was the most common system organ class **QoL (Global Health Status—change from baseline to cycle 2)**: Metformin group: −0.6 (standardized effect size for change over time) Control group: 0.3 (standardized effect size for change over time) Standardized effect size for difference between arms, cycle 2 scores = 0.1
NCIC CTG MA.32 Goodwin, 2022	3649 patients; Both sexes were eligible for study; 3643 women [99.8%]) No data regarding: obesity, overweight, physical activity, and metabolic syndrome 2237 (61,3%) were postmenopausal. There are frequencies in the subpopulation according to HR status too 100% nondiabetic people	**Phenotype**: Luminal, HER2, Triple negative **Receptors**: **Status HR:** 2533 (69,4%) ER/PgR +; 1116 (30,6%) ER/PgR-; **Status HER2:** 3029 (83,0%)** HER2-;** 620 (17,0%) HER2 +; **Frequencies in the subpopulation according to HR status** **ER/PgR + patients:** 429 (16,9%) HER2 + **ER/PgR- patients:** 191 (17,1%) HER2 + **Stage**: Stages I – III **Treatment modality**: adjuvant	**Interventions**: Patients receive 850 mg of oral metformin twice daily (once daily in weeks 1–4). Treatment continues for up to 5 years in receptor positive (ER and/or PgR positive) subjects in the absence of disease progression or unacceptable toxicity. Standard adjuvant therapy (radiotherapy, chemotherapy, hormone, biologics) **Comparator**: Patients receive oral placebo twice daily (once daily in weeks 1–4). Treatment continues for up to 5 years in receptor positive (ER and/or PgR positive) subjects in the absence of disease progression or unacceptable toxicity Standard adjuvant therapy (radiotherapy, chemotherapy, hormone, biologics)	**Primary:** Invasive DFS **Secondary:** OS, distant relapse-free survival, breast cancer-free interval, new diabetes diagnosis, cardiovascular hospitalizations, quality of life, diet and physical activity	**Invasive DFS (incidence rates)** Group ER/PgR + Metformin group: 2.78 per 100 patient-years Control group: 2.74 per 100 patient-years (HR = 1.01, 95% CI: 0.84 to 1.21, p = 0.93 Metformin effect on invasive disease–free survival did not differ by rs11212617 SNV status (p for interaction = 0.97) Group ER/PgR − Metformin group: 3.58 per 100 patient-years Control group: 3.60 per 100 patient-years HR = 1.01, 95%CI: 0.79 to 1.30, p = 0.92 Metformin effect on invasive disease–free survival did not differ by rs11212617 SNV status (p for interaction = 0.31) Group ERBB2 + Metformin group: 1.93 events per 100 patient-years Control group: 3.05 events per 100 patient-years HR = 0.64, 95%CI: 0.43 to 0.95, p = 0.03) Group ERBB2 + by rs11212617 SNV status p for interaction = 0.05 *Subgroup C, A/C genotype*: Metformin group: 1.74 per 100 patient-years Control group: 3.48 per 100 patient-years HR = 0.51, 95%CI: 0.31 to 0.83, p = 0.007 *Subgroup AA genotype*: Metformin group: 2.5 per 100 patient-years Control group: 1.91 per 100 patient-years (HR = 1.32, 95%CI: 0.58 to 2.96, p = 0.51) Group ERBB2- Metformin group: 3.24 events per 100 patient-years Control group: 2.98 events per 100 patient-years HR = 1.09, 95%CI: 0.93 to 1.28, p = 0.29 **OS** Group ER/PgR + : Metformin group: 1.46 deaths per 100 patient-years Control group: 1.32 deaths per 100 patient-years HR = 1.10, 95%CI: 0.86 to 1.41, p = 0.47 Metformin effect on overall survival did not differ by rs11212617 SNV status (*p* for interaction = 0.46) Group ER/PgR-: Metformin group: 1.91 deaths per 100 patient-years Control group: 2.15 deaths per 100 patient-years HR = 0.89, 95%CI: 0.64 to 1.23, p = 0.46 Group ERBB2 + Metformin group: 0.78 deaths per 100 patient-years Control group: 1.43 deaths per 100 patient-years HR = 0.54, 95%CI: 0.30 to 0.98, p = 0.04 Group ERBB2 + by rs11212617 SNV status p for interaction = 0.02 *Subgroup C, A/C genotype*: Metformin group: 0.69 deaths per 100 patient-years Control group: 1.96 deaths per 100 patient-years (HR = 0.35, 95%CI: 0.17 to 0.73, p = 0.003) *Subgroup AA genotype*: Metformin group: 1.18 deaths per 100 patient-years Control group: 0.53 deaths per 100 patient-years (HR = 2.15, 95%CI: 0.56 to 8.36, p = 0.26 Group ERBB2- Metformin group: 1.76 deaths per 100 patient-years Control group: 1.59 deaths per 100 patient-years HR = 1.11, 95%CI, 0.90 to 1.36, p = 0.34 Metformin effect on overall survival did not differ by rs11212617 SNV status (p for interaction = 0.25) **Distant recurrence–free survival:** Group ER/PgR + : Metformin group: 1.99 per 100 patient-years Control group: 1.99 per 100 patient-years HR = 0.99, 95%CI: 0.80 to 1.23, p = 0.94; Group ER/PgR-: Metformin group: 2.35 per 100 patient-years Control group: 2.63 per 100 patient-years HR = 0.90, 95%CI: 0.67 to 1.20, p = 0.46) **Breast cancer–free interval:** Group ER/PgR + : Metformin group: 2.15 per 100 patient-years Control group: 2.18 per 100 patient-years HR = 0.98, 95%CI: 0.80 to 1.20; p = 0.87 Group ER/PgR-: Metformin group: 2.75 per 100 patient-years Control group: 3.14 per 100 patient-years HR = 0.88, 95%CI: 0.67 to 1.16, p = 0.35) **New diabetes diagnosis, cardiovascular hospitalizations, quality of life, diet and physical activity:** Not reported
NCIC CTG MA.32 Goodwin, 2021	Metabolic subpopulation: 2.915 patients (all available subjects with fasting blood at baseline and 6 months); Metabolic SNP subpopulation: 2.747 subjects (SNP information available) **Obesity:** Overall (≥ 30) n(%): 1000 (34.3%) **Overweight:** Overall (≥ 25 and < 30) n(%): 963 (33.03%) 100% nondiabetic people No data regarding: menopause status, physical activity, and metabolic syndrome	**Phenotype**: Luminal, HER2, Triple negative **Receptors**: **Hormone receptor status** ER/PgR-: 859 (29.5%) ER/PgR + : 2056 (70,5%) **HER2 status** HER2-: 2417 (82.9%) HER2 + : 498 (17.1%) **Stage**: Stages I – III **Treatment modality**: adjuvant	**Interventions**: Metformin 1.700 mg/day (850 mg BID) for 5 years, including a 4-week ramp-up of one caplet per day **Comparator**: Placebo	**Secondary:** Weight, BMI, glucose, insulin, HOMA, leptin, hsCRP (relative change from baseline to six months)	**Weight (Kg):** Metformin group: −2% Control group: + 1% *p* < 0.0001 Standardized ratio*: 0.97, 95%CI: 0.97 to 0.98, p < 0.0001 **BMI (kg/m**^**2**^**):** Metformin group: −2% Control group: + 1% *p* < 0.0001 Standardized ratio*: 0.97, 95%CI: 0.97 to 0.98, p < 0.0001 **Weight or BMI:** Interactions for baseline Weight or BMI: p = 0.76 Interactions for baseline insulin: p = 0.16 Interactions for SNP genotype: p = 0.69 **Glucose (mmol/L):** Metformin group: −2% Control group: + 1% *p* < 0.0001 Standardized ratio*: 0.98, 95%CI: 0.97 to 0.99, p < 0.0001 Interactions for baseline Weight or BMI: p = 0.94 Interactions for baseline insulin: p = 0.53 Interactions for SNP genotype: p = 0.21 **Insulin (pmol/L):** Metformin group: −11% Control group: + 6% Standardized ratio*: 0.85, 95%CI: 0.82 to 0.88, p < 0.0001 Interactions for baseline Weight or BMI: p = 0.53 Interactions for baseline insulin: p = 0.19 Interactions for SNP genotype: p = 0.84 **HOMA:** Metformin group: −10% Control group: + 11% *p* < 0.0001 Standardized ratio*: 0.83, 95%CI: 0.79 to 0.87, p < 0.0001 Interactions for baseline Weight or BMI: p = 0.86 Interactions for baseline insulin: p = 0.68 Interactions for SNP genotype: p = 0.59 **Leptin (ng/ml):** Metformin group: −10% Control group: + 12% p < 0.0001 Standardized ratio*: 0.80, 95%CI: 0.77 to 0.83, p < 0.0001 Interactions for baseline Weight or BMI: p = 0.02 Interactions for baseline insulin: p = 0.13 Interactions for SNP genotype: p = 0.65 **hsCRP (μg/L):** Metformin group: −9% Control group: + 10% *p* < 0.0001 Standardized ratio*: 0.84, 95%CI: 0.78 to 0.80, p < 0.0001 Interactions for baseline Weight or BMI: p = 0.93 Interactions for baseline insulin: p = 0.04 Interactions for SNP genotype: p = 0.74 There was no evidence that change in body size or blood variables differed in those receiving adjuvant endocrine therapy vs no (all interaction p ≥ 0.22) * relative levels in the two arms (metformin/control) at 6 months standardized to remove the baseline differences in the variable, age and BMI
NCIC CTG MA.32 Lohmann, 2017	The first 492 patients with paired blood samples 100% nondiabetic people No data regarding: obesity, overweight, menopause status, physical activity, and metabolic syndrome	**Phenotype**: Luminal, HER2, Triple negative **Receptors**: **Stage**: Stages I – III **Treatment modality**: adjuvant	**Interventions**: Metformin 1.700 mg/day (850 mg BID) for 5 years, including a 4-week ramp-up of one caplet per day **Comparator**: Placebo	**Secondary:** Vitamin B12, methylmalonic acid (MMA)* and homocysteine* (change from baseline to six months) *analyzed in patients with vitamin B12 levels below 222 pmol/L at baseline or six months	**Vitamin B12 (pmol/L)** (median change)**:** Metformin group: −51 [interquartile range (IQR)]: 6, −182 Control group: 0 (IQR: −61, + 65) p < 0.001 **MMA (lmol/L)** (median at six months)**:** Metformin group: 0.15 (IQR: 0.12, 0.2) Control group: 0.15 (IQR: 0.11, 0.2) p = 0.70 **Homocysteine (lmol/L)** (median at six months)**:** Metformin group: 9.5 (IQR: 8.5, 12.5) Control group: 9 (IQR: 8, 12) p = 0.57
NCIC CTG MA.32 Goodwin, 2021	CA 15–3 population (n = 2708) 100% nondiabetic people No data regarding: obesity, overweight, menopause status, physical activity, and metabolic syndrome	**Phenotype**: Luminal, HER2, Triple negative **Receptors**: **Hormone receptor status** ER/PgR-: 795 (29,4%) ER/PgR + : 1913 (70,6%) **HER2 status** HER2-: 2244 (82,9%) HER2 + : 464 (17,1%) **Stage**: Stages I – III **Treatment modality**: adjuvant	**Interventions**: Metformin 1.700 mg/day (850 mg BID) for 5 years, including a 4-week ramp-up of one caplet per day **Comparator**: Placebo	**Secondary:** CA 15–3 (change from baseline to six months)	**CA 15–3:** Metformin group: −7.7% Control group: −2.0% p < 0.001 Metformin/control standardized ratio (after correcting for differences in baseline CA 15–3, age, and BMI): 0.94 (95%CI: 0.92 to 0.96, p < 0.001) This outcome was not affected differentially depending on baseline stage (T-Stage clinical: p = 0.73; N-Stage clinical: p = 0.17; T-Stage pathologic: p = 0.12; N-Stage pathologic: p = 0.33), receptor status (ER/PgR: p = 0.35; HER2: p = 0.14), adjuvant treatment (hormones: p = 0.76; chemotherapy: p = 0.52; trastuzumab: p = 0.15), SNP status (p = 0.16), BMI (p = 0.87), or insulin level (p = 0.11)
NCIC CTG MA.32 Pimentel, 2021	312 patients 100% postmenopausal 100% nondiabetic women No data regarding: obesity, overweight, physical activity, and metabolic syndrome	**Phenotype**: Luminal, HER2, Triple negative **Receptors**: **Hormone receptor status** ER/PgR-: 312 (100,0%) ER/PgR + : 0 (0,0%) **HER2 status** HER2 + : 59 (18,9%) HER2-: 253 (81,1%) **Stage**: Stages I – III **Treatment modality**: adjuvant	**Interventions**: Metformin 1.700 mg/day (850 mg BID) for 5 years, including a 4-week ramp-up of one caplet per day **Comparator**: Placebo	**Secondary:** Estradiol, SHBG and bioavailable testosterone (change from baseline to six months)**;**	**Estradiol (pmol/L, median change):** Metformin group: −5.7 (Q1-Q3: −18.6, 0) Control group: 0 (Q1-Q3: −12.6, 14.7) p < 0.001 *Reduction in estradiol in metformin vs placebo:* -Adjusted for baseline BMI and BMI change: 25.7%, 95%CI: 13.2% to 36.4%) -Adjusted for change in insulin: 30.1%, 95%CI: 18.7% to 39.8% Reduction in estradiol associated with the metformin group was not affected by the C allele of the SNP rs11212617 **SHBG (nmol/L, median change)** Metformin group: −5.9 (Q1-Q3: −15.6, 3.0) Control group: −5.9 (Q1-Q3: −17.4, 1.5) P = 0.43 **Bioavailable testosterone (nmol/L, median change)** Metformin group: 0 (Q1-Q3: −0.01, 0.01) Control group: 0 (Q1-Q3: −0.01, 0.02) P = 0.24
EudraCT number 2008–004912-10 ISRCTN16493703 Bonanni, 2012	200 patients **Obesity:** 26 (13%) **Overweight:** 56 (28%) **Menopause status:** Premenopausal: 100 (50%) Perimenopausal: 12 (6%) Postmenopausal: 88 (44%) **DM:** 100% nondiabetic women **Metabolic syndrome:** Yes: 51 (25,50%) No: 146 (73%) Unknown: 3 (1,5%) No data regarding physical activity	**Phenotype**: Luminal, HER2, Triple negative **Receptors**: ER + pretreatment: 173 (86,5%) PgR + pretreatment: 142 (71%) HER2 + : 22 (11%) **Stage**: stage I to IIa **Treatment modality**: neoadjuvant	**Interventions**: days 1 to 3: 850-mg once daily to adapt to gastrointestinal symptoms; days 4 through 28: two 850-mg tablets after dinner (1700 mg daily) **Comparator**: days 1 to 3: placebo once daily to adapt to gastrointestinal symptoms; days 4 through 28: two tablets of placebo after dinner	**Primary:** Ki67 **Secondary:** Insulin, glucose, CRP, total cholesterol	Ki67 (Proportional change*) *Overall* Proportional Change: 4.0% (95%CI: −5.6 to 14.4, p interaction = 0.4) Group HOMA index (proportional change), per protocol HOMA index ≤ 2.8: 11.1% (95% CI, −0.6 to 24.2) HOMA index ˃ 2.8: −10.5% (95%CI: −26.1 to 8.4) *p* interaction = 0.045 Group BMI (proportional change), per protocol ≤ 27 kg/m^2^ (75th percentile): 8.2% (95%CI: −3.4 to 21.3) ˃ 27 kg/m^2^: −8.0% (95%IC: −23.2 to 10.3) *p* interaction = 0.143 Group Waist/hip girth ratio (proportional change) ≤ 0.88 (75th percentile): 9.9% (95%CI: −1.8 to 23.2) ˃ 0.88: −11.2% (95%CI: −26.3 to 6.9) *p* interaction = 0.058 Group Alcohol consumption (proportional change) No: 10.3% (95%CI: 0.8 to −22.6) Yes: −20.2% (95%CI: −35.6 to −0.9) *p* interaction = 0.005 Group C-reactive protein (proportional change) ≤ 2.69 mg/L (75th percentile): 9.4 (95%CI: −2.7 to 23.1) ˃ 2.69 mg/L: −11.3 (95%CI: −24.1 to 3.7) *p* interaction = 0.080 *Subgroup Luminal B tumors* Proportional Change: −1.75% (95% CI: −11.4 to 8.9, p = 0.7) *Subgroup Luminal B tumors by HOMA index (proportional change)* HOMA index ≤ 2.8: 5.01% (95%CI: −7.2 to 18.8, *p* = 0.4) HOMA index ˃ 2.8: −16.0% (95%CI, −30.9 to 2.1, p = 0.008) *p* interaction = 0.05 **Insulin (mU/L)** Proportional change: 2.3% (95%CI: −10.6 to 17.1; *p* = 0.739) Group BMI (proportional change) ≤ 27 kg/m^2^: 8.3% (95%CI: −4.6 to 22.9) ˃ 27 kg/m^2^: −13.8% (95%CI: −42.8 to 29.9) *p* interaction = 0.092 **Glucose (mg/dL)** Proportional change: −1.8% (95%CI: −4.7 to 1.3, *p* = 0.254) Group BMI (proportional change), ≤ 27 kg/m^2^: 0.04% (95%CI: −3.0 to 3.2) ˃ 27 kg/m^2^: −6.8 (95%CI: −14.0 to 1.0) p interaction = 0.015 **CRP (mg/L)** Proportional change: −19.9% (95%CI: −34.3 to −2.2; p = 0.029) Group BMI (proportional change), ≤ 27 kg/m^2^: −18.7 (95%CI: −35.1 to 1.9) ˃ 27 kg/m^2^: −23.6 (95%CI: −51.4 to 20.1) *p* interaction = 0.7 **Total cholesterol (mg/dL)** Proportional change: −5.3% (95%CI: −7.8 to −2.7, *p* < 0.001 Group BMI (proportional change), ≤ 27 kg/m^2^: −4.5 (95%CI: −7.2 to −1.6) ˃ 27 kg/m^2^: −7.9 (95%CI: −13.6 to −1.8) *p* interaction = 0.191 *[100 × exp (mean metformin post-treatment—mean placebo post-treatment)/(mean placebo post-treatment)]
EudraCT number 2008–004912-10 ISRCTN16493703 Cazzaniga, 2013	100 patients 100% nondiabetic women No data regarding: obesity, overweight, menopause status, physical activity, and metabolic syndrome	**Phenotype**: Luminal, HER2, Triple negative **Receptors**: Luminal A: 22 (22%) Luminal B: 55 (55%) HER2 + : 14 (14%) TNBC: 9 (9%) **Stage**: stage I to IIIa **Treatment modality**: neoadjuvant	**Interventions**: days 1 to 3: 850-mg once daily to adapt to gastrointestinal symptoms; days 4 through 28: two 850-mg tablets after dinner (1700 mg daily) **Comparator**: days 1 to 3: placebo once daily to adapt to gastrointestinal symptoms; days 4 through 28: two tablets of placebo after dinner	**Secondary:** Apoptotic cell nuclei by terminal deoxynucleotidyl transferase dUTP nick end labelling (TUNEL)	**Apoptotic cell nuclei by TUNEL** *Median difference surgery-biopsy levels:* Metformin group: + 4% (IQR: 2–12) Control group: + 2% (IQR: 0–8) *Median TUNEL levels at* *surgery:* Metformin group: 10% (IQR: 4–20) Control group: 8% (IQR: 3–15) p adjusted = 0.2 HOMA index *Median difference surgery-biopsy levels* HOMA index < 2.8 Metformin group: + 4% (IQR: 2–14) Control group: + 2% (IQR: 0–7) HOMA index ≥ 2.8: Metformin group: + 2% (IQR: 0–6) Control group: + 5% (IQR: 0–15) p interaction (HOMA and treatment) = 0.1 *Median TUNEL levels at* *surgery:* HOMA index < 2.8 Metformin group: 10% (IQR: 6–22) Control group: 6% (IQR: 3–12) p = 0.048 HOMA index ≥ 2.8: Metformin group: 6% (IQR: 4–10) Control group: 9% (IQR: 6–18) P = 0.3
EudraCT number 2008–004912-10 ISRCTN16493703 DeCensi, 2014	200 patients 100% nondiabetic women No data regarding: obesity, overweight, menopause status, physical activity, and metabolic syndrome	**Phenotype**: NI **Receptors**: 11% had HER2-positive tumors **Stage**: stage I to IIa **Treatment modality**: neoadjuvant	**Interventions**: days 1 to 3: 850-mg once daily to adapt to gastrointestinal symptoms; days 4 through 28: two 850-mg tablets after dinner (1700 mg daily) **Comparator**: days 1 to 3: placebo once daily to adapt to gastrointestinal symptoms; days 4 through 28: two tablets of placebo after dinner	**Secondary:** Effects of metformin according to BMI, glucose, insulin, HOMA index, C-peptide, CRP, IGFBP-1, IGFBP-3, IGF-I, free IGF-I, adiponectin and tumor subtype (difference surgery-baseline)	**Weight (kg)** Treatment effect (mean)*: −0.09 (95%CI: −0.85 to 0.67, p = 0.8) **BMI (kg/m**^**2**^**):** Treatment effect (mean)*: −0.05 (95%CI: −0.35 to 0.25, p = 0.7) **Glucose (mmol/L)** Treatment effect (mean)*: −0.64 (95%CI: −3.78 to 2.49, p = 0.7) **Insulin (mU/L)** Treatment effect (mean)*: −0.75 (95%CI: −3.36 to 1.85, p = 0.6) **HOMA index** Treatment effect (mean)*: −0.27 (95%CI −1.19 to 0.65, p = 0.6) **C-peptide (ng/mL)** Treatment effect (mean)*: −0.04 (95%CI: −0.25 to 0.17, p = 0.7) **hs-CRP (mg/L)** Treatment effect (mean)*: −0.47 (95%CI: −1.06 to 0.12, p = 0.1) Metformin decreased hs-CRP levels in women with BMI˃25 (p = 0.05) **IGFBP-1 (ng/mL)** Treatment effect (mean)*: −0.10 (95%CI: −0.98 to 0.78, p = 0.8) Metformin increased IGFBP-1 in women with BMI˃25 (p = 0.09) **IGFBP-3 (lg/mL)** Treatment effect (mean)*: 0.03 (95%CI: −0.10 to 0.15, p = 0.7) **IGF-I (ng/mL)** Treatment effect (mean)*: −3.1 (95%CI: −10.0 to 3.7, p = 0.4) **Free IGF-I** Treatment effect (mean)*: −0.006 (95%CI: −0.01 to 0.002, p = 0.1 **Adiponectin (ng/Ml)** Treatment effect (mean)*: −0.64 (95%CI: −1.17 to −0.12, p = 0.02) **Ki-67 (%)** Treatment effect (mean)*: 0.30 (95%CI: −1.93 to 2.53, p = 0.8) Group HOMA index (Ki67 change-difference between endpoint surgery and baseline biopsy) HOMA index ≤ 2.8: no association (p = 0.68) HOMA index > 2.8: greater decrease of Ki67 (p = 0.04) p interaction treatment*HOMA = 0.07 Group HER2 status (Ki67 change-difference between endpoint surgery and baseline biopsy) HER2 + : −4.6% HER2-: 0.9% p interaction = 0.076 Group hs-CRP p interaction = 0.02 Group IGFBP-3 p interaction = 0.04 Group IGFBP-1 p interaction = 0.02 *adjusted for the biomarker level at baseline, BMI and age
EudraCT number 2008–004912-10 ISRCTN16493703 DeCensi, 2015	142 patients **Obesity:** 10 (7%) **Overweight:** 46 (32,4%) **Menopause status:** Premenopausal: 76 (53,52%) Perimenopausal: 7 (4,93%) Postmenopausal: 59 (41,55%) **DM:** 100% nondiabetic women No data regarding: metabolic syndrome and physical activity	**Phenotype**: Luminal, HER2, Triple negative **Receptors**: ER + posttreatment: 126 (88,7%) PgR + posttreatment: 110 (77,5%) HER2 + posttreatment: 22 (15,5%) **Stage**: stage I to IIa **Treatment modality**: neoadjuvant	**Interventions**: days 1 to 3: 850-mg once daily to adapt to gastrointestinal symptoms; days 4 through 28: two 850-mg tablets after dinner (1700 mg daily) **Comparator**: days 1 to 3: placebo once daily to adapt to gastrointestinal symptoms; days 4 through 28: two tablets of placebo after dinner	**Coprimary:** Ki67 in adjacent ductal carcinoma in situ (DCIS), lobular carcinoma in situ (LCIS) and ductal hyperplasia	**Ki67:** Group Posttreatment in DCIS (Median) Metformin group: 12 (IQR: 8–20) Control group: 10 (IQR: 7–24) p = 0.9 *Subgroup DCIS grade 3* (Median) Metformin group: 33 (25–55) Control group: 40 (32–40) p = 0.2 p interaction = 0.2 *Subgroup DCIS grade 1/2* (Median) Metformin group: 10 (7–16) Control group: 10 (6–17) p = 0.9 *Subgroup DCIS HER2* + (Median) Metformin group: 22 (11–32) Control group: 35 (30–40) p = 0.06 p interaction = 0.04 *Subgroup DCIS HER2-* (Median) Metformin group: 16 (10–20) Control group: 17 (8–26) p = 0.7 *DCIS ER* + */HER2* + Metformin group: 12 (7–18) Control group: 32 (27–42) p = 0.004 p interaction = 0.001 *Subgroup DCIS ER* + */HER2-* Metformin group: 16 (10–20) Control group: 15 (8–22) p = 0.8 *Subgroup DCIS ER* + */PR* + */HER2* + Metformin group: 18 (12–18) Control group: 32 (24–44) p = 0.02 p interaction = 0.05 *Subgroup DCIS ER* + */PR* + */HER2-* Metformin group: 16 (10–20) Control group: 12 (8–20) p = 0.6 *Subgroup DCIS by HOMA* p interaction = 0.7 *Subgroup HER2* + */HOMA* < *2.8* Metformin group: 26.5 (18– 30.5) Control group: 32 (32–40) *Subgroup HER2* + */HOMA* ≥ *2.8* Metformin group: 7 (2–71) Control group: 38 (30–40) p = 0.3 Group Posttreatment in LCIS (Median) Metformin group: 15 (IQR: 5–15) Control group: 5 (IQR: 4–6) p = 0.1 Group Posttreatment in ductal hyperplasia (Median) Metformin group: 3 (IQR: 1–4) Control group: 3 (IQR: 1–4) p = 0.5 *Subgroup Waist/hip girth ratio* There was a lower proliferation fraction in women with abdominal adiposity (waist/hip girth ratio > 0.85) in the metformin group (p interaction = 0.05)
**Studies with results published only in the form of abstracts**					
Arce-Salinas, 2016	48 women were included, 28 were assigned to the experimental group and 20 in the control group Obesity rate was 46.2% (metformin group) and 47.1% (placebo group) for each treatment arm 100% nondiabetic Metabolic syndrome was more prevalent in experimental arm 61.5% (metformin group) vs 52.9% (placebo group) No data regarding: menopause status and physical activity	**Phenotype**: Luminal = 48 (100%) **Receptors**: ER/PR + HER2- (100%) **Stage**: Stage III: 48 (100%) **Treatment Modality**: neoadjuvant	**Interventions**: Metformin 850 mg PO q12H with NeoCT **Comparator**: Placebo with NeoCT	**Primary:** pCR (assessed after 24 weeks of treatment) **Secondary:** Safety analysis, assess clinical response and to correlate serum levels of insulin, protein C and HbA1 with clinical and pathologic response	**pCR:** Metformin group: 26.9% Control group: 5.9% *p* = 0.22 **Safety analysis**: No significant toxicity was associated with metformin No information for the others secondary outcomes
Azazy, 2020	60 participants (30 in each arm) No data regarding: obesity, overweight, menopause status, physical activity, and metabolic syndrome 100% nondiabetic	**Phenotype**: NI **Receptors**: NI **Stage**: Stage II—III **Treatment Modality**: neoadjuvant	**Interventions**: Metformin 1.700 mg/day (850 mg bid) starting with neoadjuvant chemotherapy (fluorouracil, epirubicin, cyclophosphamide (FEC) then docetaxel ± trastuzumab) **Comparator**: Neoadjuvant chemotherapy alone ((FEC) then docetaxel ± trastuzumab)	**Primary:** pCR and pathological partial response (pPR) **Secondary:** Toxicity	**pCR and pPR:** There was a significant difference between metformin and control groups (p = 0.03). pCR was higher in metformin group, but did not reach significance due to the small sample size (p = 0.09) There was more pronounced response in the Luminal B subtype (p = 0.03) and in grade II tumours (p = 0.03) **Toxicity:** There was no significant difference in overall toxicity or ≥ grade 3 adverse events between both groups
Sadighi, 2016	45 participants (25 metformin group; 20 control group) No data regarding: obesity, overweight, menopause status, physical activity, and metabolic syndrome 100% nondiabetic	**Phenotype**: NI **Receptors**: NI **Stage**: NI **Treatment Modality**: neoadjuvant	**Interventions**: Metformin (1500 mg/day) was prescribed to intervention group from pathology report to the night before surgery **Comparator**: Patients with the same inclusion criteria who did not receive any drug	**Primary:** Ki67 **Secondary:** Toxicity	**Ki67:** Metformin group: Decrease of median from 35.14 to 29.6 Control group: Increase of median from 24.5 to 30.6 Both results were statistically significant *HOMA* There was a correlation between metabolic factor of HOMA score and changes in Ki67 **Toxicity:** Although mild gastrointestinal symptoms were seen in 30% of cases, generally patients tolerated metformin very well
IBIS 3 Trial (**Feasibility study**)^a^ (Oke, 2017)	Planned number of subjects to be included in the whole clinical trial: 300 patients; **Total final enrolment**: 89 No data regarding: obesity, overweight, physical activity, and metabolic syndrome 100% postmenopausal 100% nondiabetic women	**Phenotype**: NI **Receptors**: ER + **Stage**: NI **Treatment Modality**: prevention of recurrence after end of cancer treatment	**Interventions**: Aromatase inhibitor (anastrozole, letrozole or examestane) and/or metformin (1700 mg/day) and/or zoledronic acid 2 × 2x2 factorial design **Comparator**: Standard care (No investigational medicinal product assigned in this arm)	**Primary**: To determine acceptability and feasibility of recruitment, recruitment rate and number of sites required for main trial investigating prevention of late recurrence with an aromatase inhibitor and/or metformin and/or zoledronic acid or standard care (no treatment) **Secondary**: To determine reasons for non-participation/drop-outs and address these for main trial; To evaluate treatment adherence and reasons for stopping; To determine reasons for non-adherence and address for main trial; To assess the use of recruiting through GP surgeries local to sites via the PCRN/LCRN; To investigate feasibility of the use of email for data collection of PROs from patients; To assess acceptability of investigations for main trial	Overall, only 5% GPs (?) agreed to participate and only 23 of 800 (3%) subsequently responded to the survey The main reasons identified for nonparticipation were lack of time/resources to carry out database search (61%) and/or review medical records to confirm eligibility (48%), request coming at a busy time (9%) e.g. Calendar or financial year-end, and insufficient funding (26%)

ADIA: Automated digital image analysis; ATM:Ataxia telangiectasia mutated; BCR: Breast Conservative Rate; BDI: Beck Depression Inventory; BHBA: β-hydroxybutyrate; BMI: Body Mass Index; CA15-3: Cancer Antigen 15–3; CBR: Clinical Benefit Rate; CBS: Conservative breast surgery; cCR: Complete Clinical Response; CI: confidence interval; cPR: Partial Clinical Response; CRP: C-reactive protein; CRR: Clinical Response Rate; cSD: Clinical Stable Disease; CT: Chemotherapy treatment; CTC: Circulating tumor cells; DCIS: Ductal carcinoma in situ; DLT: Dose limiting toxicity; DFS: Disease-free survival; eGFR: Glomerular Filtration Rate; ER/PgR: Estrogen receptor and/or progesterone receptor; ER: Estrogen receptor; ET: Endometrial Thickness; FBG: Fasting blood glucose; HbA1c: Glycated hemoglobin; HDL: High-density lipoprotein; HOMA-IR: Homeostasis Model Assessment of Insulin Resistance; HR: Hazard Ratio; hs-CRP: High sensitivity CRP; HT: Hormonal treatment; IGF: Insulin-like Growth Factor; IGFBP: Insulin-like Growth Factor Binding Protein; IQR: Interquartile range; IL-6: Interleukin 6; IGF-1R: Insulin-like growth factor-1 receptor; LCIS: Lobular carcinoma in situ; LDL: Low-density lipoprotein; MMA: Methylmalonic acid; MRM: Modified radical mastectomy; OR: Odds Ratio; ORR: Objective Response Rate; OS: Overall survival; pCR: Pathological complete response; pPR: Pathological partial response; PFS: Progression-free survival

^a^IBIS 3 Trial is a feasibility study whose results were published in a conference abstract (Oke, 2017) and European Union Clinical Trials Register

### Breast cancer and other tumors

Of the 40 clinical trials included, 35 focused exclusively on patients with breast cancer, while five [50–52, 59, 62] included participants with multiple types of cancer. Two of these trials [59, 62] were available only in the form of a conference abstract and a trial registry without results or detailed research protocols. Although the other three studies [50–52] published their results, they did not report them separately by type of cancer.

### General characteristics of participants from the included studies

In total, the 25 studies with published results focused on individuals with breast cancer and included 5,623 participants. The participant counts ranged from 40 to 3,649 (median 73 and interquartile range [IQR] 51 to 121). While most studies involved female participants, four studies [28, 52, 59, 73] included both men and women with breast cancer in their samples, with the majority comprised by women.

The three studies [50–52], which included individuals with multiple types of cancer and had some form of published results, did not offer separate data for the 176 patients with breast cancer.

The total population enrolled in the four studies [70–73] that were terminated without publishing their results was 244 patients. Finally, the total number of participants planned to enroll according to the research protocols of ongoing studies [59–69] was 2,090.

### The distribution of comorbidities among participants in the included studies

The level of reporting on participants’ comorbidities in the included studies was heterogeneous. The most frequently reported comorbidities included obesity, overweight status, hypercholesterolemia, hypertension, diabetes, prediabetes, and metabolic syndrome.

#### Obesity/overweight

Liubota’s trial [43] was the only study in which all participants were obese (BMI > 30). In three other studies [41, 48, 51], participants were either overweight or obese (BMI > 25). In contrast, the METTEN Trial [24] was the only study that excluded any potential participants with a BMI above 30. For the remaining studies with published results [22, 45, 47, 54, 58], the prevalence of overweight and/or obesity among participants ranged from 13 to 43%. Fifteen studies [20, 21, 28, 39, 40, 42, 44, 46, 47, 50, 52, 55–58] did not report any data on that characteristic of their samples.

#### Diabetes and prediabetes

Of the 25 studies with published results, 18 [20–22, 24, 28, 35, 40, 42, 44, 45, 47, 50, 53–58] exclusively recruited participants without diabetes. Five [39, 43, 46, 48, 52] of these studies did not provide information about the diabetes status of their samples. Only two studies did not exclude participants with diabetes or prediabetes. In one study [41], 1% of participants had diabetes, while in the other study [51], 17% of participants had prediabetes.

#### Metabolic syndrome

Among the studies reviewed, only three [35, 43, 54] provided data regarding the frequency of metabolic syndrome among their participants. Notably, one [43] of these studies exclusively included participants with metabolic syndrome. In the remaining two [35, 54] studies, the prevalence of metabolic syndrome among participants ranged from 26 to 58%. It is worth noting that 22 [20–22, 24, 28, 39–42, 44–48, 50–53, 55–58] of the studies did not include any data related to this particular characteristic of their samples.

#### Menopause status

Among the 25 studies with published results, one study [47] exclusively involved a postmenopausal population. Additionally, five studies [21, 42, 46, 48, 57] specifically focused on postmenopausal women. Ten studies [22, 24, 28, 35, 39, 41, 43–45, 49] examined a mixed population, encompassing pre, peri-, and postmenopausal women. Moreover, nine studies [40, 50–56, 58] did not provide information about the menopausal status of the participants.

### Characteristics of breast cancer

#### Histological type

Only five studies [35, 39, 41, 43, 49] provided information on the frequencies of histological types of breast cancer among their participants. In these studies, the frequencies of ductal, lobular, mixed, or other histological types ranged from 3 to 90%, 4% to 10%, 3% to 8%, and 3% to 3.5%, respectively.

#### Breast cancer phenotype

**Fig. 2:**
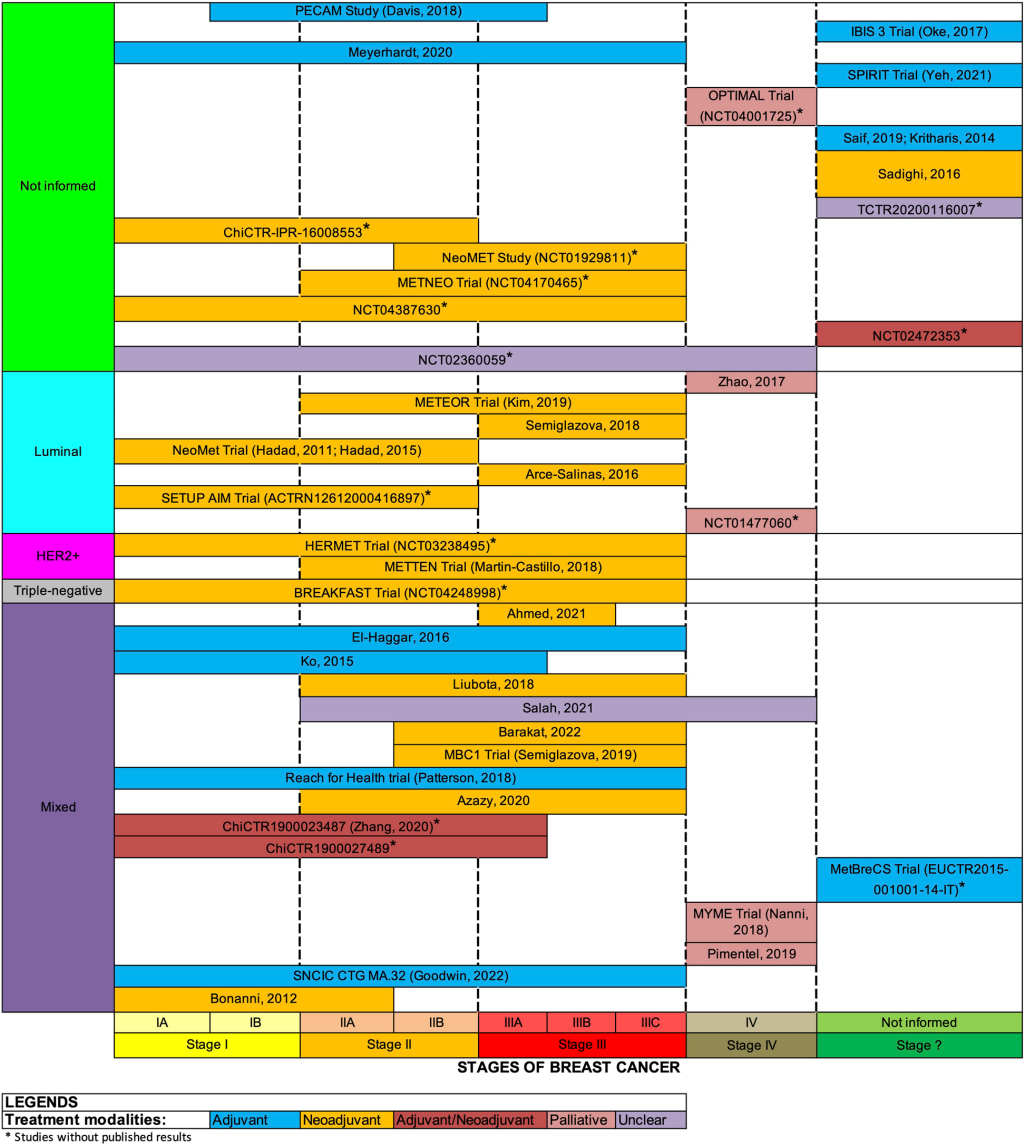
Overview of the Studies Included in the Scoping Review According to Phenotypes, Stages of Breast Cancer, Treatment Modalities, and Status of Publication Results

Figure 2 presents an overview of the studies included in the scoping review according to four aspects: phenotypes, stages of breast cancer, treatment modalities, and status of publication results.

The reporting of breast cancer phenotypes varied considerably among the 25 studies with published results. Six studies [21, 42, 50–52, 56] did not mention the phenotypic classification of breast cancer. Five studies [20, 46, 47, 54, 57] explicitly included only participants with the luminal phenotype. A single study [24], consisting of three substudies [25–27], included only HER2 + breast cancer patients. Thirteen studies [22, 28, 35, 39–41, 43–45, 48, 53, 55, 58] included participants with mixed phenotypes. In this group, the frequencies of luminal, HER2 +, and triple-negative phenotypes ranged from 62 to 84%, 6% to 40%, and 9% to 12%, respectively. Six mixed studies [22, 28, 35, 44, 53, 58] only provided information about the frequency of the hormonal and HER2 receptors of breast cancers in isolation, without explicitly determining the frequency of each phenotype.

**Fig. 3:**
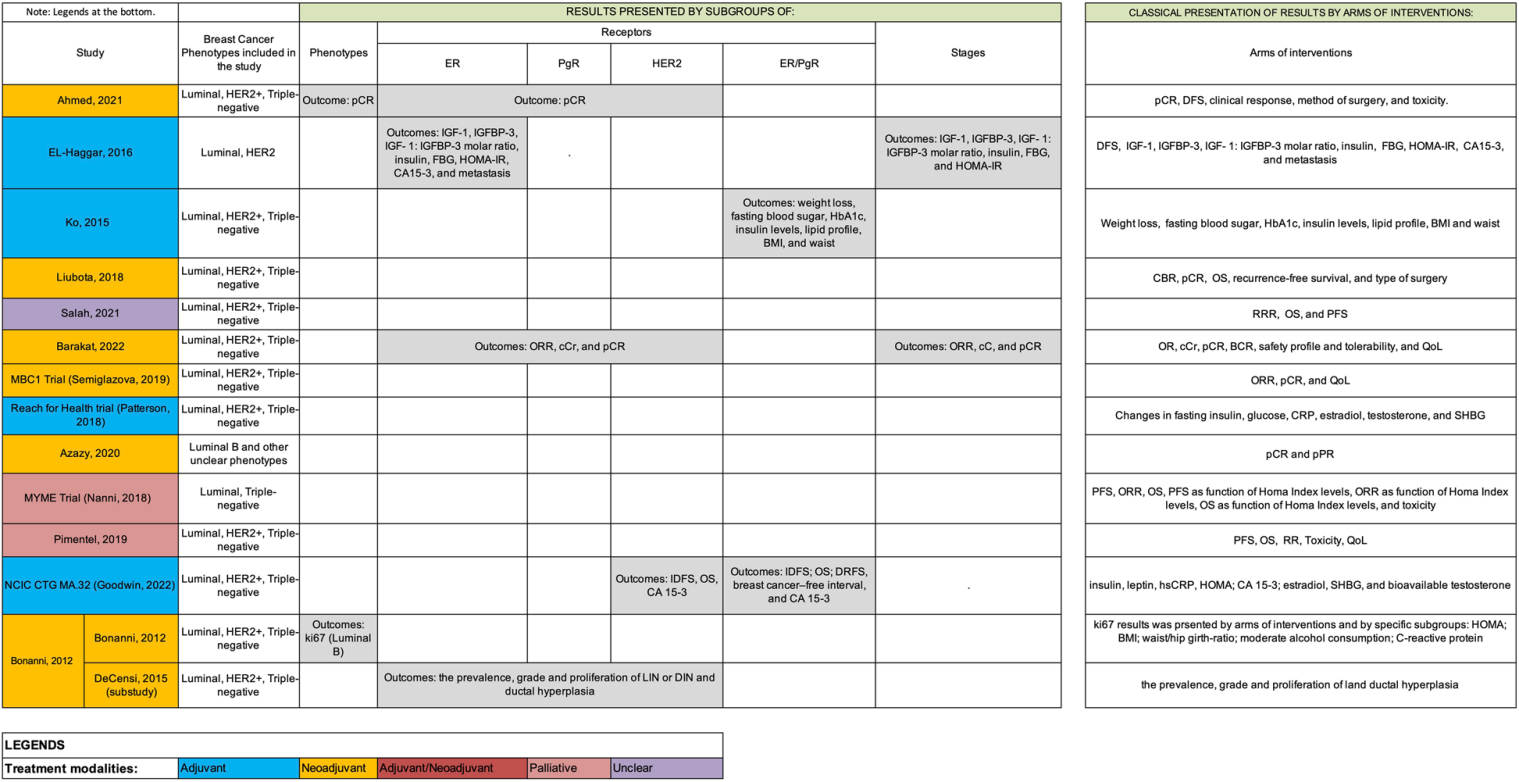
Clinical and Nonclinical Outcomes in Studies Involving Mixed Breast Cancer Phenotypes BCR = breast conservation rate; CBR = clinical benefit rate; cCr = clinical complete response; CRP = C-reactive protein; DFS = disease free survival; DRFS = distant recurrence–free survival; DIN = ductal intraepithelial neoplasia; ER = estrogen receptor; FBG = fasting blood glucose; HbA1c = glycated hemoglobin; HER2 = human epidermal growth factor receptor 2; HOMA-IR = homeostatic model assessment for insulin resistance; IGF-1 = insulin-like growth factor 1; IGFBP-3 = insulin-like growth factor binding protein 3; IDFS = invasive disease-free survival; LIN = lobular intraepithelial neoplasia; ORR = objective response rate; pPR = partial pathological response; pCR = pathological complete response; PgR = progesterone receptor; RRR = radiological response rate; RR = response rates; SHBG = sex hormone-binding globulin

Figure 3 illustrates the clinical and nonclinical outcomes of the 13 studies involving mixed breast cancer phenotypes. While all of these studies traditionally presented results according to intervention arms, six studies [28, 35, 39–41, 45] segmented some outcome results by subgroups based on phenotypes, receptors, or stages of breast cancer. Ahmed’s study [39] provided pathological complete response (pCR) results according to phenotypes, and Bonanni’s study [35] reported ki67 results for the luminal phenotype. Six studies [28, 38–41, 45] reported relevant clinical outcomes, such as pCR, objective response rate (ORR), invasive disease–free survival (IDFS), and overall survival (OS), according to hormone and HER2 receptor subgroups. Finally, two studies [40, 45] reported results for clinical and nonclinical outcomes based on subgroups of breast cancer stages.

#### Stages of breast cancer

The stages of breast cancer investigated were also heterogeneous among the 25 studies with some published results (Fig. 2). Two studies [20, 35] included participants with stages I and II disease. Six studies [28, 40–42, 48, 50] included participants with breast cancer stages ranging from I to III. Six other trials [24, 43, 45, 55, 57, 58] included patients with stages II to III disease. Three studies [39, 47, 54] included only stage III patients. One study [44] involved a stage interval from IIa to IV. Three studies [22, 46, 53] were restricted to participants with stage IV breast cancer. Finally, four studies [21, 51, 52, 56] did not provide any information regarding the stage of breast cancer among their participants.

### Interventions and Comparators

The RCTs included in this review examined metformin as a standalone intervention or in combination with various oncological treatments, employing a variety of comparators. The dosages of metformin investigated in these trials ranged from 850 to 2550 mg per day.

Of the 25 studies with published results, four trials [28, 35, 41, 42] administered metformin as a single intervention and compared it with placebo. In Sadighi’s study [56] and the NeoMet Trial [49], metformin was given to participants in the experimental group, while the participants in the control group received no medication.

Furthermore, among the same set of studies, three trials [48, 50, 51] combined metformin with behavioral interventions such as weight loss or exercise training, aiming to prevent recurrence after cancer treatment. The SPIRIT trial [51] had three intervention arms: metformin alone, coach-directed weight loss, and self-directed weight loss. The Reach for Health trial [48], a 2 by 2 factorial randomized trial, included groups receiving metformin alone and weight loss with metformin interventions and compared them with placebo groups and weight loss with a placebo. Meyerhardt’s study [50] compared metformin with training exercises, exercise training alone, and metformin alone, and the results were compared with those of an educational information group without treatment.

Of the 25 studies with published results, 16 incorporated metformin in combination with standard care systemic therapy, which could involve chemotherapy, hormone therapy, or a combination of both. Specifically, ten studies [22, 24, 39, 44, 45, 52–55, 58] employed chemotherapy, four [21, 46, 47, 57] used hormone therapy, and two [40, 43] utilized both chemotherapy and hormone therapy.

Among the studies examining chemotherapy, eight [22, 24, 39, 44, 45, 52, 55, 58] combined metformin with various chemotherapy regimens and compared them to chemotherapy alone. Two studies [53, 54] administered metformin in conjunction with chemotherapy and compared it with the same chemotherapy plus a placebo.

Among the studies evaluating hormone therapy, two [21, 47] combined metformin with hormone therapy and compared it with hormone therapy without metformin. For Semiglazova’s three-arm trial comparing metformin plus toremifene vs. toremifene alone and toremifene plus melatonin, we considered only the arms with metformin and its control for our review. Two studies [46, 57] combined metformin with hormone therapy, specifically an aromatase inhibitor, and compared it with the same hormone therapy plus a placebo.

Finally, among two studies [40, 43] that tested both chemotherapy and hormone therapy in conjunction with metformin, the control groups underwent the same interventions without metformin. In El-Haggar’s study [40], metformin was tested in combination with chemotherapy and hormone therapy as adjuvant systemic therapy. In Liubota’s study [43], both systemic therapies were applied as neoadjuvant treatments, with some patients receiving four cycles of anthracycline-based chemotherapy on three-week schedules and postmenopausal patients with luminal breast cancer receiving hormone therapy (letrozole 2.5 mg per day) for 16 weeks.

### Treatment Modalities

Metformin was examined in various treatment modalities across the studies included in this review (Fig. 2). Of the 25 studies with published results, twelve [24, 35, 39, 43, 45, 47, 49, 54–58] included metformin as a neoadjuvant treatment for breast cancer, while nine [21, 28, 40–42, 48, 50–52] incorporated it as an adjuvant treatment. Additionally, three studies [22, 46, 53] utilized metformin for palliative care. Finally, in one specific study [44], the treatment modality for investigating metformin in combination with standard chemotherapy was unclear.

Of the three studies that incorporated metformin into palliative breast cancer treatment, only the MYME Trial [22] and its substudy, TransMYME [23], explicitly stated the use of 100% first-line chemotherapy. In the other two studies involving participants with stage IV breast cancer receiving palliative oncological treatment, one [46] combined metformin with first-, second-, and third-line or subsequent treatments in 22 (36.7%), 35 (58.3%), and 3 (5.0%) of their patients, respectively. Another study [53] combined metformin with first-, second-, and third-line or subsequent treatments in 27 (67.5%), 7 (17.5%), and 6 (15%) of the participants, respectively.

### Outcome measures and main findings

Overall, among the 25 included studies with published results, the most frequently reported outcomes were toxicity, pathological complete response (pCR), objective response rate (ORR), clinical benefit rate (CBR), and insulin levels. Table 2 provides a list of clinical, nonclinical, and miscellaneous outcome measures evaluated by all 40 studies included in this review. Similarly, **Supplement 5** includes three spreadsheets where readers may find a list of the outcome measures examined by each study according to the breast cancer phenotype, stage, and treatment modality of their samples. The first tab compiles the clinical outcomes mapped in the trials included in the scoping review, with survival outcomes presented initially, as they hold the utmost significance for patients with breast cancer. The second tab organizes nonclinical outcomes assessed in the trials by categories. The final tab consolidates other nonclinical outcomes that do not fit into any specific category.

**Table 2:** Clinical, non-clinical, and miscellaneous outcomes evaluated by the studies included in the Scoping Review

Outcomes	Studies with at least some published results	Studies without published results
**Clinical Outcomes**		
**Survival**		
Overall survival (OS)	MYME Trial (Nanni, 2018); Zhao, 2017; Liubota, 2018; Salah, 2021; Pimentel, 2019; NCIC CTG MA.32 (Goodwin, 2022)	NCT01477060; BREAKFAST Trial (NCT04248998); ChiCTR1900023487 (Zhang, 2020)
Disease-free survival (DFS)	METTEN Trial (Martin-Castillo, 2018); Ahmed, 2021; El-Haggar, 2016; Liubota, 2018; NCIC CTG MA.32 (Goodwin, 2022)	ChiCTR1900023487 (Zhang, 2020); ChiCTR1900027489
Progression-free survival (PFS)	MYME Trial (Nanni, 2018); Zhao, 2017; Salah, 2021; Pimentel, 2019	Saif, 2019/Kritharis, 2014; NCT01477060; ChiCTR1900027489
Relapse-free survival (RFS)	NCIC CTG MA.32 (Goodwin, 2022)	-
Distant metastasis-free survival (DMFS)	-	BREAKFAST Trial (NCT04248998)
Mortality	-	OPTIMAL Trial (NCT04001725)
**Response Rates**		
Objective Response Rate (ORR)	Zhao, 2017; METEOR Trial (Kim, 2019); Semiglazova, 2018; Ahmed, 2021; Liubota, 2018; MBC1 Trial (Semiglazova, 2019); MYME Trial (Nanni, 2018)	NeoMET Study (NCT01929811); NCT04387630; Arce-Salinas, 2016; NCT01477060; BREAKFAST Trial (NCT04248998); Salah, 2021; ChiCTR1900023487 (Zhang, 2020)
Clinical Benefit Rate (CBR)	Zhao, 2017; Ahmed, 2021; Liubota, 2018; Salah, 2021; Pimentel, 2019	NeoMET Study (NCT01929811); NCT04387630; Arce-Salinas, 2016; BREAKFAST Trial (NCT04248998);
Pathological Complete Response (pCR)	Arce-Salinas, 2016; Semiglazova, 2018; METTEN Trial (Martin-Castillo, 2018); Ahmed, 2021; Liubota, 2018; Barakat, 2022; MBC1 Trial (Semiglazova, 2019); Azazy, 2020	ChiCTR-IPR-16008553; NeoMET Study (NCT01929811); METNEO Trial (NCT04170465); METEOR Trial (Kim, 2019); HERMET Trial (NCT03238495); BREAKFAST Trial (NCT04248998)
**Breast-related outcomes**		
Breast conservation rate (BCR)	METEOR Trial (Kim, 2019); METTEN Trial (Martin-Castillo, 2018); Ahmed, 2021; Liubota, 2018; Barakat, 2022	NeoMET Study (NCT01929811)
Breast density change	METEOR Trial (Kim, 2019); NCIC CTG MA.32 (Goodwin, 2022)	-
Breast cancer–free interval	NCIC CTG MA.32 (Goodwin, 2022)	-
Breast cancer events	-	ChiCTR-IPR-16008553
Clinical resection of lump	-	ChiCTR-IPR-16008553
Prevalence, grade and proliferation of lobular or ductal intraepithelial neoplasia (LIN or DIN) and ductal hyperplasia	Bonanni, 2012/DeCensi, 2015	-
ECOG performance status	-	OPTIMAL Trial (NCT04001725)
**Other Clinical outcomes**		
Metastasis	El-Haggar, 2016	-
Quality of Life (QoL)	Semiglazova, 2018; Barakat, 2022; MBC1 Trial (Semiglazova, 2019); Pimentel, 2019; NCIC CTG MA.32 (Goodwin, 2022)	OPTIMAL Trial (NCT04001725); NCT02472353; ChiCTR1900023487 (Zhang, 2020)
Toxicity	Saif, 2019/Kritharis, 2014; Azazy, 2020; Sadighi, 2016; MYME Trial (Nanni, 2018); Zhao, 2017; METEOR Trial (Kim, 2019); Semiglazova, 2018; METTEN Trial (Martin-Castillo, 2018); Ahmed, 2021; Barakat, 2022; MBC1 Trial (Semiglazova, 2019); Pimentel, 2019	NeoMET Study (NCT01929811); METNEO Trial (NCT04170465); NCT02472353; NCT01477060; BREAKFAST Trial (NCT04248998); ChiCTR1900023487 (Zhang, 2020); ChiCTR1900027489
**Non-clinical Outcomes**		
**Comorbidities**		
Blood pressure levels	Ko, 2015	-
New diagnosis of Diabetes mellitus	NCIC CTG MA.32 (Goodwin, 2022)	OPTIMAL Trial (NCT04001725)
New diagnosis of Metabolic syndrome	-	ChiCTR-IPR-16008553
**Nutrition**		
Diet	NCIC CTG MA.32 (Goodwin, 2022)	-
Weight loss	Ko, 2015	-
**Anthropometric Measurements**		
Body Mass Index (BMI)	PECAM Study (Davis, 2018); Meyerhardt, 2020/Brown, 2020; SPIRIT Trial (Yeh, 2021); NCIC CTG MA.32 (Goodwin, 2022); Bonanni, 2012	-
Weight	PECAM Study (Davis, 2018); Meyerhardt, 2020/Brown, 2020; SPIRIT Trial (Yeh, 2021); Ko, 2015	-
Height	Ko, 2015	-
Abdominal circumference	PECAM Study (Davis, 2018)	-
Waist circunference	Ko, 2015	-
Waist to Hip Ratio	Meyerhardt, 2020/Brown, 2020	-
**Fertility**		
Endometrial thickness	PECAM Study (Davis, 2018)	-
Menstruation recovery rate	-	ChiCTR1900023487 (Zhang, 2020); ChiCTR1900027489
5-year pregnancy	-	ChiCTR1900023487 (Zhang, 2020); ChiCTR1900027489
**Sex Hormones**		
Anti-Müllerian Hormone (AMH)	-	ChiCTR1900023487 (Zhang, 2020); ChiCTR1900027489
Inhibin B	-	ChiCTR1900023487 (Zhang, 2020); ChiCTR1900027489
Estradiol	NCIC CTG MA.32 (Goodwin, 2022)	ChiCTR1900023487 (Zhang, 2020); ChiCTR1900027489
Progesterone	-	ChiCTR1900023487 (Zhang, 2020); ChiCTR1900027489
Prolactin (PRL)	-	ChiCTR1900023487 (Zhang, 2020); ChiCTR1900027489
Follicle Stimulating Hormone (FSH)	-	ChiCTR1900023487 (Zhang, 2020); ChiCTR1900027489
Luteinizing Hormone (LH)	Reach for Health trial (Patterson, 2018)	ChiCTR1900023487 (Zhang, 2020); ChiCTR1900027489
Testosterone	Reach for Health trial (Patterson, 2018); NCIC CTG MA.32 (Goodwin, 2022)	ChiCTR1900023487 (Zhang, 2020); ChiCTR1900027489
Sex Hormone-Binding Globulin (SHBG)	Reach for Health trial (Patterson, 2018); NCIC CTG MA.32 (Goodwin, 2022)	ChiCTR1900023487 (Zhang, 2020); ChiCTR1900027489
**Genetic Tests**		
Genomic analysis	-	NCT02472353
mTOR pathway gene expression	PECAM Study (Davis, 2018)	-
C Allele of ATM rs11212617	METTEN Trial (Martin-Castillo, 2018)	-
Genes associated with doxorubicin-induced cardiotoxicity at baseline	-	TCTR20200116007
**Bloodtests**		
Blood routine	-	ChiCTR1900027489
Urine routine	-	ChiCTR1900027489
Fasting Serum Urate (SU)	SPIRIT Trial (Yeh, 2021)	-
Vitamin B12	NCIC CTG MA.32 (Lohmann, 2017)	-
**Liver function**		
Aspartate aminotransferase (AST)	-	ChiCTR1900027489
Alanine aminotransferase (ALT)	-	ChiCTR1900027489
**Kidney function**		
Estimated glomerular filtration rate (eGFR)	SPIRIT Trial (Yeh, 2021)	ChiCTR1900027489
**Blood-based energetic biomarkers**		
Glucose	PECAM Study (Davis, 2018); Meyerhardt, 2020/Brown, 2020; Ko, 2015; Reach for Health trial (Patterson, 2018)	OPTIMAL Trial) (NCT04001725); ChiCTR-IPR-16008553;
Insulin	PECAM Study (Davis, 2018); Meyerhardt 2020/Brown, 2020; SPIRIT Trial (Yeh, 2021); METTEN Trial (Martin-Castillo, 2018); El-Haggar, 2016; Ko, 2015; Reach for Health trial (Patterson, 2018); NCIC CTG MA.32 (Goodwin, 2022)	ChiCTR-IPR-16008553
Homeostatic Model Assessment for Insulin Resistance (HOMA-IR)	PECAM Study (Davis, 2018); Sadighi, 2016; MYME Trial (Nanni, 2018); El-Haggar, 2016; NCIC CTG MA.32 (Goodwin, 2022); Bonanni, 2012	-
Hemoglobin A1C (HbA1c)	PECAM Study (Davis, 2018); Sadighi, 2016; MYME Trial (Nanni, 2018); El-Haggar, 2016; NCIC CTG MA.32 (Goodwin, 2022); Bonanni, 2012	-
IGF1R (Insulin Like Growth Factor 1 Receptor)	MYME Trial (Nanni, 2018); Bonanni, 2012	-
IGF-1 (insulin-like growth factor 1)	Meyerhardt, 2020/Brown, 2020; SPIRIT Trial (Yeh, 2021); El-Haggar, 2016; Bonanni, 2012	-
IGFBP-1 (Insulin-like growth factor-binding protein 1)	Bonanni, 2012	-
IGFBP-3 (Insulin-like growth factor binding protein 3)	El-Haggar, 2016; Bonanni, 2012	-
IGF1:IGFBP3 molar ratio	SPIRIT Trial (Yeh, 2021); El-Haggar, 2016	-
**Cardiac-related outcomes**		
LVEF (Left Ventricle Ejection Fraction)	-	NCT02472353
Troponin-I level	-	TCTR20200116007; NCT02472353
BNP (Brain natriuretic peptide)	-	NCT02472353
Glutathione	-	NCT02472353
Cardiac function		TCTR20200116007
Cardiovascular hospitalizations	NCIC CTG MA.32 (Goodwin, 2022)	-
**Inflammation measures**		
hs-CRP (high sensitivity C-reactive protein)	Meyerhardt, 2020/Brown, 2020; SPIRIT Trial (Yeh, 2021); Reach for Health trial (Patterson, 2018); NCIC CTG MA.32 (Goodwin, 2022); Bonanni, 2012	-
sTNF-αR2 (soluble tumor necrosis factor alpha receptor 2)	Meyerhardt, 2020/Brown, 2020	-
IL-6 (interleukin 6)	Meyerhardt, 2020/Brown, 2020; SPIRIT Trial (Yeh, 2021);	-
Absolute counts of immune cell populations	-	OPTIMAL Trial (NCT04001725)
Relative counts of immune cell populations	-	OPTIMAL Trial (NCT04001725)
Activation status of immune cell populations	-	OPTIMAL Trial (NCT04001725)
Systemic inflammatory parameters	-	OPTIMAL Trial (NCT04001725)
**Tumor Biomarkers**		
Ki67	Sadighi, 2016; METEOR Trial (Kim, 2019); Semiglazova, 2018; NeoMet Trial (Hadad, 2011/Hadad, 2015); METTEN Trial (Martin-Castillo, 2018); Bonanni, 2012	METNEO Trial (NCT04170465); SETUP AIM Trial (ACTRN12612000416897); MetBreCS Trial (EUCTR2015-001001–14-IT)
CA-153	El-Haggar, 2016; NCIC CTG MA.32 (Goodwin, 2022)	-
Caspase-3	NeoMet Trial (Hadad, 2011/Hadad, 2015)	METNEO Trial (NCT04170465)
T-cell cytotoxic markers	-	NCT04387630
HER2-related pathways	METTEN Trial (Martin-Castillo, 2018)	-
**Liquid Biopsy**		
Circulating Tumor Cells (CTC)	MYME Trial (Nanni, 2018)	-
**Imaging responses**		
Mammogram	-	SETUP AIM Trial (ACTRN12612000416897)
Ultrasound	-	SETUP AIM Trial (ACTRN12612000416897)
Magnetic Resonance Imaging (MRI)	-	SETUP AIM Trial (ACTRN12612000416897)
**Cell signalling**		
pAMPK and pAkt	NeoMet Trial (Hadad, 2011/Hadad, 2015); METTEN Trial (Martin-Castillo, 2018)	-
AMPK phosphorylation		NCT02472353
mTOR	METTEN Trial (Martin-Castillo, 2018)	-
**Miscellaneous outcomes**		
Adiponectin	Bonanni, 2012	NCT02472353
Lipid peroxidation		NCT02472353
Leptin	Meyerhardt, 2020/Brown, 2020; NCIC CTG MA.32 (Goodwin, 2022)	-
Lipids profile	Ko, 2015	OPTIMAL Trial (NCT04001725)
Aminoacid profile	-	OPTIMAL Trial (NCT04001725)
Mitochondria function	-	TCTR20200116007
Brain local control rate of disease	-	OPTIMAL Trial (NCT04001725)
Neuropathy	-	NCT02360059
Feasibility	IBIS 3 Trial (Oke, 2017)	-
DLT (dose limiting toxicity)	Saif, 2019/Kritharis, 2014	-
PD (Pharmacodynamic markers)	Saif, 2019/Kritharis, 2014	-
BDI (Beck depression inventory)	Ko, 2015	
5 cognitive domains	Reach for Health trial (Patterson, 2018)	-
Glucocorticoids-induced changes in gut microbiota populations	-	OPTIMAL Trial (NCT04001725)
Metformin-induced changes in gut microbiota populations	-	OPTIMAL Trial (NCT04001725)
**Not specified miscellaneous outcomes**		
Not specified circulating and molecular biomarkers	-	MetBreCS Trial (EUCTR2015-001001–14-IT)
Not specified metabolomic analysis	-	MetBreCS Trial (EUCTR2015-001001–14-IT)
Not specified gene expression profile in adipose and epithelial breast tissue	-	MetBreCS Trial (EUCTR2015-001001–14-IT)
Not specified assessment of epigenetic changes in methylome patterns (DNA methylation)	-	MetBreCS Trial (EUCTR2015-001001–14-IT)

Clinical outcomes, including survival, quality of life, and cancer response rates, deserve special attention because they are considered the most important from the patients’ perspective. Therefore, we emphasize the presentation of results related to those outcomes in our narrative synthesis and figures.

#### Overall Survival (OS)

**Fig. 4:**
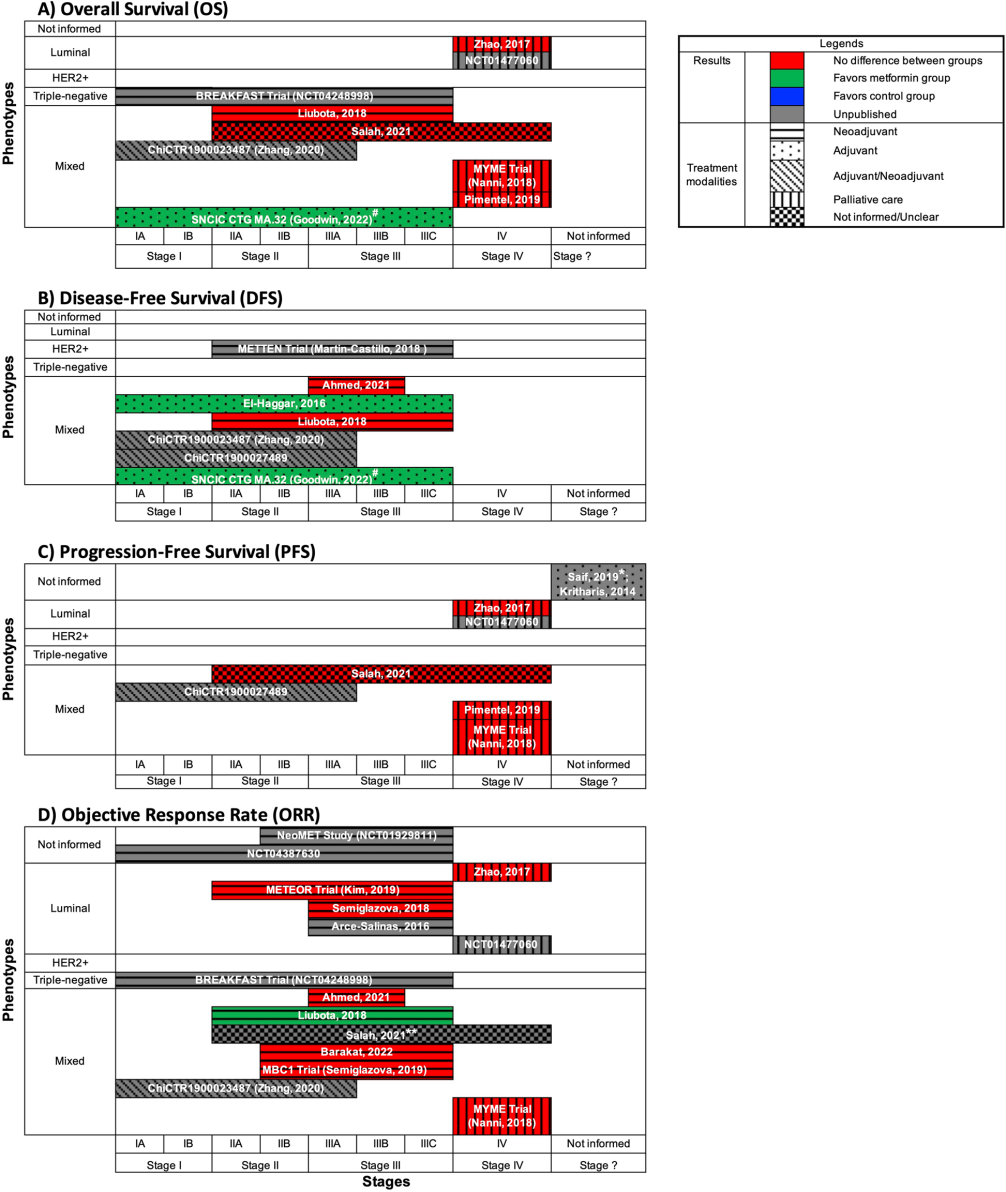
Results for overall survival, disease-free survival, progression-free survival, and objective response rate according to phenotype, breast cancer stage, and treatment modality adopted from primary studies # Positive for HER2 + phenotype only. * Included other types of cancer in addition to breast cancer. ** In the published article, the authors presented data on the frequency of partial response, stable disease and disease progression. However, they did not present complete response or partial response analyses of the overall response rate (ORR)

OS, defined as the time from randomization to death, is considered the ‘gold standard’ primary clinical endpoint in oncology [74]. Figure 4A depicts the mapping of the nine studies [22, 28, 43, 44, 46, 53, 68, 71, 75] that evaluated OS according to the phenotype, stage and treatment modality of breast cancer, as well as the publication status of their results concerning that outcome. Six [22, 28, 43, 44, 46, 53] of those nine studies had published results. Only one of those studies [28] showed improved survival among participants treated with metformin. Importantly, that effect was observed only among HER2 + participants (Hazard Ratio [HR] = 0.54; 95% CI: 0.30–0.98; P = 0.04) and not among participants with luminal breast cancer or the overall group of participants with mixed phenotypes.

#### Disease-free survival (DFS)

Seven studies [24, 28, 39, 40, 43, 62, 75] included DFS, defined as the time from randomization to disease recurrence [74], among their outcome measures. As shown in Fig. 4B, four [28, 39, 40, 43] of those seven studies had published results, and all of them included participants with mixed phenotypes. In two [28, 40] of them, there was some evidence of improvement in DFS among participants treated with metformin. Goodwin’s study [28] revealed improved DFS among HER2 + participants (HR = 0.64; 95% CI: 0.43–0.95; P = 0.03) but not among participants with other phenotypes or the overall group of participants. In the study by El-Haggar [40], which did not present DFS results for specific phenotypes, metformin treatment was associated with an average increase in DFS of approximately two months (log rank test, p = 0.044) and a hazard ratio of 0.31 (95% CI: 0.11–0.86, P = 0.023), favoring the metformin group for that same outcome in a statistical model adjusted for age, tumor stage, adjuvant chemotherapy, and estrogen and HER2 receptor status.

#### Progression-free survival (PFS)

PFS is defined as the time from randomization to disease progression or death [74]. Seven studies [22, 44, 46, 52, 53, 62, 71] reported PFS (Fig. 4C). Four studies [22, 44, 46, 53] out of those seven had some published results, all of which were negative. One of those studies [46] included only participants with luminal breast cancer, whereas the other three [22, 44, 53] included people with mixed phenotypes. Although PFS is an outcome used to assess therapies targeting advanced or metastatic malignancies [74], we noticed that one [44] of those four studies included participants with earlier stages of breast cancer in addition to metastatic disease. The same issue occurred with a study with unpublished results [62]. Notably, Saif’s study [52], which included participants with 16 different cancers, did not provide results specific to patients with breast cancer in its published article. Furthermore, although that study claimed to have evaluated PFS using the Kaplan‒Meier method, such results were not presented in the publication.

#### Objective response rate (ORR)

ORR is an outcome primarily used to assess neoadjuvant therapies and is defined as the proportion of patients who achieve a partial or complete response to therapy [74]. Fifteen studies [22, 39, 43–47, 54, 57, 58, 68–71, 75] evaluated ORR among their outcome measures (Fig. 4D). Eight [22, 39, 43, 45–47, 57, 58] of those 15 studies had published results, and only one [43] of them was positive. In that study, which included patients with mixed phenotypes and stages II-A to III-C breast cancer treated with neoadjuvant chemotherapy, the authors found that 28 (77.5%) out of 36 participants in the metformin arm achieved either a complete or partial response, in contrast to 9 (25%) out of 36 participants in the control arm (p < 0.05). Notably, of the eight studies with published results, five [22, 39, 43, 45, 58] included participants with mixed phenotypes, and three [46, 47, 57] included participants with the luminal phenotype; the latter of which were all negative.

#### Clinical benefit rate (CBR)

**Fig. 5:**
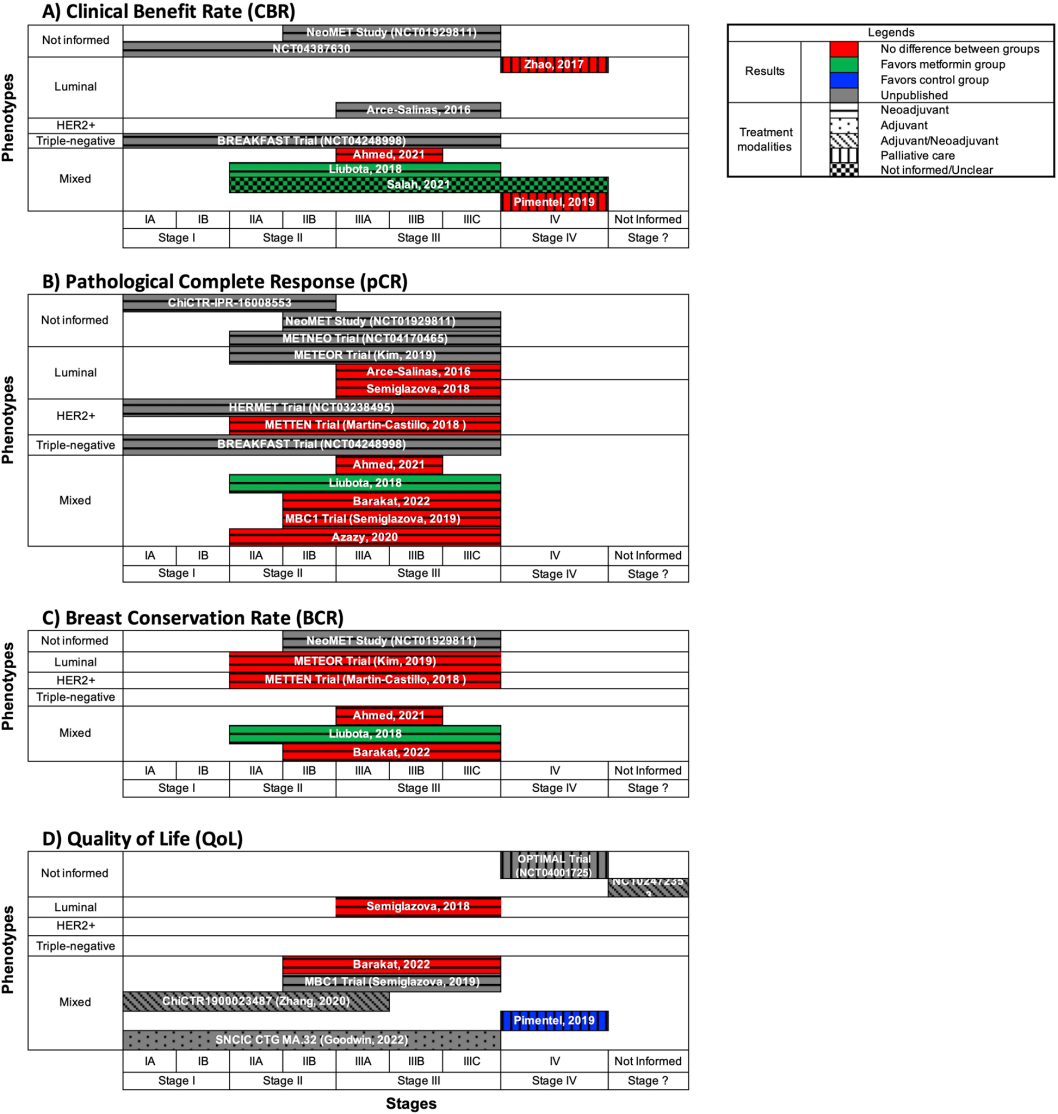
Results for the Clinical Benefit Rate, Pathological Complete Response, Breast Conservation Rate, and Quality of Life Outcomes According to Phenotype, Breast Cancer Stage, and Treatment Modality Adopted by Primary Studies

CBR is defined as the proportion of patients who achieve a complete response, partial response, or stable disease for at least six months [74]. Nine studies [39, 43, 44, 46, 53, 54, 68–70] included the CBR in their outcomes (Fig. 5A), and five [39, 43, 44, 46, 53] of them had published results. Only two [43, 44] of those studies reported positive results for that outcome. Both included participants with mixed phenotypes. Liubota’s study [43] included women with stages II to III disease who were receiving neoadjuvant chemotherapy and revealed a statistically significant increase in the proportion of patients with stable disease and complete or partial response (34 [94.5%] out of 36 in the metformin arm vs. 28 [78%] out of 36 in the control group, *p* < 0.05). The study by Salah et al. [44] included 50 participants with stage II to IV breast cancer. Unfortunately, the numbers of participants who experienced partial response or stable disease were reported on a three-dimensional figure, which does not allow the accurate extraction of data. Zhao’s trial [46] was the only study with published results restricted to participants with luminal breast cancer (*N* = 60). In that study, metformin was added to palliative chemotherapy for women with stage IV breast cancer and was not associated with a significantly different CBR compared with that of the control group.

#### Pathological Complete Response (pCR)

pCR is defined as the lack of residual invasive cancer in resected breast tissue or regional lymph nodes [74]. This outcome is commonly used for the accelerated approval of neoadjuvant therapies targeting breast cancer. Eight [24, 39, 43, 45, 47, 54, 55, 58] out of 14 studies [24, 39, 43, 45, 47, 54, 55, 57, 58, 63, 66–68, 70] had some published results on this outcome, and only one [43] of them, which included patients with mixed phenotypes and stage II to III breast cancer, was positive (Fig. 5B). In that study, 9 (26.5%) out of 36 patients in the metformin arm achieved pCR, while 2 (6%) out of 36 patients in the control group achieved pCR (p < 0.05). Of the remaining negative studies, four [39, 45, 55, 58] involved participants with mixed breast cancer, one [24] was restricted to participants with the HER2 + phenotype, and two [47, 54] focused on patients with luminal breast cancer.

#### Breast Conservation Rate (BCR)

Neoadjuvant systemic therapy for operable breast cancer can increase the options for conservative surgery in patients with breast cancer rather than mastectomy [76]. Six studies [24, 39, 43, 45, 57, 70] included BCR in their outcomes (Fig. 5C), and five [24, 39, 43, 45, 57] of them had published results. The only positive result came from Liubota’s study [43], which was already mentioned as the single study with positive ORR and pCR results. In that study, among the 35 participants with surgical indications, breast-conserving surgery was performed on 9 (50%) of the 18 patients in the metformin group and 4 (23.5%) of the 17 patients in the control group (p < 0.05).

#### Quality of Life (QoL)

Eight studies [28, 45, 47, 53, 58, 59, 72, 75] included QoL among their outcome measures (Fig. 5D). Three [45, 47, 53] of those eight studies had published results, and all of them used the European Organization for Research and Treatment for Cancer Quality of Life Questionnaire (EORTC-QLQ-C30). Only Pimentel’s study [53], which included 40 women with metastatic breast cancer with mixed phenotypes, identified a statistically significant difference regarding one of the domains of quality of life evaluated by that instrument. Remarkably, participants in the metformin arm experienced a large worsening of their Global Health Status in comparison with those in the control arm (standardized difference of 0.8, p = 0.006). Neither the other domains of quality of life nor the frequency of fatigue, diarrhea, nausea and vomiting, appetite loss, abdominal pain, or other symptoms measured by the EORT-QLQ-C30 were significantly different between the two groups in that study.

#### Adverse Events

Among the 25 studies with published results, 22 [21, 22, 24, 28, 35, 39–42, 44–46, 48–57] provided data on adverse events related to metformin compared to control groups, while three [43, 47, 58] did not report adverse events in their trial results.

The NCIC CTG MA.32 [28] was the only study in which participants in the metformin arm experienced a greater rate of severe adverse events. In that study, 391 (21.5%) patients in the metformin group and 328 (17.5%) in the placebo group experienced grade 3 or higher adverse events (P = 0.003); the most common adverse events of grade 3 or higher included hypertension (2.4% metformin vs 1.9% placebo), irregular menses (1.5% metformin vs 1.4% placebo), and diarrhea (1.9% metformin vs 0.8% placebo). In contrast, in the MYME trial [22], the metformin group experienced a lower frequency of grade 3 or 4 adverse events than did the control group, with 54% vs 72% of patients developing neutropenia, respectively (P = 0.019).

For the other 20 studies [21, 24, 35, 39–42, 44–46, 48–57] with published results, metformin was generally well tolerated and the incidence of severe adverse events was not greater in the metformin group than in the control group. However, as expected due to the well-known side effect profile of metformin, several studies have reported a greater rate of less severe (grades 1 and 2) gastrointestinal adverse events, such as diarrhea, associated with this medication.

#### Funding

Thirty-four studies [21, 24, 28, 35, 41, 42, 45–52, 54–60, 62–73, 75] were conducted with not-for-profit funding, such as research foundations, governments, and universities. Only two [22, 53] studies had mixed funding sources, including for-profit and not-for-profit funders. The MYME Trial [22] was funded by the Italian Association for Cancer Research (AIRC) and TEVA Pharmaceuticals. In addition, Pimentel’s study [53] was funded by the Breast Cancer Research Foundation, Hold’em for Life Charity, and the contract research organization Ozmosis Research Inc. Three studies [39, 40, 43] did not provide information regarding their sources of funding. Finally, Salah’s trial [44], which also included published results, was the only study in which its authors explicitly stated that it was conducted without sponsorship.

## Discussion

This is the most extensive and comprehensive review of RCTs that evaluated the use of metformin in the treatment of breast cancer. Our results provide insight into the growing body of literature that assesses the impact of metformin on the effectiveness of breast cancer treatment. We categorized existing studies based on breast cancer tumor phenotypes, stages, and possible treatment modalities. To the best of our knowledge, this comprehensive mapping and organization has not been previously undertaken by other studies of a similar nature. This charting effort is essential because it highlights knowledge gaps in the literature, identifying opportunities for new clinical trials and demonstrating the value and scope of further systematic reviews.

Based on the findings of this review, we identified the need for the development of methodological guidelines that ensure the clear presentation of breast cancer phenotype-specific data in any new oncological clinical trial in this field. We believe that obtaining trial results from the perspective of cancer phenotypes will make the evidence more relevant to clinical oncology practice, which frequently addresses a variety of breast tumor types. Until the development and implementation of such guidelines becomes a reality, we suggest that researchers explore the possibility of retrospectively ascertaining the phenotypes of participants from previous primary studies. If feasible, this approach has the strategic potential to rapidly generate new phenotype-specific data without incurring the substantial cost of recruiting new patients for novel trials. Furthermore, the accumulation of new phenotype-specific evidence from such retrospective analyses of previously completed clinical trials may be compiled in future systematic reviews. This compilation may provide strong enough evidence to support not only clinical decisions but also regulatory decisions.

Despite the variety of phenotypes, outcomes, disease states, treatment modalities, comparators, and publication status, our mapping of the field allows the envisioning of future phenotype- and outcome-specific meta-analyses. For example, there are six studies with published results, five of which involved mixed phenotypes and one focused on the luminal population (Fig. 4A), which can be used to conduct a meta-analysis on OS. Additionally, an opportunity exists to evaluate DFS using the METTEN trial [24] results for the HER2 + population and the four studies already published, which involve mixed phenotypes (Fig. 4B). Moreover, as previously highlighted, if future systematic reviewers are able to gain access to retrospectively ascertained phenotypes of breast cancer patients enrolled in previous RCTs, better and more meta-analyses would be possible, including performing individual patient data (IPD) meta-analyses taking that kind of information into account through a collaborative effort among researchers in this field.

With regard to opportunities for new clinical trials in this field, our review also allows the identification of relevant knowledge gaps to be addressed. For example, although there are already studies involving neoadjuvant treatment modalities for all breast cancer phenotypes, the landscape is not the same for adjuvant, or palliative studies, as depicted in Fig. 2. For example, innovative trials could be proposed to investigate the effectiveness of metformin within adjuvant and palliative treatment modalities in patients with HER2 + and triple-negative breast cancer. There is also a lack of evidence of adjuvant treatment with metformin specifically for the luminal phenotype.

We believe it is essential to propose new RCTs that combine phenotypes that have been poorly explored with the most important outcomes for breast cancer patients. Currently, the METTEN Trial [24] is the only completed trial with published results that investigated neoadjuvant treatment with metformin in patients with the HER2 + phenotype. Additionally, the only other trial involving the same phenotype and treatment modality remains unpublished [66]. Both trials had pCR as the primary outcome. Therefore, we believe there is an opportunity for more neoadjuvant trials that aim to evaluate HER2 + breast tumors, focusing on strategic clinical outcomes such as DFS and BCR. Further evaluation of metformin in patients with HER2 + breast cancer is especially relevant due to the limited evidence from a subgroup analysis of a single large RCT suggesting that it could improve OS and DFS [28]. In a similar vein, the BREAKFAST Trial [68] is the only interventional study proposed to investigate the effect of metformin on triple-negative breast cancer treated with a neoadjuvant modality. At the time of our last literature search, its results had not been published yet. As a result, further investigations into this specific phenotype are needed, with a focus on outcomes such as OS, ORR, pCR, BCR, and relapse-free survival.

Other relevant gaps in the literature that deserve mention include how treatment with metformin interacts with certain comorbidities and characteristics of participants such as overweight, obesity, metabolic syndrome, menopause status, and their physical activity levels. Such gaps may be addressed by new studies collecting that kind of data, reanalysis of existing data from specific studies, or even by IPD meta-analyses. This is particularly relevant because metformin may be effective among certain groups of people but not others.

Importantly, our review offers a map of the landscape of RCT data on metformin for the treatment of breast cancer. It is beyond the scope of our discussion section to outline every possible knowledge gap and research opportunity available in terms of new RCTs or systematic reviews. Experts in this field will be able to use our charting effort to recognize further research gaps and opportunities just as expert travelers are able to devise new paths from triangulating information from maps and their own knowledge of a specific region. For example, the examination of Figs. 4 and 5 easily reveals a lack of published results on the most important clinical outcomes for people with triple-negative breast cancer throughout all stages of the disease.

There are several major differences between our scoping review and previous reviews published on metformin and cancer. For instance, a systematic review [10] focused on in vitro and/or in vivo studies exploring the potential antiproliferative mechanisms of metformin. This review provided evidence of the effectiveness of metformin in cancer cell lines and/or animal models, confirming its antiangiogenic properties, as well as its ability to inhibit cellular metastasis and induce apoptosis.

Another prior systematic review of 11 observational studies [9] analyzed the association between metformin use by diabetic women and the prognosis of breast cancer patients and revealed that the use of the drug is associated with better survival of breast cancer patients with diabetes (HR: 0.53; 95% CI: 0.39–0.71; p < 0.001). However, meta-analyses of observational studies are subject to bias due to residual confounding within the primary studies included.

A single systematic review and meta-analysis attempted to assess the evidence from RCTs on the effectiveness of metformin in the treatment of breast cancer [11]. However, that review had several methodological limitations, including the absence of a registration of the review protocol, poor details of the search strategy for any database, and the exclusion of important databases and gray literature. Furthermore, there was insufficient description of the eligibility criteria, no flow diagram of the study selection process, no assessment of the certainty of evidence, and a limited range of evaluated outcomes. Importantly, that systematic review did not consider the different phenotypes, the staging of breast cancer, or the treatment modalities under which metformin was used in the primary studies. These aspects are crucial for interpreting the effectiveness of not only metformin but also any breast cancer treatment. Hence, it is likely that their meta-analyses were biased by too much clinical heterogeneity that compromised the quality of their pooled results. These limitations of the single systematic review of RCTs of metformin for the treatment of breast cancer corroborate the relevance of our scoping review in paving the way for future systematic reviews and clinical trials in this field.

Our review has some limitations. First, the absence of risk of bias evaluation of included studies, the appraisal of overall certainty of evidence across studies, or even the performance of a meta-analysis as a form of quantitative synthesis. All three aspects are possible but lie beyond the scope of a scoping review, which aims to provide a broad overview and description of the general landscape of a field. Second, we did not contact any author from the original studies to request unpublished data because our aim was to chart the literature, including its areas of uncertainties. Requesting unpublished data such as study results stratified by phenotype will be better suited to the future systematic reviews that we envision our scoping review will foster. Third, primary prevention of breast cancer also fell outside the scope of our review. The primary prevention and treatment of breast cancer are complex and involve different populations, risk factors, and biological determinants.

The present study also has relevant strengths. We established broad eligibility criteria without restricting the context, language, or publication date of the study reports because the best practice for a scoping review is to attempt to be as comprehensive as possible. We also searched gray literature and registers such as ICTRP, as well as using other methods such as Google Scholar, checking reference lists of relevant publications, reviewing conference abstract books, and seeking specialist referrals. These searches were crucial for identifying a large number of relevant study reports for this review. Additionally, two independent reviewers conducted the selection of studies and data extraction, while a third reviewer was available to resolve any disagreements that arose between the reviewers during that process.

## Conclusion

In summary, the proposed scoping review revealed a growing body of evidence from RCTs about the use of metformin for the treatment of breast cancer. We mapped the landscape of existing studies according to phenotypes, staging, treatment modality, types of interventions, comparators, outcomes, and their main findings. Given the clinical heterogeneity underlying breast cancer itself and the current existence of 40 different primary studies in this field, by charting that literature, we were able to identify new opportunities for clinical trials and systematic reviews. Specifically, we emphasize the necessity for standardizing the presentation of results from breast cancer clinical trials by phenotype and envision the potential for collaboration among researchers to retrospectively ascertain the phenotypes of breast cancer participants in previous studies. This could substantially enhance the possibility of conducting better and more cost-effective meta-analyses in this field, including IPD meta-analyses.

## Supplementary Information

Supplementary Material 1. Search strategy.

Supplementary Material 2. PRISMA-ScR checklist.

Supplementary Material 3. List of excluded reports with reasons for exclusion.

Supplementary Material 4. Main characteristics of the 15 included studies without published results (as of September 2023).

Supplementary Material 5. Clinical, non-clinical, and miscellaneous outcome measures examined by each study presented according to the breast cancer phenotype, stage, and treatment modality of their samples.

## Data Availability

All data and analyses pertaining to this scoping review are presented within the manuscript and supplemental files.
