# Supplementary material for: Metformin for the treatment of breast cancer: a scoping review of randomized clinical trials
Source: BMC Cancer. 2025 Aug 21;25:1352. doi: 10.1186/s12885-025-14468-3 (PMC12369099; doi:10.1186/s12885-025-14468-3)
Supplement: Supplementary file 1 — Supplementary Material 1. Search strategy. [file 12885_2025_14468_MOESM1_ESM.docx]

**Supplement 1: Search strategy**

# MEDLINE (via Pubmed)

| Search ID # | Search formula |
| --- | --- |
| 1 | (Metformin [Mesh]) |
| 2 | (Dimethylbiguanidine [all fields] OR Dimethylguanylguanidine [all fields] OR Glucophage [all fields]) |
| 3 | (Metformin Hydrochloride [all fields] OR Hydrochloride, Metformin [all fields] OR Metformin HCl [all fields] OR HCl, Metformin [all fields]) |
| 4 | #1 OR #2 OR #3 |
| 5 | (Breast Neoplasms [MeSH]) |
| 6 | (Breast Neoplasm [all fields] OR Neoplasm, Breast [all fields] OR Neoplasms, Breast [all fields]) |
| 7 | (Breast Tumors [all fields] OR Breast Tumor [all fields] OR Tumor, Breast [all fields] OR Tumors, Breast [all fields]) |
| 8 | (Breast Cancer [all fields] OR Cancer, Breast [all fields]) |
| 9 | (Cancer of Breast [all fields] OR Cancer of the Breast [all fields]) |
| 10 | (Mammary Cancer [all fields] OR Cancer, Mammary [all fields] OR Cancers, Mammary [all fields] OR Mammary Cancers [all fields]) |
| 11 | (Malignant Neoplasm of Breast [all fields] OR Breast Malignant Neoplasm [all fields] OR Breast Malignant Neoplasms [all fields]) |
| 12 | (Malignant Tumor of Breast [all fields] OR Breast Malignant Tumor [all fields] OR Breast Malignant Tumors [all fields]) |
| 13 | (Mammary Carcinoma, Human [all fields] OR Carcinoma, Human Mammary [all fields] OR Carcinomas, Human Mammary [all fields] OR Human Mammary Carcinomas [all fields] OR Mammary Carcinomas, Human [all fields] OR Human Mammary Carcinoma [all fields]) |
| 14 | (Mammary Neoplasms, Human [all fields] OR Human Mammary Neoplasm [all fields] OR Human Mammary Neoplasms [all fields] OR Neoplasm, Human Mammary [all fields] OR Neoplasms, Human Mammary [all fields] OR Mammary Neoplasm, Human [all fields]) |
| 15 | (Breast Carcinoma [all fields] OR Breast Carcinomas [all fields] OR Carcinoma, Breast [all fields] OR Carcinomas, Breast [all fields]) |
| 16 | #5 OR #6 OR #7 OR #8 OR #9 OR #10 OR #11 OR #12 OR #13 OR #14 OR #15 |
| 17 | (breast OR mammary) AND (cancer* OR tumour* OR tumor* OR neoplas* OR malignan* OR carcinoma*) |
| 18 | #16 OR #17 |
| 19 | #4 AND #18 |
| 20 | randomized controlled trial [pt] |
| 21 | controlled clinical trial [pt] |
| 22 | randomized [tiab] |
| 23 | placebo [tiab] |
| 24 | drug therapy [sh] |
| 25 | randomly [tiab] |
| 26 | trial [tiab] |
| 27 | groups [tiab] |
| 28 | #20 OR #21 OR #22 OR #23 OR #24 OR #25 OR #26 OR # 27 |
| 29 | animals [mh] NOT humans [mh] |
| 30 | #28 NOT #29 |
| 31 | #19 AND #30 |

# EMBASE

| Search # | Search formula |
| --- | --- |
| 1 | ‘metformin’/exp OR (1, 1 dimethylbiguanide) OR apophage OR aron OR benofomin OR dabex OR denkaform OR deson OR dextin OR diabetase OR (diabetase s) OR diabetformin OR diabetmin OR diabetmin retard OR diabetosan OR diabex OR diafat OR diaformin OR diaformina OR (diaformina lp) OR diametin OR diamin OR dianben OR diformin OR (diformin retard) OR dimefor OR dimethylbiguanide OR dimethyldiguanide OR dmgg OR dybis OR eraphage OR (espa-formin) OR (euform retard) OR fluamine OR flumamine OR fornidd OR fortamet OR glafornil OR glibudon OR glifage OR gliguanid OR glucaminol OR glucofage OR glucofago OR glucoform OR glucoformin OR glucohexal OR glucoless OR glucomet OR glucomin OR glucomine OR gluconil OR glucophage OR (glucophage forte) OR (glucophage retard) OR (glucophage sr) OR (glucophage xr) OR (glucophage xr extended release) OR (glucophage-mite) OR glucostop OR glucotika OR gludepatic OR glufor OR gluformin OR glukophage OR glumeformin OR glumet OR glumetza OR glupa OR glustress OR glyciphage OR glycomet OR glycon OR glycoran OR glyformin OR glymet OR haurymellin OR hipoglucin OR (i-max) OR islotin OR juformin OR (la 6023) OR la6023 OR (lyomet (drug)) OR maformin OR meglucon OR meguan OR melbin OR melformin OR mellittin OR merckformin OR mescorit OR metaformin OR metfogamma OR (metfoliquid geriasan) OR metforal OR metformax OR (metformin hydrochloride) OR metformina OR metformine OR (metformine hcl) OR methformin OR metiguanide OR metomin OR metphormin OR miformin OR (n' dimethylguanylguanide) OR (n' dimethylguanylguanidine) OR (n', n' dimethyldiguanide) OR (n, n dimethyl biguanidine) OR (n, n dimethylbiguanide) OR (n, n dimethylbiguanide retard) OR (n, n dimethylbiguanidine) OR (n, n dimethyldiguanide) OR (n, n dimethylguanylguanidine) OR neoform OR nndg OR (reglus-500) OR riomet OR (riomet er) OR risidon OR siamformet OR siofor OR thiabet OR vimetrol OR walaphage |
| 2 | (‘breast tumor’)/exp OR (breast gland tumor) OR (breast gland tumour) OR (breast mass) OR (breast neoplasms) OR (breast neoplasms, male) OR (breast tumour) OR (female breast neoplasm) OR (female breast tumor) OR (female breast tumour) OR (male breast neoplasms) OR (mamma tumor) OR (mamma tumour) OR (mammary gland tumor) OR (mammary gland tumour) OR (mammary neoplasms) OR (mammary tumor) OR (mammary tumor cell) OR (mammary tumour) OR (mammary tumour cell) OR (unilateral breast neoplasms) |
| 3 | (‘breast carcinoma’)/exp OR (carcinoma, infiltrating duct) OR (carcinoma, mammary) OR (infiltrating duct carcinoma) OR (invasive ductal carcinoma) OR (mamma carcinoma) OR (mammary carcinoma) |
| 4 | #2 OR #3 |
| 5 | #1 AND #4 |
| 6 | 'double-blind procedure':de OR 'randomized controlled trial':de OR 'single-blind procedure':de OR (random* OR factorial* OR placebo* OR doubl* NEAR/1 blind* OR singl* NEAR/1 blind* OR assign* OR allocat* OR volunteer*):de,ab,ti |
| 7 | #5 AND #6 AND [embase]/lim NOT [medline]/lim |

# LILACS

| Search # | Search formula |
| --- | --- |
| 1 | (Metformin OR Metformina OR Metformina OR Dimetil Guanil Guanidina) |
| 2 | (Breast Neoplasms OR Neoplasias de la Mama OR Neoplasias da Mama OR Carcinoma Mamário Humano OR Carcinoma de Mama OR Carcinomas Mamários Humanos OR Carcinomas da Mama OR Câncer Mamário OR Câncer da Mama OR Câncer de Mama OR Câncer de Seio OR Câncer do Seio OR Cânceres de Mama OR Neoplasia Maligna da Mama OR Neoplasia Maligna de Mama OR Neoplasia Mamária OR Neoplasia Mamária Humana OR Neoplasia da Mama OR Neoplasias Malignas de Mama OR Neoplasias Mamárias OR Neoplasias Mamárias Humanas OR Neoplasias de Mama OR Tumor Maligno da Mama OR Tumor da Mama OR Tumor de Mama OR Tumor de Seio OR Tumores Malignos da Mama OR Tumores Mamários OR Tumores da Mama OR Tumores de Mama OR Tumores de Seio) |
| 3 | (Randomized Controlled Trial OR Ensayo Clínico Controlado Aleatorio OR Ensaio Clínico Controlado Aleatório OR Ensaio Clínico Controlado Randomizado OR Ensaio Controlado Aleatório) |
| 4 | #1 AND #2 AND #3 |

# Web of Science

| Search ID # | Search formula |
| --- | --- |
| 1 | TS= (Metformin) |
| 2 | TS= (Dimethylbiguanidine OR Dimethylguanylguanidine OR Glucophage) |
| 3 | TS= (“Metformin Hydrochloride” OR “Hydrochloride, Metformin” OR “Metformin HCl” OR “HCl, Metformin”) |
| 4 | #1 OR #2 OR #3 |
| 5 | TS= (Breast Neoplasms) |
| 6 | TS= (“Breast Neoplasm” OR “Neoplasm, Breast” OR “Neoplasms, Breast”) |
| 7 | TS= (“Breast Tumors” OR “Breast Tumor” OR “Tumor, Breast” OR “Tumors, Breast”) |
| 8 | TS= (“Breast Cancer” OR “Cancer, Breast”) |
| 9 | TS= (“Cancer of Breast” OR “Cancer of the Breast”) |
| 10 | TS= (“Mammary Cancer” OR “Cancer, Mammary” OR “Cancers, Mammary” OR “Mammary Cancers”) |
| 11 | TS= (“Malignant Neoplasm of Breast” OR “Breast Malignant Neoplasm” OR “Breast Malignant Neoplasms”) |
| 12 | TS= (“Malignant Tumor of Breast” OR “Breast Malignant Tumor” OR “Breast Malignant Tumors”) |
| 13 | TS= (“Mammary Carcinoma, Human” OR “Carcinoma, Human Mammary” OR “Carcinomas, Human Mammary” OR “Human Mammary Carcinomas” OR “Mammary Carcinomas, Human” OR “Human Mammary Carcinoma”) |
| 14 | TS= (“Mammary Neoplasms, Human” OR “Human Mammary Neoplasm” OR “Human Mammary Neoplasms” OR “Neoplasm, Human Mammary” OR “Neoplasms, Human Mammary” OR “Mammary Neoplasm, Human”) |
| 15 | TS= (“Breast Carcinoma” OR “Breast Carcinomas” OR “Carcinoma, Breast” OR “Carcinomas, Breast”) |
| 16 | #5 OR #6 OR #7 OR #8 OR #9 OR #10 OR #11 OR #12 OR #13 OR #14 OR #15 |
| 17 | TS= (breast OR mammary) AND (neoplas* OR tumor* OR tumour* OR cancer* OR malignan* OR carcinoma*) |
| 18 | #16 OR #17 |
| 19 | #4 AND #18 |
| 20 | TS= (“Randomized Controlled Trial” OR “Controlled Clinical Trial” OR “Clinical Trial”) |
| 21 | #19 AND #20 |

# CENTRAL

| Search ID # | Search formula |
| --- | --- |
| 1 | Mesh descriptor: [Metformin] explode all trees |
| 2 | (Dimethylbiguanidine OR Dimethylguanylguanidine OR Glucophage) |
| 3 | (“Metformin Hydrochloride” OR “Hydrochloride, Metformin” OR “Metformin HCl” OR “HCl, Metformin”) |
| 4 | #1 OR #2 OR #3 |
| 5 | Mesh descriptor: [Breast Neoplasms] explode all trees |
| 6 | (“Breast Neoplasm” OR “Neoplasm, Breast” OR “Neoplasms, Breast”) |
| 7 | (“Breast Tumors” OR “Breast Tumor” OR “Tumor, Breast” OR “Tumors, Breast”) |
| 8 | (“Breast Cancer” OR “Cancer, Breast”) |
| 9 | (“Cancer of Breast” OR “Cancer of the Breast”) |
| 10 | (“Mammary Cancer” OR “Cancer, Mammary” OR “Cancers, Mammary” OR “Mammary Cancers”) |
| 11 | (“Malignant Neoplasm of Breast” OR “Breast Malignant Neoplasm” OR “Breast Malignant Neoplasms”) |
| 12 | (“Malignant Tumor of Breast” OR “Breast Malignant Tumor” OR “Breast Malignant Tumors”) |
| 13 | (“Mammary Carcinoma, Human” OR “Carcinoma, Human Mammary” OR “Carcinomas, Human Mammary” OR “Human Mammary Carcinomas” OR “Mammary Carcinomas, Human” OR “Human Mammary Carcinoma”) |
| 14 | (“Mammary Neoplasms, Human” OR “Human Mammary Neoplasm” OR “Human Mammary Neoplasms” OR “Neoplasm, Human Mammary” OR “Neoplasms, Human Mammary” OR “Mammary Neoplasm, Human”) |
| 15 | (“Breast Carcinoma” OR “Breast Carcinomas” OR “Carcinoma, Breast” OR “Carcinomas, Breast”) |
| 16 | #5 OR #6 OR #7 OR #8 OR #9 OR #10 OR #11 OR #12 OR #13 OR #14 OR #15 |
| 17 | (breast OR mammary) AND (neoplas* OR tumor* OR tumour* OR cancer* OR malignan* OR carcinoma*) |
| 18 | #16 OR #17 |
| 19 | #4 AND 18 |
