# Supplementary material for: Metformin for the treatment of breast cancer: a scoping review of randomized clinical trials
Source: BMC Cancer. 2025 Aug 21;25:1352. doi: 10.1186/s12885-025-14468-3 (PMC12369099; doi:10.1186/s12885-025-14468-3)
Supplement: Supplementary file 3 — Supplementary Material 3. List of excluded reports with reasons for exclusion. [file 12885_2025_14468_MOESM3_ESM.docx]

# Supplement 3: List of excluded reports with reasons for exclusion

# Excluded Reports from Databases (MEDLINE, EMBASE, Web of Science, LILACS, and CENTRAL)

| 7 reports excluded from Databases | | | |
| --- | --- | --- | --- |
|  | Author, data | Reference | Reason of exclusion |
| 1 | Carlo C. and Pasanisi P. 2011 | Carlo C., Pasanisi P. Metformin and breast cancer. Climacteric. 2011;14((Carlo C.; Pasanisi P.)):21. | wrong comparator |
| 2 | Egle et al. 2017 | Egle D, Marth C, Steger G, Bartsch R, Pfeiler G, Greil R, et al. Effect of metformin on progression-free survival outcomes in patients with advanced or metastatic HR+, HER2-breast cancer: subgroup analysis of STEPAUT. Breast. 2017 Mar;32:S26–7. | wrong study design |
| 3 | Gennari A. et al. 2016 | Gennari A., Nanni O., Rocca A., De Censi A., Fieschi A., Bologna A., et al. Phase II randomised clinical study of metformin plus chemotherapy vs chemotherapy alone in HER2 negative metastatic breast cancer: final results of the MYME trial. Ann Oncol. 2016;27((Gennari A.; De Censi A.; Corradengo D.) Medical Oncology, Ospedali Galliera, Genoa, Italy):vi70. | duplicate |
| 4 | Goodwin et al. 2019 | Goodwin PJ, Ennis M, Cescon DW, Elser C, Haq R, Hamm CM, et al. Phase II randomized clinical trial (RCT) of metformin (MET) vs placebo (PLAC) in combination with chemotherapy (CXT) in refractory locally advanced (LABC) or metastatic breast cancer (MBC). Cancer Res [Internet]. 2019 Feb;79(4). Available from: ://WOS:000478677000305 | duplicate |
| 5 | MedSIR and Novartis 2020 | MedSIR, Novartis. Study to Evaluate the Effect of Metformin in the Prevention of HG in HR + /HER2 - PIK3CA-mut Advanced BC Patients. 2020 Oct 23; | wrong comparator |
| 6 | NCT01905046 | NCT01905046. Metformin Hydrochloride in Preventing Breast Cancer in Patients With Atypical Hyperplasia or In Situ Breast Cancer. https://clinicaltrials.gov/show/NCT01905046 [Internet]. 2013; Available from: https://www.cochranelibrary.com/central/doi/10.1002/central/CN-01489061/full | wrong population |
| 7 | Sehdev et al. 2018 | Sehdev A, Karrison T, Zha Y, Janisch L, Turcich M, Cohen E, et al. A pharmacodynamic study of sirolimus and metformin in patients with advanced solid tumors. Cancer Chemother Pharmacol. 2018;82(2):309‐317. | wrong comparator |

# Excluded Reports from the International Clinical Trial Registry Platform (ICTRP)

| 22 reports excluded from ICTRP | | | |
| --- | --- | --- | --- |
|  | Author, data | Reference | Reason of exclusion |
| 1 | Chow, 2022 | Chow S. Phase II Study of Metformin for Reduction of Obesity-Associated Breast Cancer Risk [Internet]. clinicaltrials.gov; 2022 Jun. Report No.: NCT02028221. Available from: https://clinicaltrials.gov/ct2/show/NCT02028221 | wrong population |
| 2 | Crew, 2022 | Crew KD. Pre-Surgical Trial of the Combination of Metformin and Atorvastatin in Newly Diagnosed Operable Breast Cancer [Internet]. clinicaltrials.gov; 2022 Aug. Report No.: NCT01980823. Available from: https://clinicaltrials.gov/ct2/show/NCT01980823 | wrong study design |
| 3 | Daiqin, 2019 | Daiqin L. Effect of metformin combined with radiotherapy on radiosensitivity of CD44+CD24- MCF-7 cells in breast cancer [Internet]. 2019. Report No.: ChiCTR1900023063. Available from: https://www.chictr.org.cn/showproj.aspx?proj=38759 | wrong study design |
| 4 | Davis, 2016 | Davis S. The effects of metformin on the LKB1/AMP-activated protein kinase (AMPK) pathway in breast tissue, a pilot study [Internet]. 2016. Report No.: ACTRN12610000219088. Available from: https://anzctr.org.au/Trial/Registration/TrialReview.aspx?ACTRN=12610000219088 | wrong population |
| 5 | Esserman, 2022 | Esserman L. I-SPY TRIAL: Neoadjuvant and Personalized Adaptive Novel Agents to Treat Breast Cancer [Internet]. clinicaltrials.gov; 2022 Nov. Report No.: NCT01042379. Available from: https://clinicaltrials.gov/ct2/show/NCT01042379 | wrong comparator |
| 6 | EUCTR2009-009921-28-IT | EUCTR2009-009921-28-IT. The Effect of Metformin, an insulin-sensitizing drug, on Breast Cancer Primary Prevention: The Plotina Breast Cancer Prevention Randomized, Placebo Controlled [Internet]. 2009. Available from: https://www.clinicaltrialsregister.eu/ctr-search/trial/2009-009921-28/IT | wrong population |
| 7 | Fedeli, 2020 | Fedeli A. A Study of Liposomal Doxorubicin + Docetaxel + Trastuzumab + Metformin in Operable and Locally Advanced HER2 Positive Breast Cancer [Internet]. clinicaltrials.gov; 2020 Apr. Report No.: NCT02488564. Available from: https://clinicaltrials.gov/ct2/show/NCT02488564 | wrong study design |
| 8 | Fedeli, 2021 | Fedeli A. Clinical and translational phase II study of liposomal doxorubicin plus docetaxel and transtuzumab with metformin as primary systemic therapy for operable and locally advanced HER2 positive breast cancer [Internet]. 2021. Report No.: EUCTR2014-002602-20-IT. Available from: https://www.clinicaltrialsregister.eu/ctr-search/trial/2014-002602-20/IT | wrong study design |
| 9 | Goodwin, 2012 | Goodwin PJ. Clinical and Biologic Effects of Metformin in Early Stage Breast Cancer [Internet]. clinicaltrials.gov; 2012 Jan. Report No.: NCT00897884. Available from: https://clinicaltrials.gov/ct2/show/NCT00897884 | wrong study design |
| 10 | Harris, 2014 | Harris A. Effect of Metformin on Breast Cancer Metabolism [Internet]. clinicaltrials.gov; 2014 Jul. Report No.: NCT01266486. Available from: https://clinicaltrials.gov/ct2/show/NCT01266486 | wrong study design |
| 11 | Hershman, 2022 | Hershman DL. Pre-Surgical Study: Effect of Metformin on Breast Cancer Proliferation [Internet]. clinicaltrials.gov; 2022 Oct. Report No.: NCT00930579. Available from: https://clinicaltrials.gov/ct2/show/NCT00930579 | wrong study design |
| 12 | Hoffmann, 2023 | Hoffmann. To Evaluate the Safety, Tolerability, and Pharmacokinetics of Inavolisib Single Agent in Participants With Solid Tumors and in Combination With Endocrine and Targeted Therapies in Participants With Breast Cancer [Internet]. clinicaltrials.gov; 2023 Feb. Report No.: NCT03006172. Available from: https://clinicaltrials.gov/ct2/show/NCT03006172 | wrong study design |
| 13 | Kalinsky, 2017 | Kalinsky K. Study of Erlotinib and Metformin in Triple Negative Breast Cancer [Internet]. clinicaltrials.gov; 2017 Aug. Report No.: NCT01650506. Available from: https://clinicaltrials.gov/ct2/show/NCT01650506 | wrong study design |
| 14 | Lohmann, 2016 | Lohmann AE. The Use of Metformin in Early Breast Cancer Patients Pre-Surgery [Internet]. clinicaltrials.gov; 2016 Feb. Report No.: NCT01302002. Available from: https://clinicaltrials.gov/ct2/show/NCT01302002 | wrong study design |
| 15 | Mackenzie, 2013 | Mackenzie M. Metformin and Temsirolimus in Treating Patients With Metastatic or Unresectable Solid Tumor or Lymphoma [Internet]. 2013. Report No.: NCT00659568. Available from: https://clinicaltrials.gov/ct2/show/NCT00659568?term=NCT00659568&draw=2&rank=1 | wrong study design |
| 16 | Mayer, 2012 | Mayer I. Metformin Hydrochloride in Treating Women With Stage I or Stage II Breast Cancer That Can Be Removed By Surgery [Internet]. clinicaltrials.gov; 2012 Jun. Report No.: NCT00984490. Available from: https://clinicaltrials.gov/ct2/show/NCT00984490 | wrong study design |
| 17 | Novartis Pharmaceuticals, 2023 | Novartis Pharmaceuticals. Study of Safety and Efficacy of Dapagliflozin + Metformin XR Versus Metformin XR in Participants With HR+, HER2-, Advanced Breast Cancer While on Treatment With Alpelisib and Fulvestrant [Internet]. clinicaltrials.gov; 2023 Jan. Report No.: NCT04899349. Available from: https://clinicaltrials.gov/ct2/show/NCT04899349 | wrong comparator |
| 18 | Ow, 2017 | Ow S. Metformin and Simvastatin in Addition to Fulvestrant [Internet]. clinicaltrials.gov; 2017 Jun. Report No.: NCT03192293. Available from: https://clinicaltrials.gov/ct2/show/NCT03192293 | wrong study design |
| 19 | Seidemann, 2022 | Seidemann, Ericka. Metformin Use to Reduce Disparities in Newly Diagnosed Breast Cancer Response to Neoadjuvant Treatment [Internet]. clinicaltrials.gov; 2022 Sep. Report No.: NCT04741204. Available from: https://clinicaltrials.gov/ct2/show/NCT04741204 | wrong comparator |
| 20 | Storniolo, 2018 | Storniolo AM. Metformin Hydrochloride vs. Placebo in Overweight or Obese Patients at Elevated Risk for Breast Cancer [Internet]. clinicaltrials.gov; 2018 Mar. Report No.: NCT01793948. Available from: https://clinicaltrials.gov/ct2/show/NCT01793948 | wrong population |
| 21 | Sundelin, 2018 | Sundelin EIO. Metformin in Breast Cancer, Visualized With Positron Emission Tomography [Internet]. clinicaltrials.gov; 2018 Jan. Report No.: NCT02882581. Available from: https://clinicaltrials.gov/ct2/show/NCT02882581 | wrong study design |
| 22 | Valero, 2020 | Valero V. Exemestane-RAD001-Metformin [Internet]. clinicaltrials.gov; 2020 Jun. Report No.: NCT01627067. Available from: https://clinicaltrials.gov/ct2/show/NCT01627067 | wrong study design |

# Excluded Reports from Google Scholar

| 32 reports excluded from Google Scholar | | | |
| --- | --- | --- | --- |
|  | Author, data | Reference | Reason of exclusion |
| 1 | Arce-Salinas et al. 2016 | Arce-Salinas C, Alamilla G, Flores-Diaz D, Deneken CZ, Mendoza-Galindo L, Ramirez-Morales R, et al. Randomized, double blind trial to evaluate the safety and efficacy of metformin vs placebo plus neoadjuvant chemotherapy in locally advanced breast cancer. J Clin Oncol [Internet]. 2016;34. Available from: https://www.embase.com/search/results?subaction=viewrecord&id=L611753653&from=export | duplicate |
| 2 | Bonanni et al. 2012 | Bonanni B, Puntoni M, Cazzaniga M, Pruneri G, Serrano D, Guerrieri-Gonzaga A, et al. Dual effect of metformin on breast cancer proliferation in a randomized presurgical trial. J Clin Oncol. 2012;30(21):2593‐2600. | duplicate |
| 3 | Brown et al. 2020 | Brown JC, Zhang S, Ligibel JA, Irwin ML, Jones LW, Campbell N, et al. Effect of Exercise or Metformin on Biomarkers of Inflammation in Breast and Colorectal Cancer: A Randomized Trial. Cancer Prev Res Phila Pa. 2020 Dec;13(12):1055–62. | duplicate |
| 4 | Cazzaniga et al. 2013 | Cazzaniga M, DeCensi A, Pruneri G, Puntoni M, Bottiglieri L, Varricchio C, et al. The effect of metformin on apoptosis in a breast cancer presurgical trial. Br J Cancer. 2013 Nov 26;109(11):2792–7. | duplicate |
| 5 | Cuyas et al. 2019 | Cuyas E, Buxo M, Ferri Iglesias MJ, Verdura S, Pemas S, Dorca J, et al. The C Allele of ATM rs11212617 Associates With Higher Pathological Complete Remission Rate in Breast Cancer Patients Treated With Neoadjuvant Metformin. Front Oncol [Internet]. 2019 Mar 28;9. Available from: ://WOS:000462704800001 | duplicate |
| 6 | Cuyàs et al. 2019 | Cuyàs E, Fernández-Arroyo S, Buxó M, Pernas S, Dorca J, Álvarez I, et al. Metformin induces a fasting- and antifolate-mimicking modification of systemic host metabolism in breast cancer patients. Aging. 2019 May 9;11(9):2874–88. | duplicate |
| 7 | Davis et al. 2018 | Davis SR, Robinson PJ, Jane F, White S, Brown KA, Piessens S, et al. The benefits of adding metformin to tamoxifen to protect the endometrium-A randomized placebo-controlled trial. Clin Endocrinol (Oxf). 2018 Nov;89(5):605–12. | duplicate |
| 8 | DeCensi et al. 2012 | DeCensi A, Pollak MN, Puntoni M, Gandini S, Cazzaniga M, Pruneri G, et al. Dual effects of metformin on breast cancer proliferation in a randomized trial. J Clin Oncol [Internet]. 2012;30(15). Available from: https://www.embase.com/search/results?subaction=viewrecord&id=L71002974&from=export | duplicate |
| 9 | DeCensi et al. 2014 | DeCensi A, Puntoni M, Gandini S, Guerrieri-Gonzaga A, Johansson HA, Cazzaniga M, et al. Differential effects of metformin on breast cancer proliferation according to markers of insulin resistance and tumor subtype in a randomized presurgical trial. Breast Cancer Res Treat. 2014 Nov;148(1):81–90. | duplicate |
| 10 | DeCensi et al. 2015 | DeCensi A, Puntoni M, Guerrieri-Gonzaga A, Cazzaniga M, Serrano D, Lazzeroni M, et al. Effect of Metformin on Breast Ductal Carcinoma In Situ Proliferation in a Randomized Presurgical Trial. Cancer Prev Res (Phila Pa). 2015 Oct;8(10):888–94. | duplicate |
| 11 | El-Haggar et al. 2016 | El-Haggar SM, El-Shitany NA, Mostafa MF, El-Bassiouny NA. Metformin may protect nondiabetic breast cancer women from metastasis. Clin Exp Metastasis. 2016 Apr;33(4):339–57. | duplicate |
| 12 | Gennari et al. 2020 | Gennari A, Foca F, Zamarchi R, Rocca A, Amadori D, De Censi A, et al. Insulin-like growth factor-1 receptor (IGF-1R) expression on circulating tumor cells (CTCs) and metastatic breast cancer outcome: results from the TransMYME trial. Breast Cancer Res Treat. 2020 May;181(1):61–8. | duplicate |
| 13 | Goodwin et al. 2013 | Goodwin PJ, Parulekar W, Gelmon KA, Shepherd LE, Ligibel JA, Hershman DL, et al. Effect of metformin versus placebo on weight and metabolic factors in initial patients enrolled onto NCIC CTG MA.32, a multicenter adjuvant randomized controlled trial in early-stage breast cancer (BC). J Clin Oncol [Internet]. 2013 May 20;31(15). Available from: ://WOS:000335419600256 | duplicate |
| 14 | Goodwin et al. 2019 | Goodwin PJ, Ennis M, Cescon DW, Elser C, Haq R, Hamm CM, et al. Phase II randomized clinical trial (RCT) of metformin (MET) vs placebo (PLAC) in combination with chemotherapy (CXT) in refractory locally advanced (LABC) or metastatic breast cancer (MBC). Cancer Res [Internet]. 2019 Feb;79(4). Available from: ://WOS:000478677000305 | duplicate |
| 15 | Goodwin et al. 2021 | Goodwin P, Dowling R, Ennis M, ... Cancer Antigen 15-3/Mucin 1 Levels in CCTG MA. 32: A Breast Cancer Randomized Trial of Metformin vs Placebo. JNCI Cancer … [Internet]. 2021;(Query date: 2021-09-30 09:48:10). Available from: https://academic.oup.com/jncics/article-abstract/5/5/pkab066/6329643 | duplicate |
| 16 | Hadad et al. 2010 | Hadad S, Dewar J, Elseedawy E, ... Gene signature of metformin actions on primary breast cancer within a window of opportunity randomized clinical trial. J Clin … [Internet]. 2010;(Query date: 2021-09-30 09:48:10). Available from: https://ascopubs.org/doi/abs/10.1200/jco.2010.28.15_suppl.560 | duplicate |
| 17 | Hadad et al. 2011 | Hadad S, Iwamoto T, Jordan L, Purdie C, Bray S, Baker L, et al. Evidence for biological effects of metformin in operable breast cancer: a pre-operative, window-of-opportunity, randomized trial. Breast Cancer Res Treat. 2011 Aug;128(3):783–94. | duplicate |
| 18 | Hadad et al. 2015 | Hadad SM, Coates P, Jordan LB, Dowling RJO, Chang MC, Done SJ, et al. Evidence for biological effects of metformin in operable breast cancer: biomarker analysis in a pre-operative window of opportunity randomized trial. Breast Cancer Res Treat. 2015 Feb;150(1):149–55. | duplicate |
| 19 | Johansson et al. 2012 | Johansson HA, De Censi A, Puntoni M, Cazzaniga M, Pruneri G, Serrano D, et al. Effects of metformin on markers of insulin resistance and on breast cancer proliferation: The putative role of IGFBP-1 as a predictive biomarker. Cancer Res [Internet]. 2012 Apr 15;72. Available from: ://WOS:000209701503213 | duplicate |
| 20 | Kim et al. 2014 | Kim J, Lim W, Kim E, Kim M, Paik N, Jeong S, et al. Phase II randomized trial of neoadjuvant metformin plus letrozole versus placebo plus letrozole for estrogen receptor positive postmenopausal breast cancer (METEOR). BMC Cancer. 2014;14:170. | duplicate |
| 21 | Kim et al. 2019 | Kim J, Han W, Kim E, Jung Y, Kim H, Chae S, et al. Phase II randomized study of neoadjuvant metformin plus letrozole versus placebo plus letrozole for ER-positive postmenopausal breast cancer [METEOR Study]. [Internet]. ascopubs.org; 2019. Available from: https://ascopubs.org/doi/abs/10.1200/JCO.2019.37.15_suppl.576 | duplicate |
| 22 | Ko et al. 2015 | Ko K, Ma S, Yang J, Hwang Y, Ahn C, Cho Y, et al. Metformin intervention in obese non-diabetic patients with breast cancer: phase II randomized, double-blind, placebo-controlled trial. Breast Cancer Res Treat. 2015;153(2):361‐370. | duplicate |
| 23 | Lohmann et al. 2017 | Lohmann A, Liebman M, Brien W, Parulekar W, Gelmon K, Shepherd L, et al. Effects of metformin versus placebo on vitamin B12 metabolism in non-diabetic breast cancer patients in CCTG MA.32. Breast Cancer Res Treat. 2017;164(2):371‐378. | duplicate |
| 24 | Lopez-Bonet E. et al. 2019 | Lopez-Bonet E., Buxó M., Cuyàs E., Pernas S., Dorca J., Álvarez I., et al. Neoadjuvant metformin added to systemic therapy decreases the proliferative capacity of residual breast cancer. J Clin Med [Internet]. 2019;8(12). Available from: http://www.embase.com/search/results?subaction=viewrecord&from=export&id=L2003271278 | duplicate |
| 25 | Meyerhardt et al. 2020 | Meyerhardt JA, Irwin ML, Jones LW, Zhang S, Campbell N, Brown JC, et al. Randomized phase II trial of exercise, metformin, or both on metabolic biomarkers in colorectal and breast cancer survivors. JNCI Cancer Spectr [Internet]. 2020;4(1). Available from: https://www.embase.com/search/results?subaction=viewrecord&id=L631824359&from=export | duplicate |
| 26 | Nanni et al. 2019 | Nanni O, Amadori D, De Censi A, Rocca A, Freschi A, Bologna A, et al. Metformin plus chemotherapy versus chemotherapy alone in the first-line treatment of HER2-negative metastatic breast cancer. The MYME randomized, phase 2 clinical trial. Breast Cancer Res Treat. 2019 Apr;174(2):433–42. | duplicate |
| 27 | Parulekar et al. 2011 | Parulekar W, Chen B, Elliott C, ... A phase III randomized trial of metformin versus placebo on recurrence and survival in early-stage breast cancer (BC)(NCIC Clinical Trials Group MA. 32). J Clin … [Internet]. 2011;(Query date: 2021-09-30 09:48:10). Available from: https://ascopubs.org/doi/abs/10.1200/jco.2011.29.15_suppl.tps103 | duplicate |
| 28 | Patterson et al. 2016 | Patterson RE, Marinac CR, Natarajan L, Hartman SJ, Cadmus-Bertram L, Flatt SW, et al. Recruitment strategies, design, and participant characteristics in a trial of weight-loss and metformin in breast cancer survivors. Contemp Clin Trials. 2016 Mar;47:64–71. | duplicate |
| 29 | Patterson et al. 2018 | Patterson RE, Marinac CR, Sears DD, Kerr J, Hartman SJ, Cadmus-Bertram L, et al. The Effects of Metformin and Weight Loss on Biomarkers Associated With Breast Cancer Outcomes. J Natl Cancer Inst. 2018 Nov 1;110(11):1239–47. | duplicate |
| 30 | Pimentel et al. 2019 | Pimentel I, Lohmann AE, Ennis M, Dowling RJO, Cescon D, Elser C, et al. A phase II randomized clinical trial of the effect of metformin versus placebo on progression-free survival in women with metastatic breast cancer receiving standard chemotherapy. Breast. 2019 Dec;48:17–23. | duplicate |
| 31 | Zhang et al. 2020 | Zhang J, Ma X, Li Y, Liu R, Li Y, Zhang P, et al. Metformin intervention against ovarian toxicity during chemotherapy for early breast cancer: study protocol for a randomized double-blind placebo-controlled …. Maturitas [Internet]. 2020;(Query date: 2021-09-30 09:48:10). Available from: https://www.sciencedirect.com/science/article/pii/S0378512220302401?casa_token=UkVFDHR2g3YAAAAA:slHgMVpODkTitGg9cKJCHv3IcK21BQO31cc2H03mYT6ro-bvlxwUuSMxc9WF_M7_UKPxUf3-cX6M | duplicate |
| 32 | Zhao et al. 2017 | Zhao Y, Gong C, Wang Z, Zhang J, Wang L, Zhang S, et al. A randomized phase II study of aromatase inhibitors plus metformin in pre-treated postmenopausal patients with hormone receptor positive metastatic breast cancer. Oncotarget. 2017 Oct 13;8(48):84224–36. | duplicate |
