# Supplementary material for: Metformin for the treatment of breast cancer: a scoping review of randomized clinical trials
Source: BMC Cancer. 2025 Aug 21;25:1352. doi: 10.1186/s12885-025-14468-3 (PMC12369099; doi:10.1186/s12885-025-14468-3)
Supplement: Supplementary file 4 — Supplementary Material 4. Main characteristics of the 15 included studies without published results (as of September 2023). [file 12885_2025_14468_MOESM4_ESM.docx]

**Supplement 4:**  Main characteristics of the 15 included studies without published results (as of September 2023).

| Study / Registry number / Principal Intestigator | | Eligibility Criteria | Sample Size Goal | Intervention and Comparator | Outcomes | Informed Recruitment Period and Recruitment status |
| --- | --- | --- | --- | --- | --- | --- |
| **Study status: Prematurely ended** | | | | | | |
| NeoMET Study  (NCT01929811)  (Principal investigator (PI): Kunwei Shen) | | women aged ≥18 years and < 70 years with life expectancy > 12 months  Measurable disease in breast or axillary lymph node, histologically confirmed invasive breast cancer by core needle biopsy, T≥2cm or stage IIb or stage III according AJCC classification, fine-needle aspirationis encouraged to every patient with metastasis suspicious nodes;  Biopsy specimens are available for ER, PgR, Her2 and proliferation biomarker detection;  Adequate bone marrow function: Neutrophil ≥ 1.5*109/L; Hb ≥ 100g/L; PLT ≥ 80*109/L;  Adequate liver and renal function:  Serum AST ≤ 90U/L  Bilirubin ≤ upper limit of normal (UNL) range  Serum creatinine ≤110 umol/L，calculated creatinine clearance should be ≥ 60 mL/min;  BUN ≤ 7.1mmol/L;  Has ECOG Performance Score 0-1;  BMI ≥ 25kg/m2 or hyperglycemia or hyperlipemia or hypertension;  Willing to take biopsy before neoadjuvant chemotherapy and patients must be accessible for treatmentand follow-up;  Women with potential child-bearing must have a negative pregnancy test (urine or serum) within 7 daysof drug administration and agree to use an acceptable method of birth control to avoid pregnancy forthe duration of the study;  Written informed consent according to the local ethics committee requirements. | Actual Enrollment : 92 participants | **Interventions**:  TEC plus metformin Metformin (1500 mg/day)  **Comparator**: docetaxel, epirubicin and cyclophosphomide (TEC) | **Primary**:  pathologic complete response rate  **Secondary**:  1. Clinical response rate;  2. safety profile;  3. breast conservation therapy | **Time conduction:**  2013 - 2020  **Recruitment status:** Terminated (The study was stopped due to insufficient accrual) |
| NCT01477060  (PI: Milvia Zambetti) | | 1. Female patients with a histologically or cytologically confirmed adenocarcinoma of the breast progressing from prior hormonal therapy  2. Receptor positive disease (ER+ and/or PgR+)  3. HER2 negative  4. Pre- and post-menopausal status  5. Documented disease progression after first-line hormone therapy  6. Age ≥18 years.  7. Measurable or evaluable metastatic disease  8. Life expectancy > 3 months  9. ECOG Performance Status < 1  10. Adequate bone marrow, liver, and renal function as assessed by the following parameters:  Hemoglobin > 9.0 g/dl  Leucocytes count ≥ 3,000/mL  Absolute neutrophil count (ANC) ≥ 1.500/mL  Platelet count ≥ 100,000/mL  Alanine aminotransferase (ALT) and aspartate aminotransferase (AST) ≤ 2.5 x ULN (≤ 5 x ULN for patients with liver involvement)  Albumine and total bilirubin ≤ 1.5 x ULN  Prothrombin Time (PT) < 70 %  Serum creatinine < 1.4 mg/ml, creatinine clearance > 70 ml/min  11. Normal Respiratory Function and Saturation level ≥ 90%  12. New York Hearth Association (NYHA) Classification ≤ 2 and baseline left ventricular ejection fraction (LVEF)≥ 50%  13. Patients must be willing and able to sign a written informed consent. | Estimated enrollment: 69 patients (first step) and 168 patients (second step);  **Actual Enrollment:** 32 participants | **Interventions**:  Hormonal therapy + metformin (1500 mg/day) (arm B) and Hormonal therapy + lapatinib + metformin (arm C)  **Comparator:**  Hormonal therapy + lapatinib (arm A) | **Primary:**  To assess how many patients, after 3 months of therapy, are free of progressive disease  **Secondary:**  To assess the overall response rate; To assess the duration of response in patients who achieved partial or complete remission; To assess 3-years overall survival rate;  To assess tolerability of each proposed treatment; | **Time conduction:**  2011-2014  **Recruitment status:**  Terminated (The study was stopped due to insufficient accrual) |
| NCT02472353  (PI: Kirstin Williams) | | Breast cancer requiring neoadjuvant or adjuvant therapy with doxorubicin  Complete metabolic panel demonstrating adequate organ functions as defined by the following:  Aspartate transaminase (AST) less than 2.5 times Upper Limit of Normal (ULN); Alanine transaminase (ALT) less than 2.5 times ULN; alkaline phosphatase less than 2.5 times ULN; serum creatinine less than 1.5 mg/dL; serum bilirubin less than ULN  Eastern Cooperative Oncology Group (ECOG) performance status of 0 or 1  Age greater than or equal to 21 years | **Actual Enrollment**: 30 participants;  SAP: 44 (planned)  Both sexes were eligible for the study. | **Interventions**:  standard of care treatment with doxorubicin plus metformin (1700 mg/day)  **Comparator:**  standard of care treatment with doxorubicin alone | **Primary:**  Number of Participants With Less Than or Equal to 5% Decrease in Left Ventricle Ejection Fraction (LVEF) on Echocardiogram  **Secondary:**  LVEF of ≥10% from baseline; diastolic function; biomarkers (Troponin I, BNP, glutathione, circulating adiponectin, AMPK phosphorylation, and lipid peroxidation); exploratory genomic analysis. | **Time conduction:**  2014 – 2018  **Recruitment status:**  Terminated (The study was stopped due to insufficient accrual) |
| NCT02360059  (PI: David L. Ramirez) | | 1. Patients with histologically confirmed invasive breast cancer, stage I - IV, treated at Lyndon B. Johnson General Hospital in the Harris Health System.  2. Patients = or > 18 years old and < 75 years old.  3. Patients scheduled to undergo paclitaxel chemotherapy for breast cancer.  4. Patients with adequate renal function, as evidenced in laboratory values = or < 3 months old: epidermal growth factor receptor (eGFR) = or > 60 mL/min/1.73m2.  5. Patients with adequate hepatic function per institutional testing standards, as evidenced in laboratory values = or < 3 months old: (1) The screening results for total bilirubin must be < 1.5 times the upper limit of normal. (2) The screening results for aspartate aminotransferase (AST) and alanine aminotransferase (ALT) must be < 2 times the upper limit of normal.  6. Patients who speak English and/or Spanish.  7. Patients who are willing and able to review, understand, and provide written consent.  8. Patients with an Eastern Cooperative Oncology performance status of 0 or 1. | **Actual Enrollment**: 1 participant;  Both sexes were eligible for the study. | **Interventions**:  Metformin (2000 mg daily) + Questionnaires + Sensory and Fine-Motor Tests  **Comparator:**  Placebo + Questionnaires + Sensory and Fine-Motor Tests | **Primary:**  Mean Change in Neuropathy  **Secondary:**  NI | **Time conduction:**  2015-2016  **Recruitment status:**  Terminated (The study was stopped due to insufficient accrual) |
| **Study status: Not yet published** | | | | | | |
| OPTIMAL Trial  NCT04001725 and EudraCT 2019-000105-73  (Vernieri, 2020) | | 1. Age ≥ 18 years and ≤ 75 years  2. Histologically confirmed diagnosis of melanoma, lung (SCLC or NSCLC) or breast cancer  3. Recent (28 days), radiologically documented (contrast-enhanced CT or MRI) diagnosis of measurable brain metastases requiring treatment with high-dose dexamethasone (at least 8 mg daily for at least 21 days) plus/minus radiation therapy (RT).  4. Any previous or ongoing antitumor systemic therapy; patients who have never received previous systemic therapy can be also included.  5. Fasting glycemia < 126 mg/dl at the baseline evaluation or random glycemia of less than 200 mg/dl if the patient has not fasted for at least 8 hours before blood sampling.  6. Adequate blood tests:  Hemoglobin ≥ 9 g/dl  Absolute neutrophil count (ANC) in the range between 1.5-10 x 103/μl  Total bilirubin ≤ 1.5 times the upper normal limit (UNL). For patients with Gilbert syndrome or known liver metastases, bilirubin levels ≤ 3 times the UNL are considered acceptable  AST, ALT ≤ 3 times the UNL  Alkaline phosphatase ≤ 2.5 times the UNL  Serum creatinine concentration ≤ 1.5 x UNL  7. ECOG Performance Status ≤ 2  8. Life expectancy > 6 weeks  9. Written informed consent  10. Ability to swallow metformin tablets  11. Patients of female gender with the potential of childbearing (neither surgically sterile nor 2 years postmenopausal) must use a medically accepted method of contraception and must agree to continue use of this method for the duration of the study and for at least 60 days after study conclusion. Acceptable methods of contraception include double barrier method [i.e. condom and occlusive cap (diaphragm or cervical vault caps)] spermicide, intrauterine device (IUD), or steroidal contraceptive (oral, transdermal, implanted, or injected) in conjunction with a barrier method.  12. Patients of male gender having female partners with childbearing potential must use a medically accepted method of contraception and must agree to continue use of this method for the duration of the study and for 60 days after participation in the study. | Estimated Enrollment: 110 participants (both sexes) | **Interventions**:  high-dose dexamethasone plus metformin (2550  mg/day on day 7, if well tolerated)  **Comparator:**  high-dose dexamethasone | **Primary:**  the rate of precocious (14 days) dexamethasone-induced diabetes, as defined as fasting plasma glucose levels ≥ 126mg/dl.  **Secondary:**  Dexamethasone-induced diabetes at 30 days; Short-term mortality; Brain local control rate of disease; Patient ECOG performance status (PS); Patient Quality of Life (QoL); Absolute counts of immune cell populations; Relative counts of immune cell populations; Activation status of immune cell populations; Plasma lipids profile; Systemic inflammatory parameters; glucocorticoids-induced changes in gut microbiota populations; Metformin-induced changes in gut microbiota populations; Amino acid profile | **Time conduction:**  2019 – 2022  **Recruitment status:**  Recruiting |
| TCTR20200116007  (PI: Osataphan) | | 1) Female patient age > 18 years  2) Diagnosis with breast cancer and planned to receive doxorubicin  3) Given informed consent | Planned sample size: 396 | **Interventions**:  doxorubicin (chemotherapy) plus metformin (did not informed the dosage)  **Comparator**:  Arm 1: Placebo + doxorubicin  Arm 3: Donepezil + doxorubicin | **Primary**:  Troponin-I level about 2 weeks after completion of doxorubicin therapy  **Secondary**:  1. Cardiac function and biomarker at 2 weeks post doxorubicin, month 3, month 6, month 9, month 12  2. Markers of mitochondria dynamics, mitochondria function and apoptosis post chemo C2, C4, C6, 2 weeks post doxorubicin, Month 3, month 6, month 9, month 12  3. Genes associated with doxorubicin-induced cardiotoxicity at baseline | **Time conduction:**  2020 – 2022  **Recruitment status:**  Not yet recruiting |
| ChiCTR1900023487  (Zhang, 2020) | | 1) Premenopausal women aged 18-45 years old;  2) Breast cancer staging: patients receiving adjuvant/neoadjuvant chemotherapy in stage I-IIIa ER (-) PR (-) HER-2 (-) or HER-2 (+);  3) Assessment of normal ovarian function before chemotherapy (regular menstruation 2 months before chemotherapy);  4) Sign the informed consent form. | Planned sample size: 314 | **Interventions**:  Group 2: Metformin (2g/day ) + Chemotherapy  **Comparator**:  Group 1: Placebo + Chemotherapy  **Chemotherapy**: “For ER(-) PR(-) HER-2(-) breast cancer patients: EC-T (epirubicin/cyclophosphamide/paclitaxel) or AC-T (doxorubicin/cyclophosphamide/paclitaxel) chemotherapy regimen; "For HER-2(+) breast cancer patients: EC-TH (epirubicin/cyclophosphamide/paclitaxel/trastuzumab), AC-TH (doxorubicin/cyclophosphamide/paclitaxel/trastuzumab) or TCb-H (docetaxel/carboplatin/ docetaxel/trastuzumab) regimen. | **Primary**:  Menstruation recovery rate  **Secondary**:  Sex hormones+AMH+InhibinB; 5-year pregnancy outcome; Tumor prognosis (OS, ORR, DFS); The quality of patient's life; Safety assessment | **Time conduction:**  2019 - 2028 (planned)  **Recruitment status:**  Recruiting |
| ChiCTR1900027489  (PI: Wang) | | Breast cancer  1) Age: 18-45 years old;  2) Molecular typing of breast cancer: ER(-)PR(-)HER-2(-) or HER-2(+);  3) Breast cancer staging: patients receiving adjuvant/neoadjuvant chemotherapy in stage I-IIIa;  4) Assessment of normal ovarian function before chemotherapy (regular menstruation 2 months before chemotherapy);  5) Sign the informed consent form.  Cervical cancer  1) age: 18-45 years old;  2) Preoperative pathological diagnosis of cervical squamous cell carcinoma, cervical adenosquamous carcinoma or cervical adenocarcinoma;  3) Clinical diagnosis is IB2 (with fertility requirements), IB3 and IIA2;  4) retain one or both sides or part of the ovaries;  5) normal ovarian function before chemotherapy (regular menstruation 2 months before chemotherapy);  Sign the informed consent form.  Ovarian cancer  1) Patients aged 18-45 years old;  2) Pathological diagnosis is limited to unilateral ovarian epithelial cancer; malignant germ cell tumor (unlimited staging); IC stage stromal stromal cell tumor;  3) retain one or both sides or part of the ovaries;  4) Who receive chemotherapy;  5) Estimated survival period: more than 2 years; ECOG score is 0-1 points;  6) Normal ovarian function before chemotherapy (regular menstruation 2 months before chemotherapy);  7) Sign the informed consent form.  Trophoblastic tumors  1）age: 18-45 years old;  2) Clinical diagnosis of aggressive hydatidiform mole and choriocarcinoma (FIGO prognostic score >= 7 points, high-risk type), placental site trophoblastic tumor (PSTT) surgery to preserve ovarian patients, high risk factors (mitotic index > 5 / 10 HPF ; > 2 years from the previous pregnancy; deep infiltration, necrosis), patients with chemotherapy with EMA-Co regimen;  3) Erosive hydatidiform mole and choriocarcinoma (FIGO prognostic score <7, low-risk type), single-agent MTX chemotherapy showed drug resistance, and patients were treated with EMA-CO regimen;  4) Assessment of normal ovarian function before chemotherapy (regular menstruation 2 months before chemotherapy);  5) Sign the informed consent form. | Planned sample size: 234 | **Interventions**:  Metformin (dosage not informed) + Chemotherapy  **Comparator**: Placebo + Chemotherapy | **Primary**: Ovarian function recovery (Menstruation or AMH)  **Secondary**:  Sex hormones+AMH+InhibinB; 5-year pregnancy outcome;Safe assessment - Blood routine, urine routine, blood biochemistry, liver and kidney function, adverse reactions; Tumor prognosis; Disease free survival (DFS); Progression free survival (PFS | **Time conduction:**  2020 – 2023  **Recruitment status:**  Recruiting |
| ChiCTR-IPR-16008553  (PI: Guo-sheng) | | 1. Volunteered for this study and informed consent was obtained from the participants;  2. Female and age from 20 to 70 years old;  3. Diagnosis as breast cancer according to clinical practice guideline;  4. Indications for neoadjuvant chemotherapy according to clinical practice guideline: clinical I/II stage breast cancer;  5. Diagnosis as prediabetes: OGTT examinations show IFG 5.6-6.9mmol/L, IGT 7.8-11.0mmol/L; Or HbA1c:5.7-6.4%. | Planned sample size: 500 | **Interventions**:  Metformin (1700mg/day) + Chemotherapy  **Comparator**:  Placebo + Chemotherapy | **Primary**:  Pathologic complete response (PCR)  **Secondary**:  breast cancer events;  Clinical recession of lump;  Glucose intolerance and insulin function status;  Metabolic syndrome. | **Time conduction:**  2016 - 2021 (planned)  **Recruitment status:**  NI |
| SETUP AIM Trial  (ACTRN12612000416897)  (PI: Ganju) | | Stage 1 or 2 operable invasive breast cancer. Estrogen receptor positive (>10% cells)  Post-menopausal female  Histologically proven invasive adenocarcinoma of the breast through either a core needle biopsy or incisional biopsy. Excisional biopsy will not be allowed. Tumour must be confined to either the breast or to the breast and ipsilateral axilla. A complete metastatic staging work-up will be performed prior to definitive surgery, as clinically indicated.  Age > =18 years.  Karnofsky Performance status index > =80%.  Laboratory requirements: (within 28 days prior to registration)  Haematology:  Neutrophils > =1.5 x 109/L  Platelets >=100 x 109/L  Haemoglobin > =10 g/dL  Hepatic function:  Total bilirubin < =1 UNL (patients with a well documented history of Gilbert’s Syndrome are eligible)  ASAT (SGOT) and ALAT (SGPT) < =2.5 UNL  Alkaline phosphatase < =5 UNL  Renal function:  Creatinine <= 175 micromol/L (2 mg/dL)  Negative pregnancy test (urine or serum) within 7 days prior to registration for all women of childbearing potential.  Patients must be postmenopausal defined as:  Age >50 y and amenorrheic for 6 months or more  Age <50 y and amenorrheic for 12 months or more  Prior bilateral oophorectomy  Prior hysterectomy and has post menopausal levels of FSH and LH per local institutional standards  Age >55 y and prior hysterectomy | Sample size target: 40 | **Interventions**:  2 weeks of metformin (1g/day) followed by 2 weeks of metformin in addition to aromatase inhibitor in the lead up to your scheduled surgery.  **Comparator**:  2 weeks of metformin followed by 2 weeks of aromatase inhibitor alone : Arimidex or Femara | **Primary**:  Reduction in tumour tissue Ki67 level (proliferative index)  **Secondary**:  Imaging responses (mammogram, ultrasound, MRI) | **Time conduction:**  2012 - NI  The trial is registered on ANZCTR and it was last updated on 11/11/2015.  **Recruitment status:**  NI |
| MetBreCS Trial  (EUCTR2015-001001-14-IT) | | • Pre- and postmenopausal BC survivors (TN, or ER neg PgR neg Her2 pos, or Luminal B Her2 pos subtypes) who have completed any adjuvant therapy (not before 1 month from treatment cessation) without evidence of residual disease and with BMI > 25 kg/m2  • Female, (age > 18 years).  • Performance status = 0 (SWOG). | Planned number of subjects to be included: 236 | **Interventions**:  Metformin 850 mg alone  **Comparator**:  Placebo | **Primary**:  Change of Ki-67 in contralateral unaffected breast  **Secondary**:  To check the ability of the treatment to modulate various translational risk factors related to the target tissue and the host characteristics:  - circulating and molecular biomarkers  - metabolomic analysis  - gene expression profile in adipose and epithelial breast tissue  - assessment of epigenetic changes in methylome patterns (DNA methylation) | **Time conduction:**  2021 - 2024 (probably)  **Recruitment status:**  NI |
| HERMET Trial  (NCT03238495)  (PI: Qamar Khan) | | Eastern Cooperative Oncology Group Performance Status (ECOG PS) 0-1.  Unilateral or bilateral primary carcinoma of the breast, confirmed histologically by needle core biopsy.Fine-needle aspiration is not sufficient. Incisional/excisional biopsy is not allowed. In case of bilateralcancer, the investigator has to decide prospectively which side will be evaluated for the primaryendpoint.  Study participants must be cT1c - cT4a-d, any node (N), no metastases (M0). Any tumor (T) is allowedif node positive (biopsy proven and HER2 positive) including no primary invasive cancer or only ductalcarcinoma in situ (DCIS). Metastatic workup is not required.  Breast tumor must be >1.5 cm in maximum diameter by clinical or any radiologic measurement, if nodenegative. If node is positive by biopsy, study participant will be eligible regardless of the size of thebreast primary. In case of inflammatory breast cancer, the extent of inflammation/erythema can be usedas measurable lesion.  Multifocal or multicentric breast cancer is allowed if all the lesions are biopsied and are HER2 positive.Largest lesion will be assigned the target lesion.  Must be HER2-positive in primary breast tumor or lymph node by the ASCO/CAP guidelines 2013:http://www.asco.org/guidelines/her2  Ejection fraction (EF) greater than 50% by MUGA or ECHO within 4 weeks prior to first dose of studytreatment.  No prior cancer chemotherapy allowed.  Adequate organ and marrow function as defined below, unless deemed non-clinically significant andapproved by the Principal Investigator:Absolute neutrophil count ≥ 1,500/mcL  Platelets ≥ 100,000/mcl  total bilirubin within normal institutional limits  AST(SGOT) ≤ 2.5 X institutional upper limit of normal  ALT(SPGT) ≤ 2.5 X institutional upper limit of normal  ALK Phos ≤ 2.5 X institutional upper limit of normal  Creatinine clearance > 50mL/min  Women of child-bearing potential must agree to use adequate contraception (hormonal or barriermethod of birth control; abstinence) prior to study entry, for the duration of study participation, and for90 days following completion of therapy.  Negative pregnancy test within 14 days prior to randomization | Estimated Enrollment: 100 participants | **Interventions**:  TCH+P chemotherapy plus metformin  **Comparator**:  TCH+P chemotherapy only | **Primary**:  pCR  **Secondary**:  NI | **Time conduction:**  2017 - 2023 (estimated)  **Recruitment status:**  Recruiting |
| METNEO Trial  (NCT04170465)  (PI: Manar A Serageldin) | | Females ≥ 18 years of age and < 65 years.  Unilateral or bilateral primary carcinoma of the breast confirmed.  Eastern Cooperative Oncology Group Performance Status (ECOG PS) 0-2.  Clinically measurable tumor size who are candidates for neoadjuvant therapy.  No evidence of distant metastasis.  Normal renal and liver functions.  Non-diabetics. | Estimated Enrollment: 60 participants | **Interventions**:  AC-T neoadjuvant chemotherapy in addition to oral metformin HCl. 850 mg metformin tablets BID (1700 mg/day) daily until the completion of neoadjuvant chemotherapy cycles.  AC: (Doxorubicin 60 mg/m2 IV + cyclophosphamide 600 mg/m2 IV) for 4 cycles every 3 weeks followed by Taxol cycles (Paclitaxel 80 mg/m2 IV) once weekly for 12 weeks.  **Comparator**:  AC-T neoadjuvant chemotherapy alone  AC: (Doxorubicin 60 mg/m2 IV + cyclophosphamide 600 mg/m2 IV) for 4 cycles every 3 weeks followed by Taxol cycles (Paclitaxel 80 mg/m2 IV) once weekly for 12 weeks. | **Primary**:  1. Evaluation of the effect on tumor proliferation as measured by Ki-67 immunohistochemical (IHC)assessment (%) [ Time Frame: 6 months ]  Tissue level of Ki-67 expression in the excised tumor. Ki-67 will be scored as the percentage oftumour nuclei staining.  2. Evaluation of the effect on tumor apoptosis as measured by Caspase-3 [ Time Frame: 6 months ]  Tissue level of the active caspase-3 in the excised tumor.  3. Chemotherapy toxicities [ Time Frame: 6 months ]  Monitoring the incidence of chemotherapy toxicities during neoadjuvant therapy  **Secondary**:  pCR rate  [Time Frame: 6 months] | **Time conduction:**  2019 - 2020  **Recruitment status:**  Recruiting |
| BREAKFAST Trial  (NCT04248998)  (PI: Filippo de Braud) | | 1. Female sex  2. Age ≥ 18 and ≤ 75 years.  3. Evidence of a personally signed and dated informed consent document (ICD) indicating that the patient has been informed of all pertinent aspects of the study before enrollment  4. Willingness and ability to comply with the prescribed FMD regimen, metformin intake, the scheduled visits, treatment plans, laboratory tests and other procedures.  5. Histologically confirmed diagnosis of invasive TNBC candidate to neoadjuvant chemotherapy and subsequent curative surgery. On the basis of International Guidelines, TNBC is defined by absent or minimal (<1%) expression of oestrogen and progesterone receptors at IHC, and absence of  HER2 over-expression or amplification, as defined as an IHC score of 0, 1+, or an IHC score of 2+ with in situ hybridization (ISH) analysis excluding HER2 gene amplification.  6. Patients with localized disease (clinical stage I-III according to TNM). Patients with Stage I TNBC will be included only if the primary tumor is at least 10 mm in greatest dimension (clinical T1c as determined through baseline MRI assessment).  7. Presence of an Eastern Cooperative Oncology Group (ECOG) performance status 0 or 1.  8. Presence of adequate bone marrow and organ function as defined by the following laboratory values:  a. ANC ≥ 1.5 x 103/l  b. platelets ≥ 100 x 103/l  c. hemoglobin ≥ 9.0 g/dl  d. calcium (corrected for serum albumin) within normal limits or ≤ grade 1 according to NCICTCAE  version 5.0 if not clinically significant  e. potassium within the normal limits, or corrected with supplements  f. creatinine < 1.5 ULN  g. blood uric acid < 10 mg/dl  h. ALT and AST ≤ 2 x ULN  i. total bilirubin < 1.5 ULN except for patients with Gilbert syndrome who may only be included if the total bilirubin is < 3.0 x ULN or direct bilirubin < 1.5 x ULN  j. Fasting glucose ≤ 250 mg/dl.  9. Female patients of childbearing potential must agree to sexual abstinence or to use two highly effective methods of contraception throughout the study and for at least six months after the end of the FMD. Abstinence is only acceptable if it is in line with the preferred and usual lifestyle of the patient. Examples of contraceptive methods with a failure rate of < 1% per year include tubal  ligation, male sterilization, hormonal implants, established, proper use of combined oral or injected hormonal contraceptives, and certain intrauterine devices. Alternatively, two methods  (e.g., two barrier methods such as a condom and a cervical cap) may be combined to achieve a failure rate of < 1% per year. Barrier methods must always be supplemented with the use of a spermicide. A patient is of childbearing potential if, in the opinion of the Investigator, she is biologically capable of having children and is sexually active.  10. Female patients are not of childbearing potential if they meet at least one of the following criteria:  a. Have undergone a documented hysterectomy and/or bilateral oophorectomy  b. Have medically confirmed ovarian failure  c. Achieved post-menopausal status, defined as: ≥ 12 months of non-therapy-induced amenorrhea or surgically sterile (absence of ovaries) and have a serum FSH level within the laboratory's reference range for postmenopausal females. | Estimated Enrollment: 90 participants | **Interventions**:  Arm B will consist of 6 months of standard anthracycline-taxane preoperative chemotherapy in combination with triweekly cycles of 5-day FMD and daily metformin (1700 mg/day).  **Comparator**:  Chemotherapy plus Fasting-Mimicking Diet (FMD) | **Primary**:  pCR rate  **Secondary**:  1. Severe adverse events  2. Safety of the experimental treatments  3. Compliance with the experimental treatment  4. Relapse-free survival (RFS)  5. Distant metastasis-free survival (DMFS)  6. Overall Survival (OS)  7. Short-term modifications of plasma glycemia (mg/dl)  8. Long-term modifications of plasma glycemia (mg/dl)  9. Short-term modifications of serum insulin concentration (μU/ml)  10. Long-term modifications of serum insulin concentration (μU/ml)  11. Short-term modifications of serum IGF-1 concentration (ng/ml)  12. Long-term modifications ofserum IGF-1 concentration (ng/ml)  13. Short-term modifications of blood lipid profile by UPLC-MS and HPLC-ELDS  14. Long-term modifications of blood lipid profile by UPLC-MS and HPLC-ELDS  15. Clinical tumor response  16. Gene expression profiles  17. Mutational analyses  18. Short-term modifications of plasma amino acid profile by UPLC-QDa Mass detector system (Waters)  19. Long-term modifications of plasma amino acid profile by UPLC-QDa Mass detector system (Waters) | **Time conduction:**  2020 - 2024 (estimated)  **Recruitment status:**  Recruiting |
| NCT04387630  (PI: Osama Hussein) | | Histologically proven breast carcinoma.  American Society of Anesthesiology (ASA) score I-II.  Candidate for neoadjuvant chemotherapy as per hospital's protocol.  Clinically measurable tumor.  No evidence of distant metastasis.  Normal renal and liver functions.  Non-diabetics. | Estimated Enrollment: 120 participants | **Interventions**:  Metformin (2550 mg/day) + Chemotherapy (AC-T or AC)  **Comparator**:  Placebo + Chemotherapy (AC-T or AC) | **Primary**:  Clinical Response rate  **Secondary**:  T-cell cytotoxic markers | **Time conduction:**  2020 – 2023 (estimated)  **Recruitment status:**  Recruiting |
| NCIC CTG MA.32  (Goodwin, 2022)  (substudies) | NCT01286233  (PI: Norman Wolmark) | The patient must have consented to participate and must have signed and dated an appropriate IRB-approved consent form that conforms to federal and institutional guidelines for the MA.32.F Study before being enrolled.  The patient must be female.  The patient must reside in the United States or Canada.  The patient must be English-speaking.  The patient must be eligible for randomization in the MA.32 treatment trial. (Participation in the MA.32QOL study is permitted but not required.)  The patient must not have started taking MA.32 study therapy.  The patient must have completed primary breast radiation therapy at least two weeks prior toenrollment in MA.32.F. | Actual  Enrollment: 394 female participants | **Interventions**:  metformin 850 mg caplet po bid for 5 years, including a 4-week ramp-up of one caplet per day  **Comparator**:  an identical placebo po bid for 5 years, including a 4-week ramp-up of one caplet per day | **Primary**:  1. Questionnaire scores from patient reported outcome battery regarding fatigue, stress, sleep,depression, and general quality of life [ Time Frame: Collected at baseline and 6, 12, 24, and 36months from randomization ]  2. Questionnaire scores from patient reported comorbid conditions and behavioral risks[ Time Frame: Collected at baseline and 6, 12, 24, and 36 months from randomization ]  3. Biological correlates of fatigue (medical and demographic characteristics of pts.) [ Time Frame: Collected at baseline and 6, 12, 24, and 36 months from randomization ] Biological correlates (medical) will be collected at these timepoints. Standard demographics will becollected at baseline only.  4. DNA polymorphisms [ Time Frame: Collected at baseline ]  5. Changes in RNA gene expression [ Time Frame: Collected at baseline and 6, 12, and 24 months ]  **Secondary**:  NI | **Time conduction:**  2011 – 2016  **Recruitment status:**  Unknown |
|  | Wood, 2013 | Patients must either be concurrently enrolling or previously enrolled to Canada (CAN) National Cancer Institute of Canada (NCIC) study MA.32 (CAN-NCIC-MA.32) (MA.32); eligible patients may be either pre- or post-menopausal  Patients must have hormone receptor-negative breast cancer  Patients must have breast density ≥ 25% (correlating with the Breast Imaging-Reporting and Data[BIRAD]-2 category of "scattered fibroglandular densities" or greater)  Baseline digital mammograms taken within 12 months prior to registration to MA.32, with at least acraniocaudal (CC) view used for enrollment to MA.32 must be available for submission; if the patienthas previously enrolled to MA.32 and one year has elapsed from baseline mammograms, one-yearmammograms must also be available for submission  Contralateral unaffected breast in place (with no prior cancer or radiation, no implants, and no plan forbreast surgery on contralateral breast over the course of the study); women with a prior biopsy on the unaffected breast are eligible | Estimated Enrollment: 458 female patients | **Interventions**:  metformin 850 mg caplet po bid for 5 years, including a 4-week ramp-up of one caplet per day  **Comparator**:  an identical placebo po bid for 5 years, including a 4-week ramp-up of one caplet per day | **Primary**:  Change in percent mammographic breast density in contralateral (unaffected) breast from baseline to 1 year  **Secondary**:  1. Change in percent mammographic breast density in contralateral (unaffected) breast from baseline to 2 years using two-sample t-test  2. Correlation between baseline mammographic density and baseline plasma fasting insulin,glucose levels, and HOMA using a scatter plot, correlation-coefficient estimation, and linear-regression method  3. Correlation of changes in dense area in response to metformin therapy from pre-treatment to ontreatment (at year 1 and year 2) with plasma fasting insulin, glucose levels, and HOMA using simple linear-regression method  4. Correlation between mammographic density and the incidence of second primary breast cancer using correlation coefficient and scatter plot | **Time conduction:**  2012 – 2013  **Recruitment status:**  Unknown |
